# Supramolecular protection of isocyanates from water by encapsulation within hydrophobic crystalline pillar[*n*]arene macrocycles

**DOI:** 10.1038/s41467-026-72696-y

**Published:** 2026-05-09

**Authors:** Kiichi Yasuzawa, Kouhei Sutou, Katsuto Onishi, Shunsuke Ohtani, Kenichi Kato, Hiroshi Yamazaki, Junko N. Kondo, Shigehisa Akine, Tomoki Ogoshi

**Affiliations:** 1 Department of Synthetic Chemistry and Biological Chemistry, Graduate School of Engineering, Kyoto University, Kyoto, Japan; 2 School of Materials and Chemical Technology, Tokyo Institute of Technology, Yokohama, Japan; 3 Chemical Resources Laboratory, Tokyo Institute of Technology, Yokohama, Japan; 4 Institute Management, Office of Communication and DEI, Institute of Science Tokyo, Yokohama, Japan; 5 Graduate School of Natural Science and Technology, Kanazawa University, Kanazawa, Japan; 6 WPI Nano Life Science Institute, Kanazawa University, Kanazawa, Japan

**Keywords:** Crystal engineering, Self-assembly, Self-assembly

## Abstract

The high reactivity of isocyanate groups not only makes them excellent precursors for polyurethanes and polyureas, but it also leads to their degradation through reactions with atmospheric moisture. Conventionally, blocking reagents containing alcohol or amine groups have been used to protect isocyanate groups. However, such covalent protection approaches often require harsh deprotection conditions. Herein, we report a non-covalent supramolecular strategy that provides both high protection efficiency and facile deprotection of isocyanate compounds. In this approach, the isocyanate groups are encapsulated within crystalline pillar[*n*]arene macrocycles. Owing to the highly hydrophobic nature of the pillar[*n*]arene crystals, the isocyanate guests are effectively protected from water in the crystalline state: the isocyanate groups are active even after the crystalline complexes are exposed to water vapour or directly immersed in water. Furthermore, although the host–guest complex is stabilized in the crystalline state, simple dissolution in an appropriate reaction solvent simultaneously triggers deprotection, enabling subsequent polyurethane synthesis.

## Abstract (web summary)

Isocyanate groups are excellent precursors for the synthesis of polyurethanes and polyureas however they degrade with atmospheric moisture. Here, the authors report the encapsulation of isocyanates within crystalline pillar[*n*]arene macrocycles that provides both high protection efficiency and facile deprotection of isocyanate compounds.

## Introduction

High reactivity of chemical compounds is a double-edged sword. On one hand, it makes the chemical an excellent synthetic substrate. On the other hand, the chemical easily undergoes side reactions and becomes degraded. Naturally, the time these compounds are stored is much longer than the time they are actually used in chemical reactions. Therefore, preventing their degradation during storage is a critical issue that cannot be overlooked.

To overcome this problem, chemists have developed a variety of strategies to store highly reactive chemicals in water and/or air. A representative example is the encapsulation of organometallic compounds within paraffin capsules^1,2^ or organogels^3–5^. Because these methods effectively prevent the direct contact of the compounds with the external environment, their stability is maintained even when the compounds are exposed to air. However, reactive species located at the gel surface remain susceptible to degradation, leaving challenges for stabilisation under harsher conditions, such as in water. These issues should be addressed by switching to media with lower fluidity, such as solids or crystalline materials, that are less affected by external stimuli than waxes or gels. As another approach, supramolecular host molecules and cages have been used to stabilise guest molecules through host–guest interactions, and the guests are effectively isolated from the external environment^6–17^. Notably, because the stabilisation occurs at the molecular level, this strategy can ensure uniform protection. Nevertheless, the strong host–guest association often requires the addition of competitive guests to release the encapsulated compounds. Therefore, we envisioned that if a system could be designed in which host–guest complexes form only in the solid state—where weak interactions are still sufficient for association, but not in solution due to solvation effects—it would allow strong protection of reactive guests from water in the solid state while enabling their rapid release and recovery of reactivity upon dissolution.

Among highly reactive species, compounds containing isocyanate (NCO) groups are essential in industry^18^. Isocyanates readily react with hydroxyl (OH) or amine (NH_2_) groups, and they are widely used as precursors for polyurethanes and polyureas. However, isocyanates also easily react with atmospheric moisture in the air and are converted into amines through the equation

1 $${-}{{\rm{NCO}}}+{{\rm{H}}}_{2}{{\rm{O}}}\to {-}{{\rm{NH}}}_{2}+{{\rm{CO}}}_{2}$$

preventing the desired reactions with OH or NH_2_ groups^19^. Therefore, great care is required to avoid exposure to moisture during the storage of isocyanates.

Traditionally, chemical modification has been used to protect isocyanate groups. In this approach, a stable urethane (or urea) bond is formed between the isocyanate group and an alcohol (or amine) group, and the protected intermediate is deprotected before use. Such covalently protected species are known as blocked isocyanates, and they exhibit excellent stability against water^20–24^. However, because the protection involves covalent bonding, harsh conditions are often required to regenerate the active isocyanate groups, such as high temperatures. In addition, the blocking reagent (typically an alcohol or an amine) may remain in the system after deprotection, potentially leading to undesired side reactions. These limitations highlight the inherent trade-off between strong protection and facile deprotection.

**Fig. 1:**
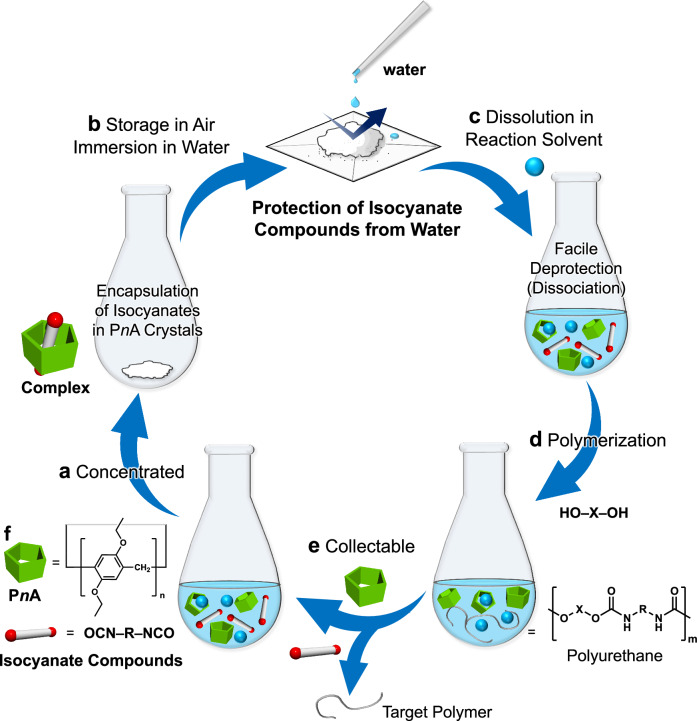
Concept of supramolecular protection of isocyanate compounds using hydrophobic crystals of pillar[*n*]arene (P*n*A) macrocycles. **a** A mixed solution of P*n*A and an isocyanate compound is concentrated to obtain the P*n*A–isocyanate co-crystal. **b** Owing to the hydrophobicity of the P*n*A crystals, the co-crystal is stable not only in air and moisture, but also in water, resulting in the protection of the isocyanate compound. **c** Because the binding affinity of the complex is weak in the reaction solvent, the complex immediately dissociates, indicating a facile deprotection process. **d** Polyurethane synthesis then occurs by the reaction with diol. **e** P*n*A can be collected from the reaction mixture. **f** Chemical structure of P*n*A.

In this work, we achieved both high storage stability and mild deprotection by utilising non-covalent host–guest interactions between pillar[*n*]arene macrocycles and isocyanate compounds. Pillar[*n*]arenes^25–28^ are pillar-shaped macrocycles composed of benzene rings connected at the para positions via methylene bridges (Fig. 1f). Notably, owing to their electron-rich π-cavities, pillar[*n*]arenes can incorporate guest molecules through bulk mixing of crystalline pillar[*n*]arenes with neat guests or by concentrating host–guest solutions via evaporation, driven by multipoint C–H···π and C–H···O interactions^28–37^. Because most organic molecules possess C–H groups, pillar[*n*]arenes are capable of incorporating a wide range of guest molecules that match the cavity size, specifically in the crystalline state, even when complexation does not occur in a solution. Furthermore, this study revealed that crystals of pillar[*n*]arene with ethoxy side chains (Fig. 1f, *n* = 5, P5A; *n* = 6, P6A) exhibit no water adsorption. Therefore, we envision that the guest molecules encapsulated within the crystalline pillar[*n*]arene would be effectively protected from water (Fig. 1). To demonstrate this concept, we prepared host–guest crystals of pillar[*n*]arenes and isocyanate compounds via evaporation of a solution containing the host and the guest (Fig. 1a). Remarkably, the isocyanate groups remained stable even after the crystals were exposed to water vapour or immersed in water (Fig. 1b). Importantly, although the host–guest complex is stabilised in the crystalline state, dissolution in an appropriate solvent rapidly induces dissociation of the complex, enabling facile deprotection (Fig. 1c). Consequently, polyurethane synthesis can be successfully achieved simply by dissolving the complex in the reaction solvent without additional deprotection steps (Fig. 1d). Additionally, P*n*A can be collected from the reaction solution by reprecipitation (Fig. 1e). Thus, our supramolecular protection approach using hydrophobic pillar[*n*]arene crystals successfully overcomes the trade-off between strong protection and facile deprotection faced by conventional covalently blocked isocyanates.

## Results

### Preparation of the host–guest complex of pillar[5]arene and hexamethylene diisocyanate

**Fig. 2:**
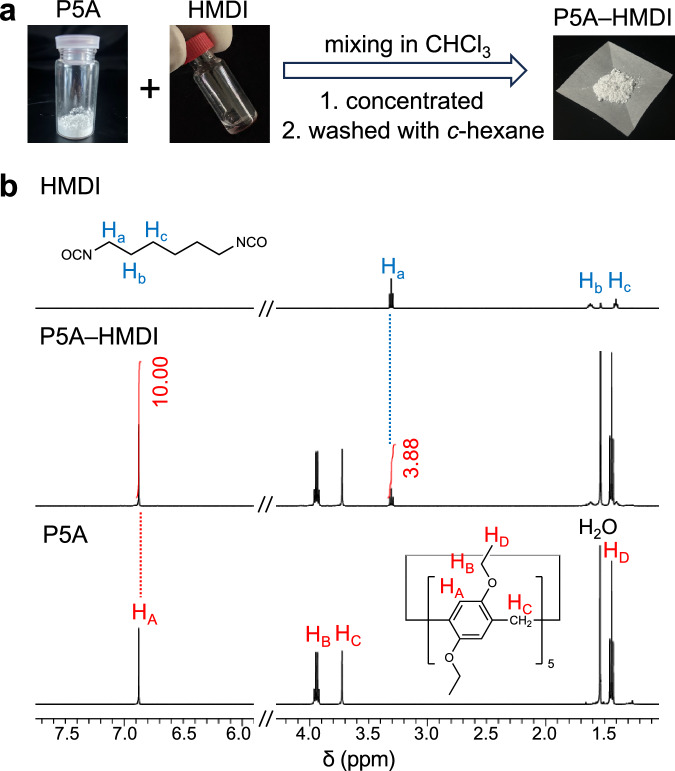
Preparation of the crystalline host–guest complex of pillar[5]arene (P5A) and hexamethylene diisocyanate (HMDI). **a** Schematic outline of the preparation of the complex crystal. P5A and HMDI were dissolved in chloroform at a 1:1.1 molar ratio. After the solution was concentrated, the crystal was washed with cyclohexane to remove uncomplexed HMDI. The crystalline P5A–HMDI complex was obtained as a white solid. **b** Chemical structures of HMDI and P5A and ^1^H NMR spectra (dichloromethane-*d*_2_, 25 °C, 500 MHz) of HMDI, P5A–HMDI, and P5A. Once the crystalline P5A–HMDI complex was dissolved in dichloromethane-*d*_2_, no host–guest complexation was observed. However, 1:1 stoichiometry was confirmed from the integral ratio of an aromatic proton of P5A (H_A_) to a methylene proton of HMDI (H_a_).

We first used hexamethylene diisocyanate (HMDI) as the target isocyanate compound and pillar[5]arene (P5A) crystal as the supramolecular protector (Fig. 2). Since the cavity size of P5A (4.7 Å) is suitable for linear alkyl chain, a host–guest complex of P5A and HMDI was prepared by simply concentrating a chloroform solution of the two components in 1–1.1 molar ratio (Fig. 2a, details in Methods). Because chloroform is bulkier than the cavity of P5A, it does not compete with HMDI for inclusion in solution or during crystallisation, allowing preferential encapsulation of HMDI during crystal formation. The obtained solid was then washed with cyclohexane to remove uncomplexed HMDI because bulky cyclohexane does not compete with the guest of P5A and can dissolve only HMDI. Proton signals from both P5A and HMDI were observed by ^1^H NMR in dichloromethane-*d*_2_ (Fig. 2b) even after uncomplexed HMDI was removed with cyclohexane, indicating the complexation of P5A and HMDI. Unlike chloroform, dichloromethane acts as a competitive guest solvent and rapidly dissociates the P5A–HMDI complex; therefore, the resulting integral ratio reflects the host–guest stoichiometry in the crystalline complex. Based on the integrals of an aromatic proton of P5A (H_A_, 10H) and a methylene proton of HMDI (H_a_, 4H), 1:1 complexation was confirmed. Importantly, the complex can be prepared on the gram scale without requiring a large excess of either P5A or HMDI, demonstrating its potential for practical application and further developments (details in Methods). Thermogravimetric analysis of the complex showed 16% weight loss at approximately 230 °C (Supplementary Fig. 14b). This corresponded to 1 equivalent of HMDI, supporting the formation of a 1:1 complex. The powder X-ray diffraction (PXRD) profile of the obtained solid showed disappearance of the peaks corresponding to individual P5A and the appearance of a new set of reflections, indicating formation of a crystalline host–guest complex.

### Stability of the crystalline host–guest complex of P5A and HMDI against water and temperature

**Fig. 3:**
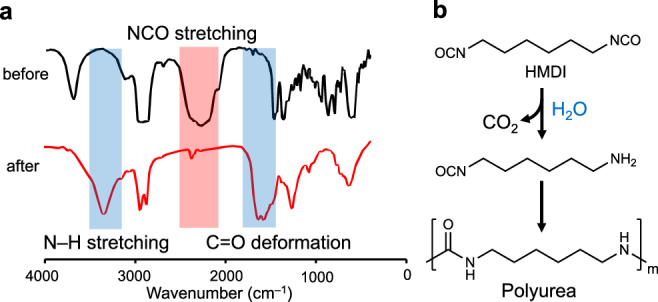
Investigation of the stability of neat HMDI without encapsulation by P5A. **a** Fourier transform infrared (FT-IR) spectra of neat HMDI before and after water vapour exposure. The signal around 2300 cm^–1^ in the after spectrum is attributed to CO_2_. **b** Plausible degradation scheme of HMDI under moisture. HMDI readily reacts with water vapour, undergoing hydrolysis to form amine groups. The resulting amine group can subsequently react with residual isocyanate group, yielding insoluble polyurea.

We first exposed neat HMDI to water vapour (Supplementary Fig. 6). After 3 days, a white product was obtained that was hardly soluble in a wide range of solvents, including water, DMSO, DMF, MeOH, acetone, THF, dichloromethane, chloroform, and *n*-hexane. The Fourier transform infrared (FT-IR) spectrum of the product showed the disappearance of the characteristic NCO stretching band at approximately 2300 cm^−1^, along with the appearance of urea signals, specifically the N–H stretching vibration at approximately 3300 cm^−1^ and the C=O deformation vibration at approximately 1600 cm^−1^ (Fig. 3a). These results indicate that neat HMDI readily reacts with water vapour, whereby the isocyanate groups are hydrolysed to form amine groups. These newly formed amine groups then undergo further reaction with the residual isocyanate groups, resulting in the formation of insoluble polyurea (Fig. 3b).

**Fig. 4:**
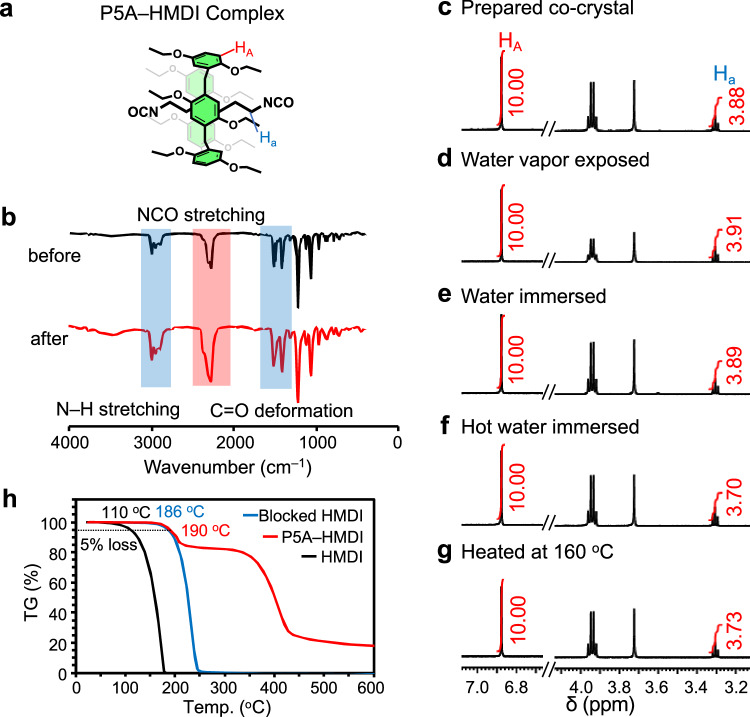
Investigation of the stability of the supramolecularly protected isocyanate compound. **a** Chemical structure of the P5A–HMDI complex. **b** Fourier transform infrared (FT-IR) spectra of the crystalline P5A–HMDI complex before and after water vapour exposure. ^1^H NMR spectra (dichloromethane-*d*_2_, 25 °C, 500 MHz) of the P5A–HMDI complex: as prepared (**c**), after 1 month of water vapour exposure (**d**), after 1 month of immersion in water (**e**), after 1 h of immersion in hot water (**f**), and after 1 h of heating at 160 °C (**g**). The integral ratio of an aromatic proton of P5A (H_A_) to a methylene proton of HMDI (H_a_) was used to evaluate the degree of isocyanate degradation. **h** Thermogravimetric (TG) curves of blocked HMDI, the crystalline P5A–HMDI complex, and neat HMDI at a heating rate of 10 °C/min under a flow of dry nitrogen. Blocked HMDI refers to HMDI in which both terminal isocyanate groups are protected by *ε*-caprolactam units.

Next, a similar experiment was conducted using the crystalline P5A–HMDI complex. After more than 1 month exposure of the crystalline complex to water vapour, no observable changes occurred, and no insoluble product formed (Supplementary Fig. 7). In the ^1^H NMR spectra (Fig. 4c, d), the integral ratio of an isocyanate proton (H_a_) to an aromatic proton of P5A (H_A_) remained almost unchanged (from 3.88 to 3.91), indicating the continued presence of isocyanate groups. The NCO signal at approximately 2300 cm^−1^ was also observed in the FT-IR spectrum of P5A–HMDI (Fig. 4a). Additionally, the signals corresponding to urea were barely detectable. These NMR and FT-IR results suggest that most of the isocyanate groups were successfully protected by encapsulation within the P5A crystal.

We further investigated their protection under more severe conditions, such as direct immersion in water and boiling water. The crystalline P5A–HMDI complex was placed in a vial with water, and the vial was sealed. After storing the sample for 1 month, ^1^H NMR and FT-IR analyses were performed to evaluate the retention of the isocyanate groups. Remarkably, even after 1 month of water immersion, no insoluble product was observed. The ^1^H NMR spectra (Fig. 4c, e) showed that the integral ratio of an HMDI proton (H_a_) to an aromatic proton of P5A (H_A_) remained nearly unchanged (from 3.88 to 3.89). In the FT-IR spectrum, the characteristic NCO stretching band at approximately 2300 cm^−1^ was clearly observed, while no urea signals (N–H stretching signal at approximately 3300 cm^−1^ and C=O deformation signal at approximately 1600 cm^−1^) were detected, indicating that the isocyanate groups remained intact (Supplementary Fig. 10). Following immersion of the crystalline complex in boiling water at 100 °C for 1 h, the ^1^H NMR spectra (Fig. 4c, f) showed a slight decrease in the integral ratio of HMDI proton A (from 3.88 to 3.70), and urea signals (N–H stretching vibration at approximately 3300 cm^−1^ and C=O deformation vibration at approximately 1600 cm^−1^) were observed in the FT-IR spectrum (Supplementary Fig. 12), suggesting perfect protection was not achieved. However, these results indicate that most of the isocyanate groups were still protected from degradation in the crystalline state (95% from ^1^H NMR integral ratio, Supplementary Fig. 11), even after immersion in boiling water. This high level of stability under such harsh conditions highlights the effectiveness of this supramolecular protection approach in crystalline state.

In addition to water stability, thermal stability is also a crucial factor for the storage of isocyanate compounds^38^. Although HMDI has a boiling point of 255 °C, thermogravimetric analysis revealed that it begins to volatilise and decompose at approximately 70 °C, with 5% weight loss observed at 110 °C, and it is completely decomposed by 180 °C (Fig. 4h). In contrast, a blocked isocyanate (blocked HMDI), whose terminal isocyanate groups are protected by two *ε*-caprolactam units (Supplementary Fig. 14a), exhibited significantly higher thermal stability, with 5% weight loss at 186 °C, owing to the formation of strong urea bonds. Notably, the P5A–HMDI complex showed 5% weight loss at 190 °C, slightly surpassing that of the blocked isocyanate. Even after the crystalline P5A–HMDI complex was heated at 160 °C for 1 h, the presence of the isocyanate groups was confirmed by ^1^H NMR measurement (Fig. 4g). It is remarkable that, even though the P5A–HMDI complex only uses non-covalent interactions, the thermal stability is equivalent to that of covalent-bonding blocked isocyanates.

### Hydrophobicity of P5A

To clarify the reason why the isocyanate compound was effectively protected from water, we measured the adsorption behaviour of water using crystalline P5A without any guest molecules in its cavity. In contrast to *n*-hexane vapour, for which P5A can incorporate one equivalent, the water adsorption and desorption isotherms remained nearly flat (Supplementary Fig. 4). These results indicate that no water was adsorbed in the P5A crystal. In addition to these measurements, FT-IR spectroscopy was performed under water vapour exposure. Interestingly, the FT-IR difference spectrum between P5A and P5A exposed to water vapour showed only minimal changes, confirming that no water adsorbed in P5A (Supplementary Fig. 3). In addition, water droplets retained their spherical shape on the surface of crystalline P5A (Supplementary Fig. 5a). The surface hydrophobicity was evaluated by measuring the contact angle of a thin P5A film (Supplementary Fig. 5b). The measured contact angle was 81°, indicating that P5A possesses a hydrophobic surface. This hydrophobicity would come from the aromatic backbone and alkoxy substituents on the side chains. These results demonstrate that P5A provides a highly hydrophobic environment in the crystalline state, enabling the effective protection of guest molecules from water.

### Single-crystal structure of the P5A–HMDI complex

**Fig. 5:**
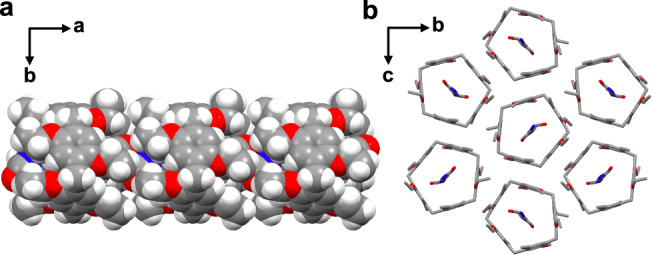
Investigation of the stability of the supramolecularly protected isocyanate compound. X-ray crystal structure of the P5A–HMDI complex in the *ab* plane (**a**, space-filling model) and *bc* plane (**b**, capped sticks model, the hydrogen atoms have been removed for clarity).

Crystallographic data can provide structural evidence of the protection of the isocyanate guest molecule within the P5A crystal. Single crystals of the P5A–HMDI complex were obtained by vapour diffusion of cyclohexane into a chloroform solution of P5A and an excess amount of HMDI. X-ray crystallographic analysis revealed that the HMDI molecule was encapsulated within the cavity of P5A at a 1:1 molar ratio, consistent with the integral ratio observed in the ^1^H NMR spectrum (Fig. 4c), and that the P5A units assembled into a one-dimensional (1D) channel structure (Fig. 5a, b). Notably, space-filling models showed that there was little to no room for water molecules to access the encapsulated isocyanate guests within the P5A channels. This structural observation supports the high water stability of the isocyanate groups, which is attributed to their encapsulation within the hydrophobic P5A crystal lattice. The experimental PXRD pattern is consistent with that simulated from the single-crystal X-ray structure of the P5A–HMDI complex (Supplementary Fig. 2). Furthermore, the formation of 1D channel structure would be one of the key factors contributing to the improved thermal stability. Based on our previous studies, molecules confined within the 1D channels of P5A crystals exhibit significantly restricted molecular motion compared with the neat molecules because they fit into the P5A channels^32^. Consequently, the confined molecules show enhanced thermal stability. In the present study, we therefore consider that confinement of the isocyanate species within the 1D channel structure of the P5A crystal is responsible for the remarkable improvement in thermal stability observed in the crystalline state, which was comparable to the typically covalent stabilisation.

### Removal of the supramolecular protecting host in solution

We next investigated the deprotection process of the supramolecularly protected HMDI. The deprotection behaviour can be readily controlled by appropriate solvent selection. For example, in systems based on P5A, dichloromethane, whose molecular size matches the cavity of P5A, is known to induce competitive inclusion and thereby rapidly release guests encapsulated in the crystalline state. No chemical shift differences of the HMDI protons were observed between a dichloromethane-*d*_2_ solution of the neat HMDI and that of the P5A–HMDI mixture (Fig. 2b). This indicates that host–guest complexation hardly occurs in dichloromethane-*d*_2_. Although P5A and HMDI can form the host–guest complex in the crystalline state, HMDI was immediately released upon dissolving in the dichloromethane, indicating that the deprotection was instantly completed. Deprotection was also confirmed to proceed in other solvents such as chloroform and toluene (Supplementary Figs. 15 and 16 and Supplementary Notes 1 and 2). Conventional covalently blocked isocyanates require harsh conditions, such as high temperatures, for deprotection because of their strong protective capability. In contrast, our supramolecular protection system achieves both high stability and mild deprotection conditions, thereby resolving the longstanding trade-off between protection strength and ease of deprotection.

### Polyurethane synthesis

**Fig. 6:**
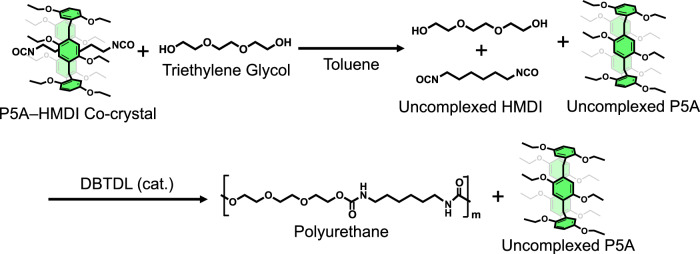
Polyurethane synthesis from triethylene glycol and supramolecularly protected diisocyanate. Synthetic scheme for the polymerisation of triethylene glycol and HMDI using the crystalline P5A–HMDI complex. Upon dissolution of the complex in toluene, HMDI is released from supramolecular protection owing to the very weak binding in the solution state. In the presence of the dibutyltin dilaurate (DBTDL) catalyst, HMDI reacts with triethylene glycol to afford polyurethane. The target polymer is obtained as a white precipitate, while uncomplexed P5A remains in the toluene solution and can be recovered.

One of the important roles of isocyanate groups is as a precursor for polyurethane synthesis. Therefore, we attempted to synthesise polyurethane from supramolecularly protected HMDI (Fig. 6). The crystalline P5A–HMDI complex and triethylene glycol were dissolved in toluene and stirred at 90 °C for 3 h in the presence of a dibutyltin dilaurate (DBTDL) catalyst. After the reaction, a white solid precipitated. The toluene phase was removed, and the precipitate was dissolved in dimethyl sulfoxide-*d*_6_ for ^1^H NMR analysis. The ^1^H NMR spectra clearly showed proton signals corresponding to urethane linkages, indicating successful polymerisation (Supplementary Fig. 17).

Importantly, deprotection of the isocyanate was achieved simply by dissolving the crystalline complex in the reaction solvent at the reaction temperature (Supplementary Fig. 16 and Supplementary Note 2). Unlike dichloromethane, the bulkier toluene does not effectively induce competitive inclusion with P5A and therefore provides slower release kinetics; however, dissociation of the complex proceeded sufficiently under the reaction conditions. These results show that this supramolecular protection strategy is readily applicable to conventional polymerisation processes, without the need for additional steps to reactivate the isocyanate groups. Moreover, the solid form of the supramolecularly protected HMDI significantly improves handling, because volatilisation and degradation can be avoided even under ambient conditions. Furthermore, because P5A is dissolved in the toluene phase after the reaction, P5A can be easily collected by reprecipitation (85% recovery), indicating the potential for the development of an eco-friendly system (Fig. 1e, Supplementary Fig. 20).

### Other isocyanates

**Fig. 7:**
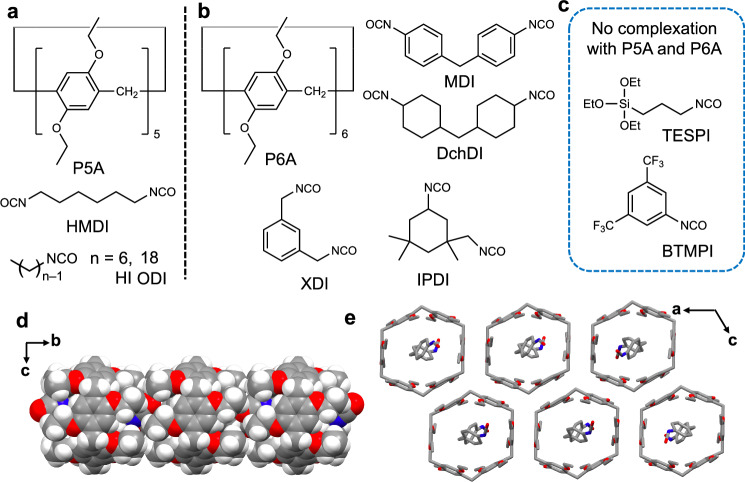
Protection of other isocyanate compounds from water. **a** P5A was used as the host macrocycle of hexamethylene diisocyanate (HMDI), hexyl isocyanate (HI) and octadecyl isocyanate (ODI). **b** P6A was used as the host macrocycle of methylenediphenyl-4,4'-diisocyanate (MDI), dicyclohexylmethyl diisocyanate (DchDI), *m*-xylylene diisocyanate (XDI), and isophorone diisocyanate (IPDI). **c** Because 3-(triethoxysilyl)propyl Isocyanate (TESPI) and 3,5-bis(trifluoromethyl)phenyl isocyanate (BTMPI) were too large for the cavities of P5A and P6A, crystalline host–guest complexes were not obtained. X-ray crystal structure of the P6A–MDI complex in the *bc* plane (**d**, space-filling model) and *ac* plane (**e**, capped sticks model, the hydrogen atoms have been removed for clarity).

We also investigated the applicability to other isocyanate compounds. The tested isocyanate compounds are shown in Fig. 7a–c. As expected, hexyl isocyanate (HI), whose linear alkyl chain fits the 4.7 Å cavity of P5A, was successfully stabilised in water by the host–guest complexation with P5A (Supplementary Figs. 24 and 25). Interestingly, P5A also exhibited a protective effect against water vapour for linear alkyl isocyanate with longer chain, octadecyl isocyanate (ODI) (Supplementary Figs. 29 and 30). Previous studies have shown that P5A can encapsulate linear alkanes with chain lengths exceeding that of a single P5A cavity through inclusion by multiple P5A units, forming 1D channel structures^30^. In the case of ODI, the formation of a similar 1D channel structure was confirmed by PXRD analysis (Supplementary Fig. 31), suggesting that multiple P5A molecules encapsulate ODI and protect it from water vapour. Thus, we demonstrate that this supramolecular protection strategy is applicable to linear alkyl isocyanate compounds over a wide range of alkyl chain lengths. Methylenediphenyl-4,4'-diisocyanate (MDI) is also an important isocyanate molecule. However, the cavity size of P5A is not suitable for benzene rings. Therefore, we used pillar[6]arene (P6A) instead of P5A. Because P6A contains six benzene units, it has a larger cavity (6.7 Å) than that of P5A (4.7 Å), and its cavity matches benzene rings^35,36^. The crystalline host–guest complex of P6A and MDI was prepared by vapour diffusion of cyclohexane into a dichloromethane solution of P6A and an excess amount of MDI (Supplementary Fig. 33). X-ray crystallographic analysis revealed that the MDI molecule was encapsulated within the cavity of P6A in a 1:1 molar ratio, and the P6A units assembled into a 1D channel structure (Fig. 7d, e), similar to the P5A–HMDI crystal. The water protection ability was confirmed by ^1^H NMR and FT-IR measurements after the exposure of the P6A–MDI complex to water vapour (Supplementary Figs. 34 and 35). These results suggest that this strategy can be applied not only to linear aliphatic but also to aromatic isocyanate molecules by changing the cavity size of the host macrocycle. Similarly, dicyclohexylmethyl diisocyanate (DchDI), *m*-xylylene diisocyanate (XDI), and isophorone diisocyanate (IPDI) were protected from water by using P6A as the supramolecular protector (Supplementary Figs. 39, 40, 44, 45, 49, 50, 53 and 54).

However, P5A and P6A do not cover all isocyanate molecules. For instance, 3-(triethoxysilyl)propyl isocyanate (TESPI) and 3,5-bis(trifluoromethyl)phenyl isocyanate (BTMPI) cannot be protected using P5A or P6A (Fig. 7c). Owing to the bulky structures of TESPI and BTMPI with respect to the cavities of P5A and P6A, stable host–guest complexes were not obtained even in the crystalline state (Supplementary Figs. 55–57). This indicates that this strategy cannot be applied to isocyanates that cannot be guests for P5A or P6A. However, once we obtain host–guest complexes, the other isocyanate compounds can be protected from water. We believe that this can be accomplished by discovering pillar[*n*]arenes with suitable cavity sizes.

## Discussion

Host–guest complexation between pillar[*n*]arene macrocycles and water-sensitive isocyanate compounds in the crystalline state provides remarkable stability of the isocyanate groups against water owing to the hydrophobicity of crystalline pillar[*n*]arenes. The complex can be easily prepared on the gram scale by simply evaporating the solvent from a mixed solution of the two components. Notably, the stabilities of the supramolecularly protected isocyanates are comparable with those of conventional blocked isocyanates, which rely on covalent bonding for protection. X-ray crystallographic analysis provided direct evidence for the shielding of the isocyanate groups from water within the hydrophobic crystalline pillar[*n*]arenes. Furthermore, deprotection immediately occurred upon dissolution of the complex in the reaction solvent, allowing efficient polyurethane synthesis without the additional activation steps typically required for blocked isocyanates. This supramolecular approach overcomes the longstanding trade-off between strong protection and facile deprotection. Given the growing use of isocyanates not only as monomers for polyurethanes and polyureas but also as dynamic crosslinkers in network polymers^39,40^, stabilisation through host–guest encapsulation is expected to provide a broadly useful strategy in materials chemistry.

In addition to isocyanate compounds, we anticipate that this supramolecular protection strategy will be applicable to other water-sensitive species, such as acid chlorides, and organometallic compounds, including Grignard reagents and organolithium reagents. Among these compounds, organometallic compounds are particularly hazardous because they can spontaneously ignite upon exposure to atmospheric moisture. Therefore, strict handling under moisture-free and inert gas conditions, along with advanced technical expertise, is essential to avoid accidents, such as fires. If supramolecular protection can eliminate the risk of ignition, it would significantly improve the safety in synthetic chemistry. We believe that the supramolecular protection strategy demonstrated in this study represents an important step toward realising safe and reliable next-generation organic synthesis.

## Methods

### Materials

All commercially available reagents and solvents were used as received. Per-ethylated pillar[5]arene (P5A) and pillar[6]arene (P6A) were prepared according to the reported methods^41,42^. Deionised water was obtained from a Merck Elix-Essential-3 instrument with a Progard TS2 Pretreatment Pack.

### Nuclear magnetic resonance (NMR)

^1^H NMR spectra were recorded on a JEOL JNM-ECZ500R spectrometer. Chemical shifts were reported as the delta scale in ppm relative to the internal standards (δ = 7.26 ppm for ^1^H in CDCl_3_, δ = 5.32 ppm for ^1^H in dichloromethane-*d*_2_, δ = 2.50 ppm for ^1^H in DMSO-*d*_6_, and δ = 2.08 ppm for ^1^H in toluene-*d*_8_).

### Fourier transform infrared (FT-IR)

FT-IR measurements were performed on JASCO FT/IR-4X. Liquid samples were measured by the transmission method, and solid samples were measured using the KBr pellet method. Difference FT-IR measurements were also performed on JASCO FT/IR-4100 equipped with a mercury cadmium telluride (MCT) detector. Each few mg of samples was applied to a CaF_2_ plate and placed in an IR cell attached to a conventional closed gas circulation system. The samples were pretreated by evacuation at 298 K with a liquid nitrogen trap for 1 h. Contact angle values of the compounds were obtained by a Phoenix-Alpha P200A (Meiwafosis Co., Ltd., Tokyo, Japan). The compounds were coated on a glass substrate.

### Thermogravimetric analysis (TGA)

TGA curves were recorded with Hitachi High-Tech Science STA7200 at a heating rate of 10 °C/min under a flow of dry nitrogen.

### Powder X-ray diffraction (PXRD)

PXRD measurements were performed on a Rigaku Smart Lab high-resolution diffractometer.

### Size-exclusion chromatography (SEC)

SEC measurement was performed on SHIMADZU Nexera system. A series of TSKgel α−2500, α−3000, and α−4000 columns connected in tandem was used as the separation column.

### Vapour sorption experiments

Vapour adsorption and desorption isotherms were obtained by a BELSORP-max (BEL Japan Inc., Osaka, Japan) at 298 K.

### Contact angle measurements

Contact angle values of the compounds were obtained by a Phoenix-Alpha P200A (Meiwafosis Co., Ltd., Tokyo, Japan). The compounds were coated on a glass substrate.

### Preparation of the crystalline P5A–HMDI complex

Guest molecules, including solvent molecules, in the P5A crystals were removed according to the previously reported method^43^. P5A was dissolved in chloroform, and evaporation of the solvent afforded crystals of P5A. The crystals were dried under vacuum at 40 °C for 24 h. The resulting solid was redissolved in acetone, and evaporation of acetone afforded crystals of P5A. Drying the crystals at 80 °C under reduced pressure for 72 h removed the included acetone molecules to afford activated crystals of P5A. Removal of chloroform and acetone was confirmed by ^1^H NMR spectroscopy. P5A (1.00 g, 1.12 mmol) and HMDI (200 µL, 1.19 mmol) were dissolved in chloroform (20 mL) and placed in an open 50 mL vial. The solvent was evaporated within 30 min with a Smart Evaporator C1 (Biochromato) at 80 °C. The uncomplexed HMDI was removed by washing with cyclohexane, and the solid was collected by filtration, followed by drying in vacuum at room temperature. A white crystalline P5A–HMDI complex was obtained (1.15 g, 1.08 mmol, 96% yield based on P5A).

### Examination of the stability of the crystalline P5A–HMDI complex against water vapour

The crystalline P5A–HMDI complex (198 mg) was placed in an open 5 mL vial. The vial was then placed inside a 50 mL vial containing water (5.0 mL), tightly capped, and stored at room temperature to expose the sample to water vapour. After 40 days, the inner vial was removed, and the crystalline complex was dried under vacuum at 50 °C. The recovered crystals were subsequently analysed by ^1^H NMR and FT-IR spectroscopy to evaluate the extent of degradation of the isocyanate groups (Fig. 4d and Supplementary Fig. 8).

### Examination of the stability of the crystalline P5A–HMDI complex against water

The crystalline P5A–HMDI complex (100 mg) was added to a 20 mL vial containing water (5.0 mL), tightly capped, and stored at room temperature. After 40 days, the crystals were collected by filtration and then analysed by ^1^H NMR and FT-IR spectroscopy to evaluate the extent of degradation of the isocyanate groups (Fig. 4e and Supplementary Figs. 9 and 10).

### Examination of the stability of the crystalline P5A–HMDI complex against boiling water

The crystalline P5A–HMDI complex (30 mg) and 15 mL of water were placed in a 100 mL round-bottom flask. The mixture was refluxed at 100 °C for 1 h, after which the water was evaporated. The recovered crystals were then analysed by ^1^H NMR and FT-IR spectroscopy to evaluate the extent of degradation of the isocyanate groups (Fig. 4f and Supplementary Figs. 11 and 12).

### Examination of the stability of the crystalline P5A–HMDI complex against heat

The crystalline P5A–HMDI complex (10 mg) was placed in an open 10 mL vial. The vial was then placed inside a heater and stored at 160 °C for 1 h. The crystals were subsequently analysed by ^1^H NMR and FT-IR spectroscopy to evaluate the extent of degradation of the isocyanate groups (Fig. 4g and Supplementary Fig. 13).

## Supplementary information

Supplementary Information

Transparent Peer Review file

## Source data

Source data

## Data Availability

The data supporting the findings of this study, including synthetic procedures and spectroscopic characterisation data (NMR, PXRD, TGA and FT-IR), are provided in the Supplementary Information. Crystallographic data for the P5A–HMDI complex and P6A–MDI complex have been deposited with the Cambridge Crystallographic Data Centre (CCDC) under deposition numbers 2492825 and 2492826, respectively. All data are available from the corresponding author upon request. Source data are provided with this paper.
