# Supplementary material for: Supramolecular protection of isocyanates from water by encapsulation within hydrophobic crystalline pillar[n]arene macrocycles
Source: Nat Commun. 2026 May 9;17:6274. doi: 10.1038/s41467-026-72696-y (PMC13376218; doi:10.1038/s41467-026-72696-y)
Supplement: Supplementary file 1 — Supplementary Information [file 41467_2026_72696_MOESM1_ESM.pdf]

**Supplementary Information**

# Supramolecular Protection of Isocyanates from Water by Encapsulation within Hydrophobic Crystalline Pillar[*n*]arene Macrocycles

Kiichi Yasuzawa,[a] Kouhei Sutou,[a] Katsuto Onishi,[a] Shunsuke Ohtani,[a] Kenichi Kato,[a] Hiroshi Yamazaki, [b] Junko N. Kondo, [c,d] Shigehisa Akine, [e,f] Tomoki Ogoshi*[a,f]

[a] Mr. K. Yasuzawa, Mr. K. Sutou, Mr. K. Onishi, Dr. S. Ohtani, Dr. K. Kato, Prof. Dr. T. Ogoshi

Department of Synthetic Chemistry and Biological Chemistry, Graduate School of Engineering, Kyoto University

Katsura, Nishikyo-ku, Kyoto, 615-8510, Japan

E-mail: ogoshi.tomoki.3s@kyoto-u.ac.jp

[b] Dr. H. Yamazaki

School of Materials and Chemical Technology, Tokyo Institute of Technology, 4259 Nagatsuta, Midori-ku, Yokohama 226-8503, Japan

[c] Prof. Dr. J. N. Kondo

Chemical Resources Laboratory, Tokyo Institute of Technology, 4259 Nagatsuta, Midori-ku,

Yokohama 226-8503, Japan

[d] Prof. Dr. J. N. Kondo

Professor for Institute Management, Office of Communication and DEI, Institute of Science Tokyo, 4259 Nagatsuta, Midori-ku, Yokohama 226-8503, Japan

[e] Prof. Dr. S. Akine

Graduate School of Natural Science and Technology, Kanazawa University

Kakuma-machi, Kanazawa, 920-1192, Japan

[f] Prof. Dr. S. Akine, Prof. Dr. T. Ogoshi

WPI Nano Life Science Institute, Kanazawa University

Kakuma-machi, Kanazawa, 920-1192, Japan

**Contents**

1. Preparation of Crystalline P5A–HMDI Complex
2. Hydrophobicity of Crystalline P5A
3. Stability Investigation of HMDI and Crystalline P5A–HMDI Complex
4. Deprotection and Polymerization of Crystalline P5A–HMDI Complex
5. Preparation and Stability Investigation of the Other Isocyanates
6. X-ray Crystallographic Analysis
7. References

## 1. Preparation of Crystalline P5A–HMDI Complex

Preparation of the crystalline P5A–HMDI complex (evaporation method)

P5A (1.00 g, 1.12 mmol) and hexamethylene diisocyanate (HMDI) (200 µL, 1.19 mmol) were dissolved in chloroform (20 mL) and placed in an open 50 mL vial. The solvent was evaporated within 30 min with Smart Evaporator C1 (Biochromato) at 80 ºC. Uncomplexed HMDI was washed with cyclohexane and the solid was collected by filtration, followed by drying in vacuum at room temperature. A white crystalline P5A–HMDI complex was obtained (1.15 g, 1.08 mmol, 96% yield based on P5A).

$^{1}$H NMR spectroscopy for prepared P5A–HMDI complex

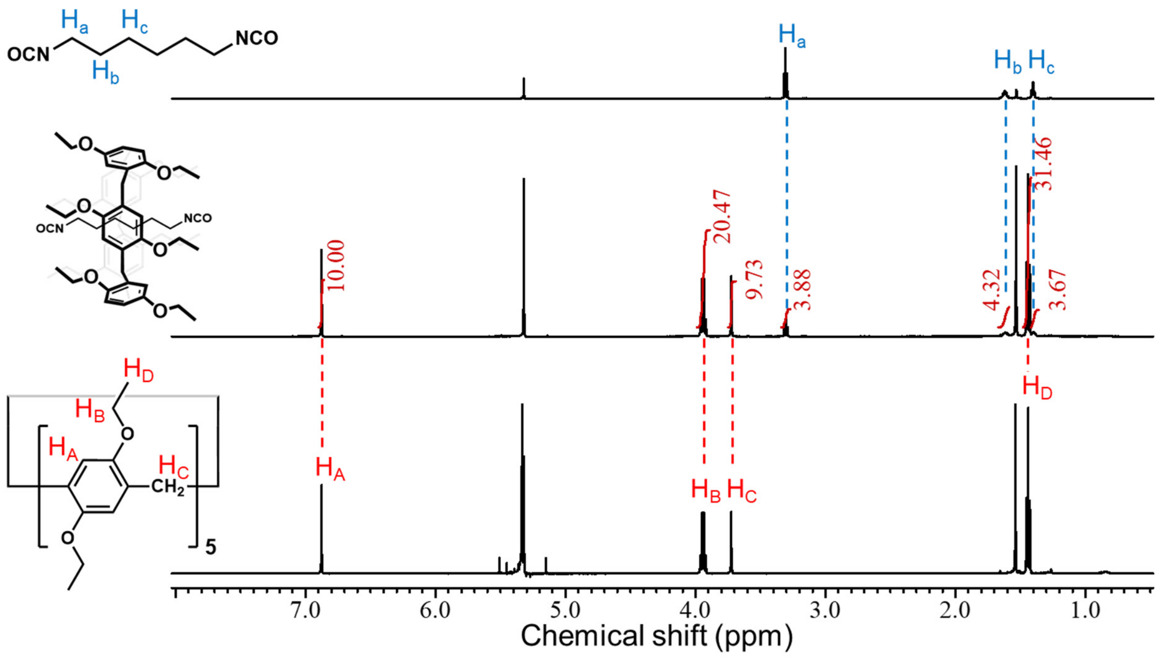


**Supplementary Fig. 1** $^{1}$H NMR spectra ($CD_2Cl_2$, 298 K) of HMDI (top), P5A–HMDI complex prepared by evaporation method (middle), and P5A (bottom), full range 1–7 ppm with integral traces.

PXRD measurements

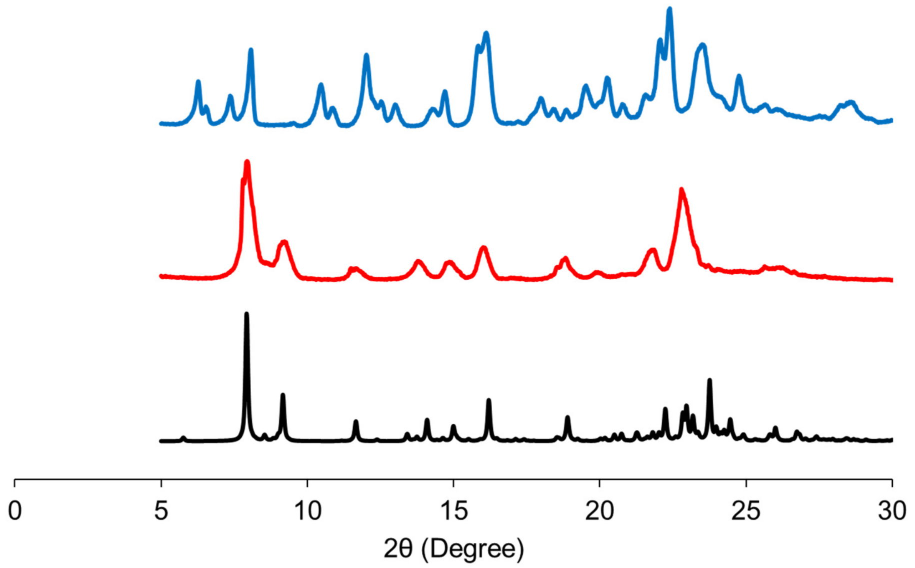


**Supplementary Fig. 2** PXRD patterns of individual P5A (blue, top), crystalline P5A–HMDI complex prepared by evaporation method (red, middle), and simulated pattern calculated from its single crystal structure shown in Figs. 3i and j (black, bottom).

PXRD profiles of the prepared P5A–HMDI complex showed clear crystallinity. The patterns corresponding to the individual P5A disappeared, and a new set of peaks appeared, indicating the formation of crystalline host–guest complex. Furthermore, the PXRD pattern was also consistent with the simulated pattern calculated from its single crystal structure, confirming that the complex adopts a 1D channel structure, as shown in Figs. 3i and j.

## 2. Hydrophobicity of Crystalline P5A

Preparation of activated P5A crystals

P5A crystals were activated according to a previously reported procedure.[S1] P5A was dissolved in acetone, and the evaporation of the solvent afforded P5A crystals. Drying at 80 °C under reduced pressure for 24 h completely removed the acetone molecules, yielding activated P5A crystals.

FT-IR spectroscopy for activated P5A crystals

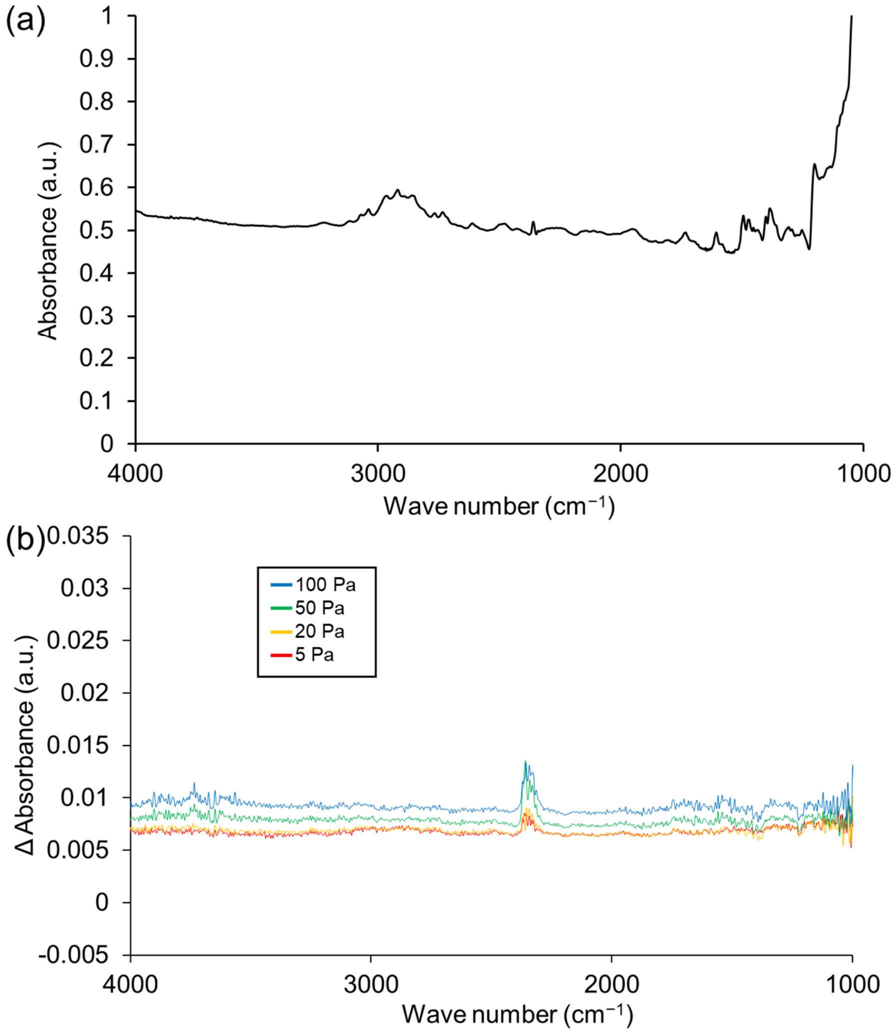


**Supplementary Fig. 3** (a) FT-IR spectrum of activated P5A crystals measured under vacuum (298 K). (b) Difference FT-IR spectra obtained by subtracting IR spectrum of activated P5A crystals measured under vacuum from IR spectra of P5A crystals measured under exposure to water vapor at 5, 20, 50, and 100 Pa (298 K).

Adsorption–desorption analysis of activated P5A crystals

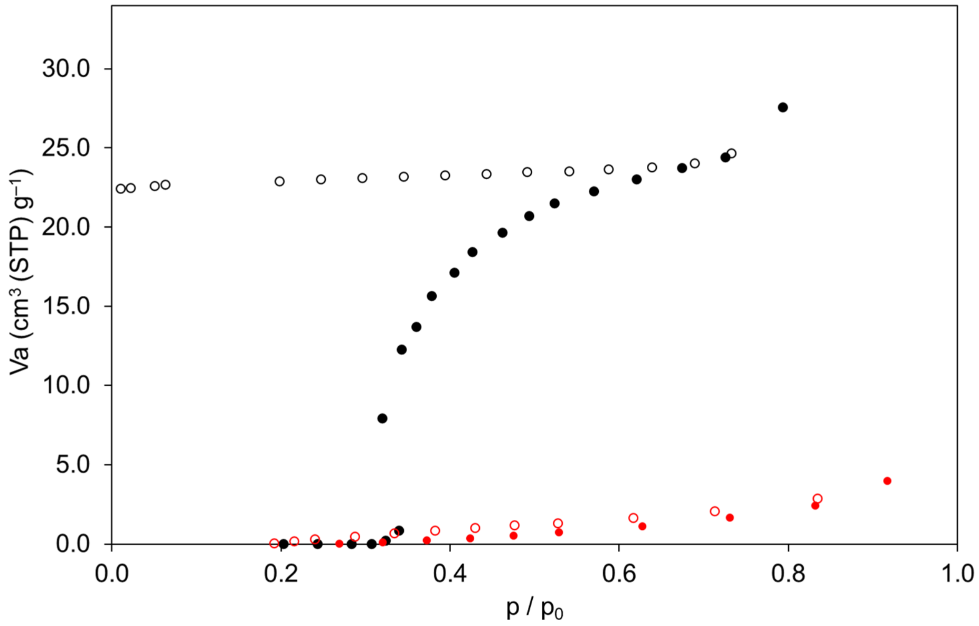


**Supplementary Fig. 4** Water (red circles) and *n*-hexane (black circles) vapor sorption isotherms of activated P5A crystals. Solid symbols: adsorption; open symbols: desorption.

Adsorption isotherms of activated P5A crystals took up *n*-hexane vapor in a 1:1 molar ratio, whereas no adsorption of water was observed. The slight increase near the saturated water vapor pressure is attributed to surface condensation during measurement.

Difference FT-IR spectroscopy and adsorption–desorption analysis clearly demonstrates that P5A crystals possess very high hydrophobicity.

<u>Water contact angle measurement of P5A film</u>

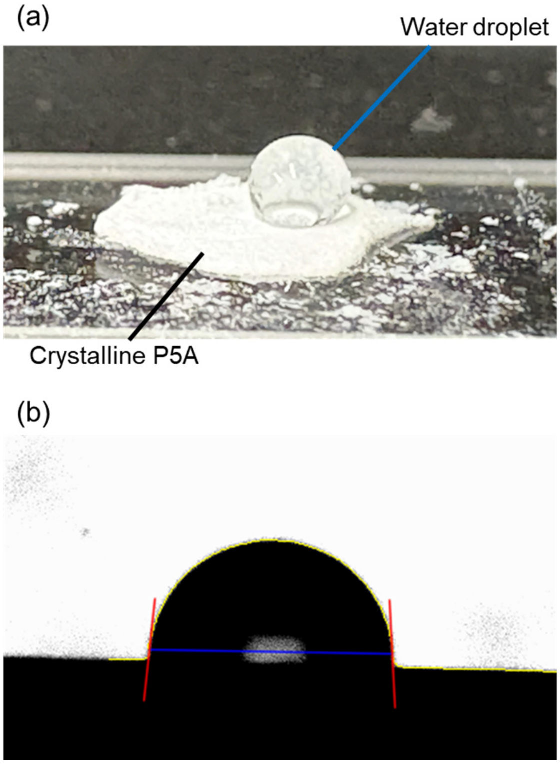


**Supplementary Fig. 5** (a) Photograph of crystalline P5A after dropping water. (b) Photograph of the water contact angle of a P5A film. P5A films were prepared by spin-coating a 4 mM chloroform solution of P5A.

The measured angles were in the range of 81 ± 5°, indicating that P5A possesses high hydrophobicity toward liquid water.

## 3. Stability Investigation of HMDI and Crystalline P5A–HMDI Complex

Examination of the stability of HMDI against water vapor

HMDI (30.0 μL) was placed in an open 5 mL vial. This vial was then placed inside a 50 mL vial containing water (5.0 mL). The outer vial was tightly capped and stored at room temperature to expose the sample to water vapor. After 3 days, the inner vial was removed and the compounds were dried under vacuum at room temperature. The obtained product was hardly soluble in a range of solvents, including water, DMSO, DMF, MeOH, acetone, THF, dichloromethane, chloroform, and *n*-hexane.

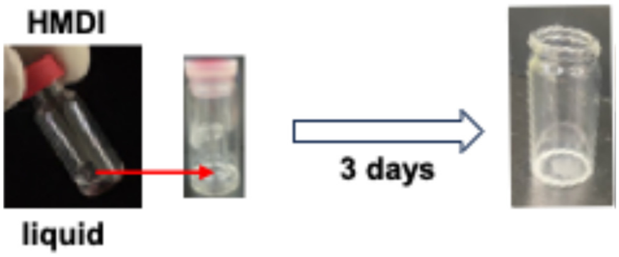


**Supplementary Fig. 6** Exposure of liquid HMDI to water vapour. Liquid HMDI produced insoluble polyurea.

Examination of the stability of crystalline P5A–HMDI complex against water vapor

Crystalline P5A–HMDI complex (198 mg) was placed in an open 5 mL vial. This vial was then placed inside a 50 mL vial containing water (5.0 mL). The outer vial was tightly capped and stored at room temperature to expose the sample to water vapor. After 40 days, the inner vial was removed and the crystalline complex was dried under vacuum at 50 °C.

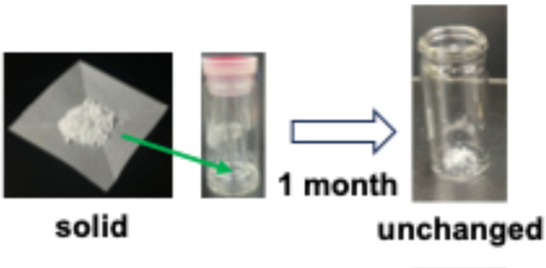


**Supplementary Fig. 7** Exposure of the crystalline P5A–HMDI complex to water vapour. While liquid HMDI produced insoluble polyurea, the crystalline P5A–HMDI complex showed no observable changes.

$^{1}$H NMR spectroscopy for P5A–HMDI complex upon water vapor exposure

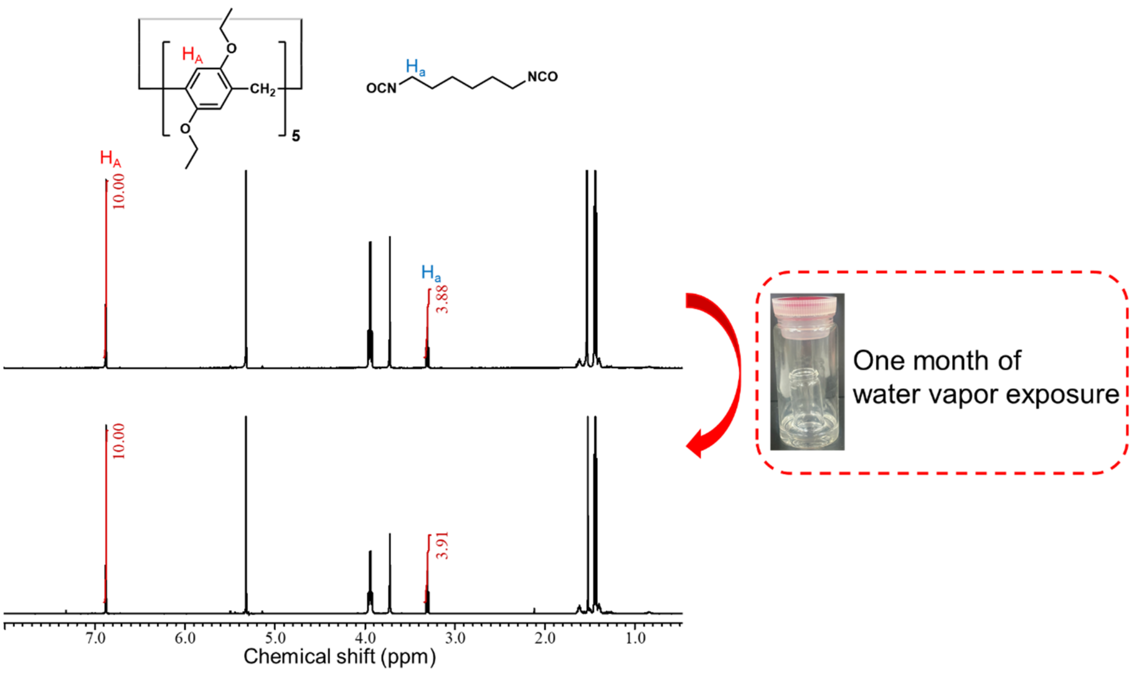


**Supplementary Fig. 8** $^{1}$H NMR spectra ($CD_2Cl_2$, 298 K) of the P5A–HMDI complex prepared by evaporation method (top) and after one month of water vapor exposure (bottom), (insert: photo of the setup for water vapor exposure), full range 1–7 ppm with integral traces.

Examination of the stability of crystalline P5A–HMDI complex against water

Crystalline P5A–HMDI complex (100 mg) was added into a 20 mL vial containing water (5.0 mL), tightly capped, and stored at room temperature. After 40 days, the crystals were collected by filtration.

$^1$H NMR spectroscopy for P5A–HMDI complex upon immersion in water

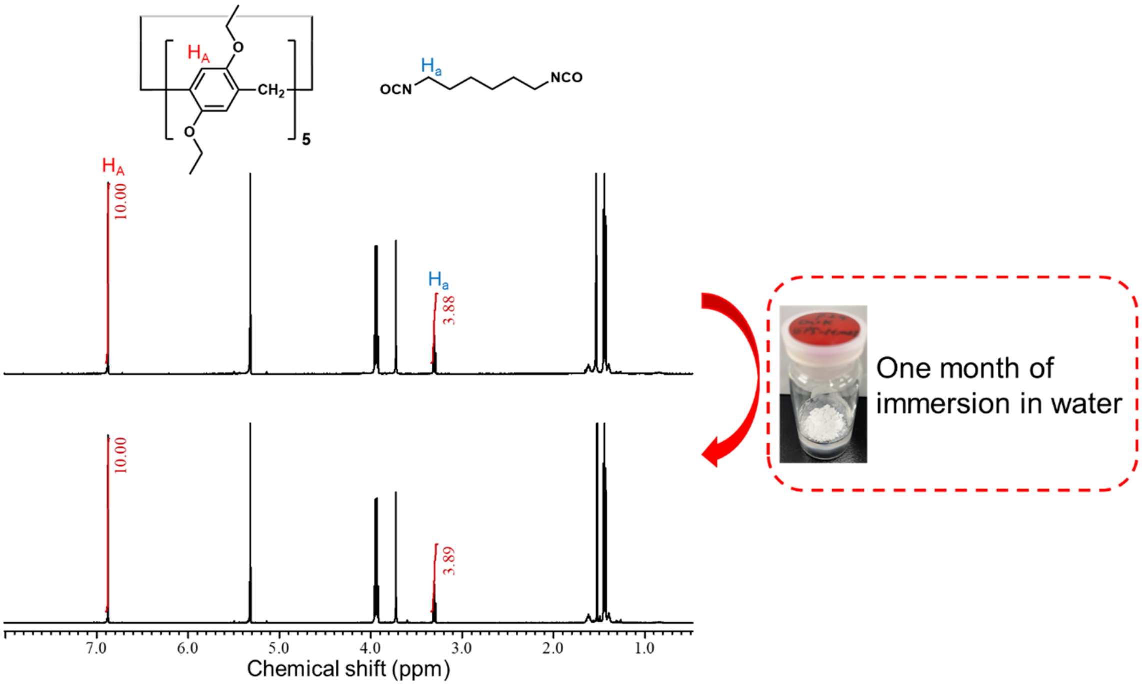


**Supplementary Fig. 9** $^1$H NMR spectra ($CD_2Cl_2$, 298 K) of the P5A–HMDI complex prepared by evaporation method (top) and after one month of immersion in water (bottom), (insert: photo of the setup for immersion in water), full range 1–7 ppm with integral traces.

FT-IR spectroscopy for P5A–HMDI complex upon immersion in water

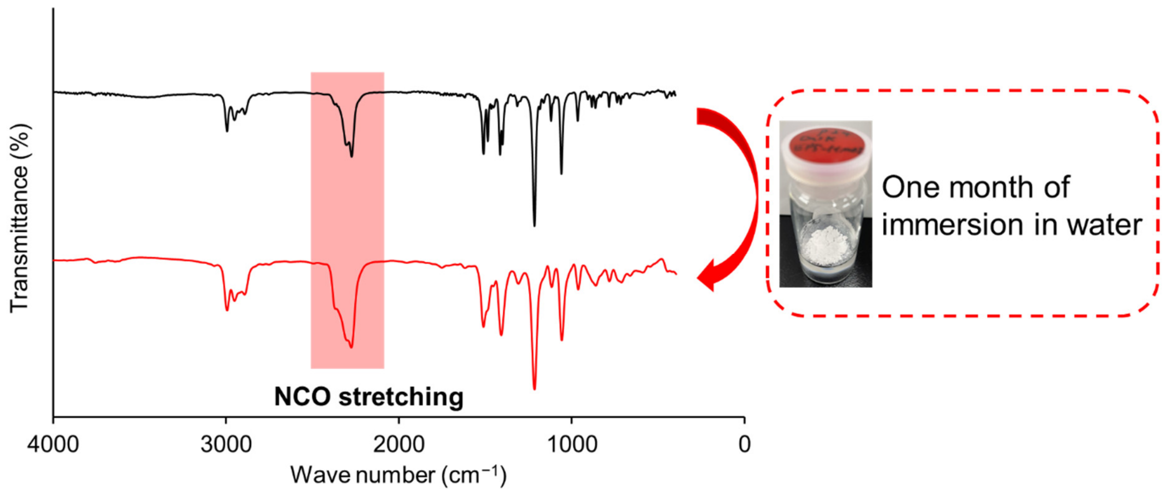


**Supplementary Fig. 10** FT-IR spectra of the P5A–HMDI complex prepared by evaporation method (top, black) and after one month of immersion in water (bottom, red), (insert: photo of the setup for immersion in water).

Examination of the stability of crystalline P5A–HMDI complex against boiling water

Crystalline P5A–HMDI complex (30 mg) and 15 mL of water were placed in a 100 mL round-bottom flask. The mixture was refluxed at 100 °C for 1 h, after which the water was evaporated.

$^1$H NMR spectroscopy for P5A–HMDI complex upon immersion in boiling water

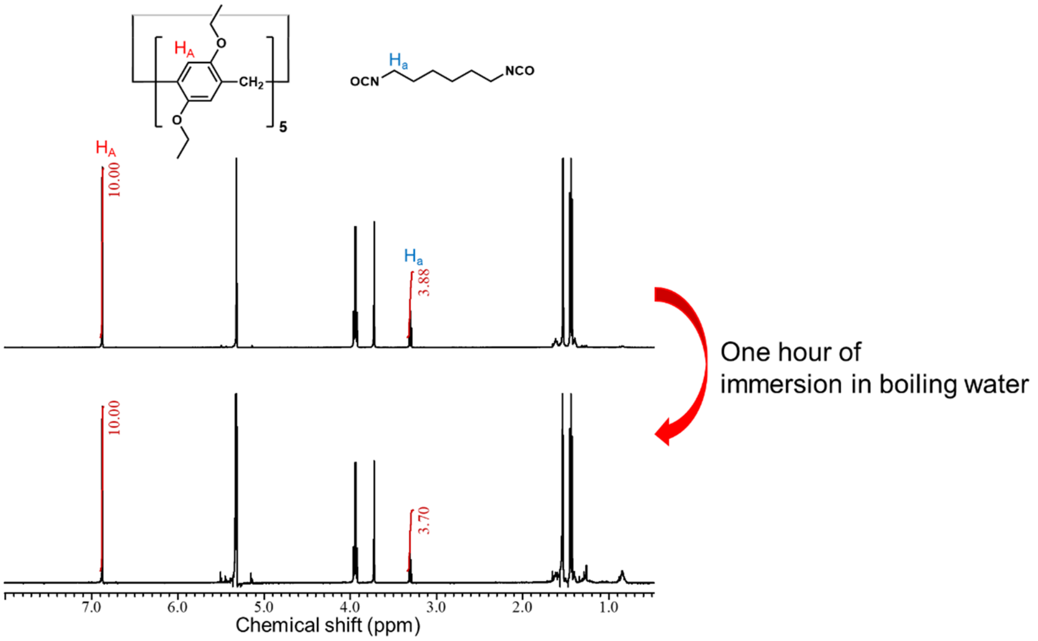


**Supplementary Fig. 11** $^1$H NMR spectra ($CD_2Cl_2$, 298 K) of the P5A–HMDI complex prepared by evaporation method (top) and after one hour of immersion in boiling water (bottom), full range 1–7 ppm with integral traces.

The degradation ratio was calculated from the integration ratio of a HMDI proton ($H_a$) to an aromatic proton of P5A ($H_A$) (from 3.88 to 3.70).

Degradation ratio (%) = 3.70 / 3.88 × 100 = 95%

FT-IR spectroscopy for P5A–HMDI complex upon immersion in boiling water

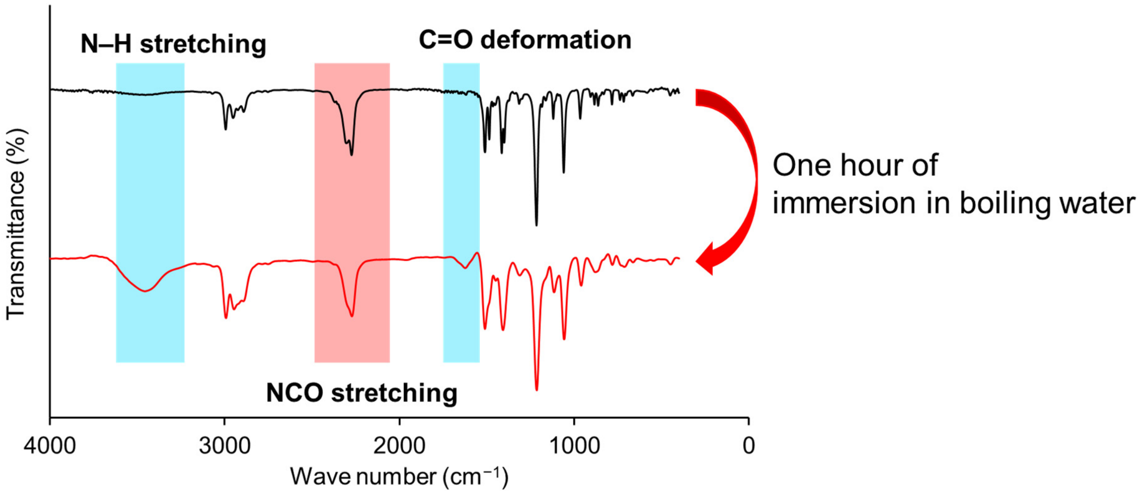


**Supplementary Fig. 12** FT-IR spectra of the P5A–HMDI complex prepared by evaporation method (top, black) and after one hour of immersion in boiling water (bottom, red).

The appearances of C=O deformation (1600 $cm^{-1}$) and N–H stretching (3300 $cm^{-1}$) signals suggest that perfect protection was not achieved; however, the majority of the isocyanate groups remained protected even after immersion in boiling water.

Examination of the stability of crystalline P5A–HMDI complex against heat

Crystalline P5A–HMDI complex (10 mg) was placed in an open 10 mL vial. This vial was then placed inside a heater and stored at 160 °C for 1 h.

$^{1}$H NMR spectroscopy for P5A–HMDI complex upon heating

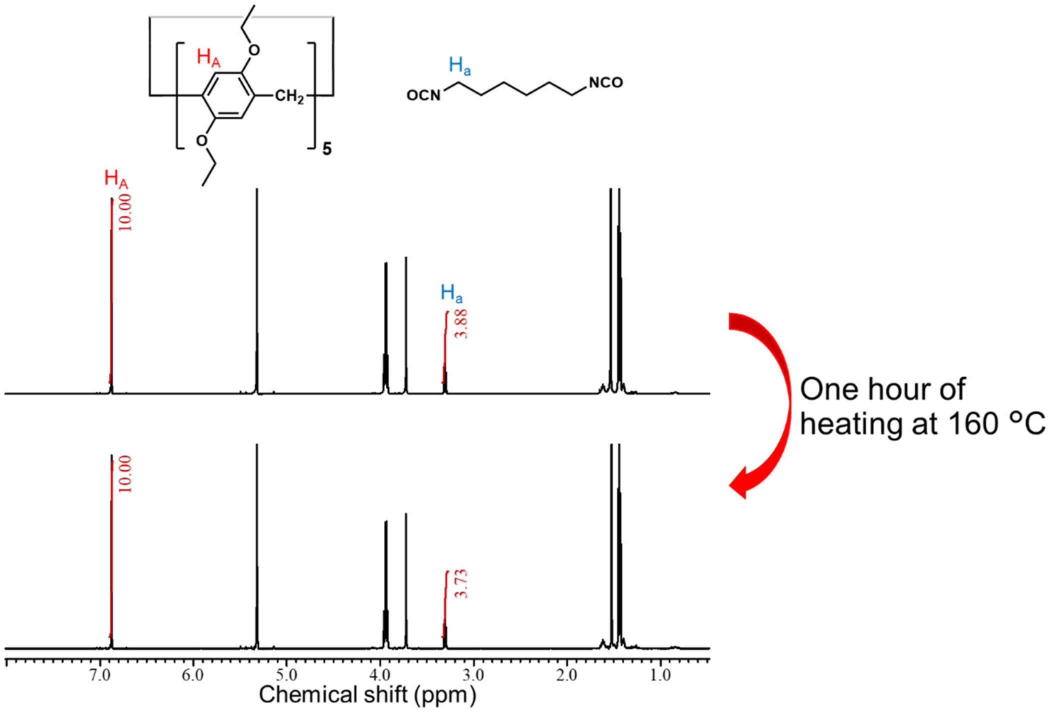


**Supplementary Fig. 13** $^{1}$H NMR spectra ($CD_2Cl_2$, 298 K) of the P5A–HMDI complex prepared by evaporation method (top) and after one hour of heating at 160 °C (bottom), full range 1–7 ppm with integral traces.

Synthesis of blocked HMDI

Blocked HMDI was prepared according to the reported method.[S2] A 50 mL round-bottom flask was charged with HMDI (1.00 mL, 6.24 mmol) and ε-caprolactam (2.24 g, 19.8 mmol, 3.2 eq.). The mixture was stirred under a nitrogen atmosphere at 90 °C for 18 h. After the reaction, the mixture was dissolved in a small amount of acetone, and then reprecipitated in excess water. The solution was filtrated to afford white solid blocked HMDI (0.795 g, 2.02 mmol, 32% yields based on HMDI).

TGA measurements

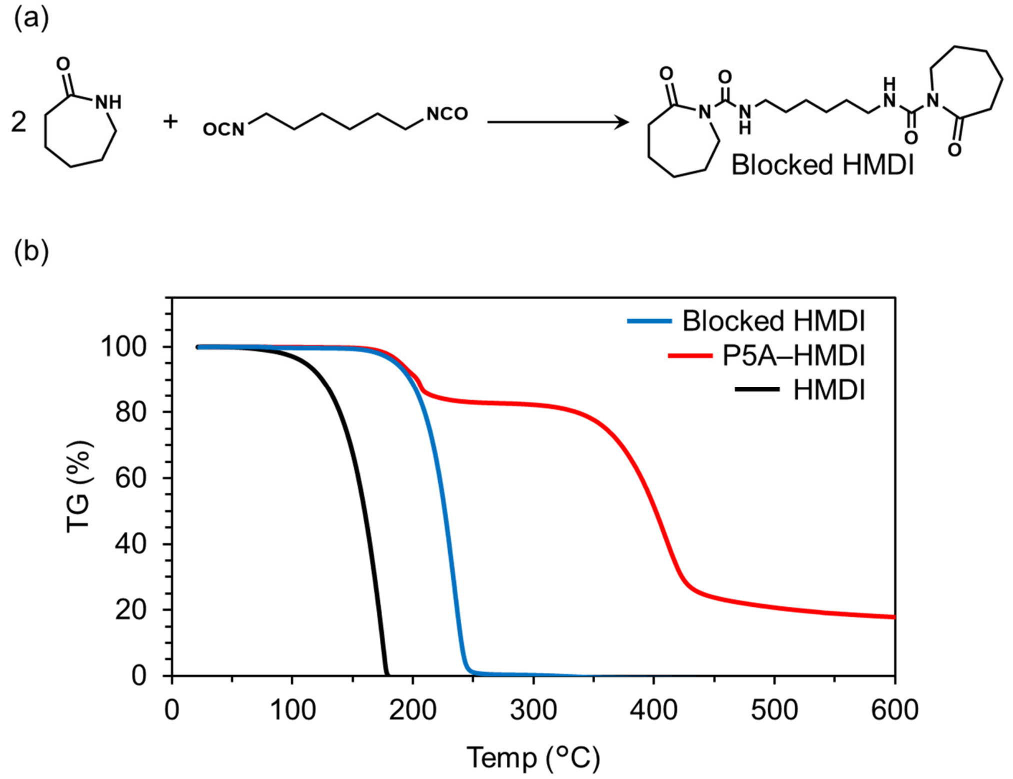


**Supplementary Fig. 14** (a) Synthesis of blocked HMDI. (b) TG curves of blocked HMDI (blue), P5A–HMDI complex (red), and neat HMDI (black).

The theoretical mass ratio of HMDI to P5A–HMDI complex, calculated from their molecular weights (HMDI: 168.2; P5A: 891.2), is 15.9% [168.2 / (168.2 + 891.2) = 15.9]. The TGA measurement of P5A–HMDI complex showed a weight loss of 16.2% at 230 °C, which is in good agreement with the theoretical value, confirming that P5A–HMDI complex is a 1:1 host–guest complex.

## 4. Deprotection and Polymerization of Crystalline P5A–HMDI Complex

$^1$H NMR spectroscopy in $CDCl_3$

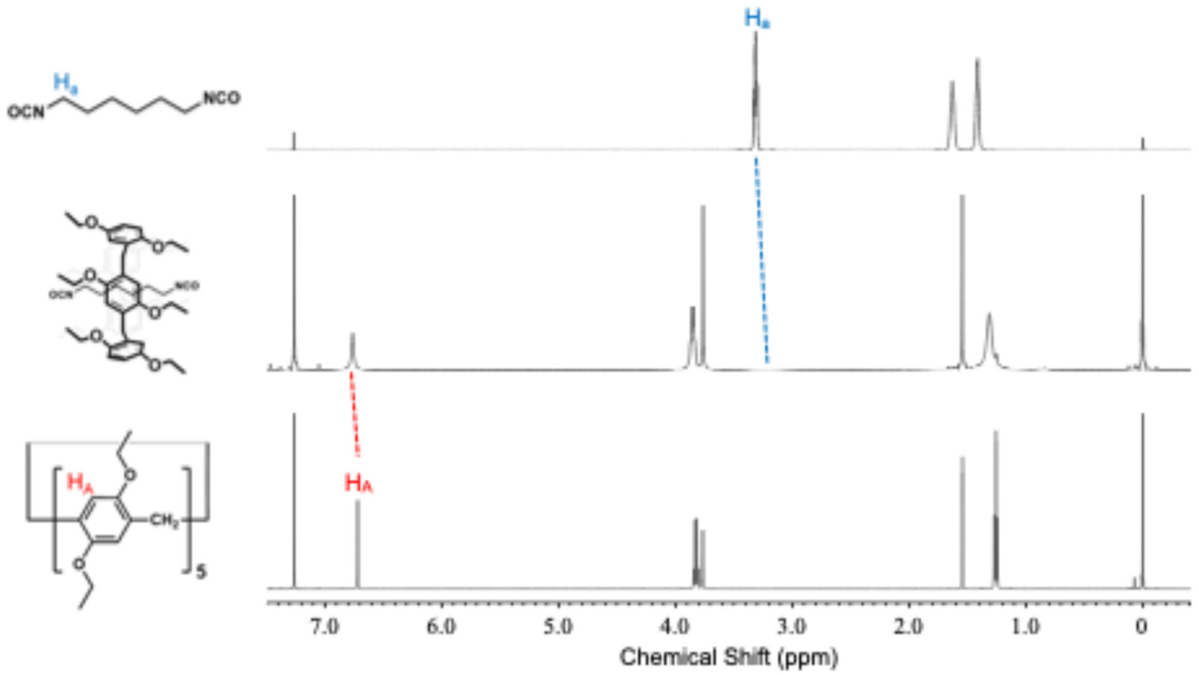


**Supplementary Fig. 15** $^1$H NMR spectra ($CDCl_3$, 298 K) of HMDI (top), P5A–HMDI complex prepared by evaporation method (middle), and P5A (bottom).

**Supplementary Note 1** Although peak shifts and broadening were observed in solution, host–guest complexation is an equilibrium process, unlike in the crystalline state where only the inclusion complex exists, indicating that sufficient dissociation occurred in solution.

$^1$H NMR spectroscopy in the condition of polyurethane synthesis

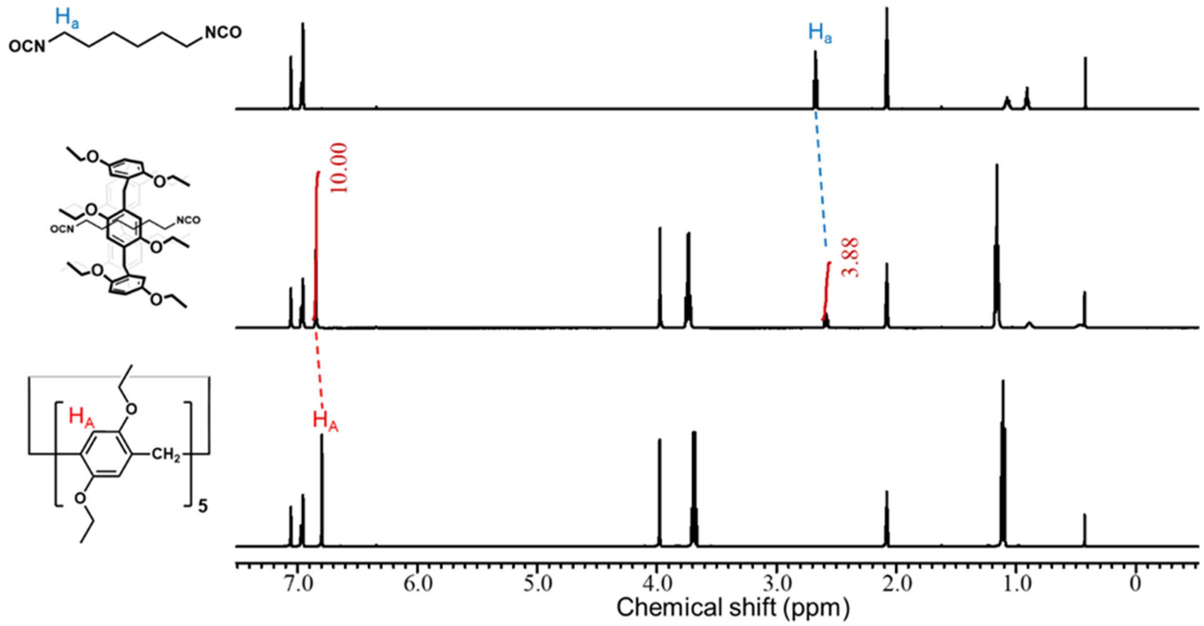


**Supplementary Fig. 16** $^1$H NMR spectra (toluene-$d_8$, 363 K, 10 mM) of HMDI (top), P5A–HMDI complex prepared by evaporation method (middle), and P5A (bottom).

**Supplementary Note 2** Although slight peak shifts are still observed, indicating a small amount of the complex remains, the dissociation would proceed sufficiently for polymerization.

Polyurethane synthesis from P5A–HMDI complex and triethylene glycol[S3]

A 50 mL round-bottom flask was charged with triethylene glycol (37.0 μL, 277 μmol), P5A–HMDI complex (300 mg, corresponding to 1.0 eq. of HMDI), DBTDL (0.333 μL, 0.002 eq.), and toluene (2 mL) as the reaction solvent. The mixture was stirred under a nitrogen atmosphere at 90 °C for 3 h. After the reaction, the supernatant solution was decanted to afford the precipitated solid corresponding to the desired polyurethane. The solid was washed with toluene and dried under vacuum at 50 °C to give the polyurethane in 41% yield.

$^1$H NMR spectroscopy for synthesized polyurethane

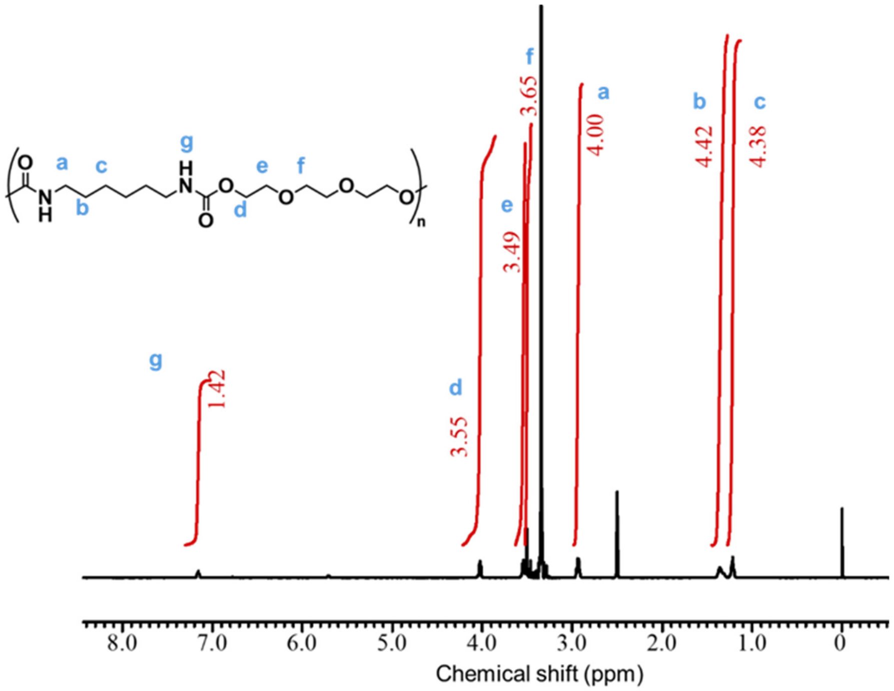


**Supplementary Fig. 17** $^1$H NMR spectrum (DMSO-$d_6$, 298 K) of the polyurethane synthesized from P5A–HMDI complex and triethylene glycol.

FT-IR spectroscopy for synthesized polyurethane

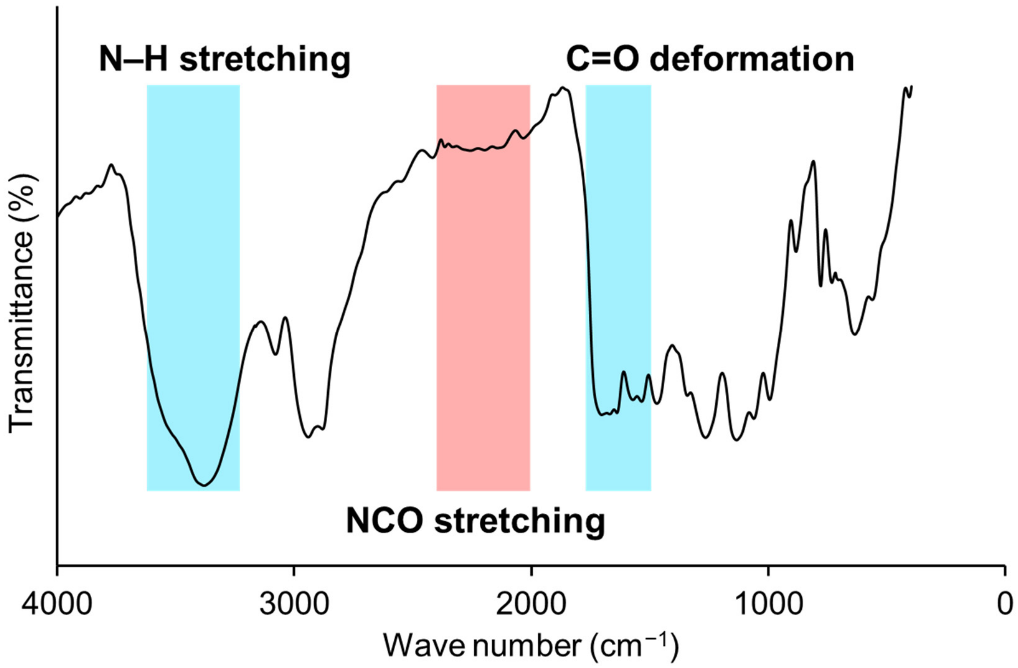


**Supplementary Fig. 18** FT-IR spectrum of the polyurethane synthesized from P5A–HMDI complex and triethylene glycol.

The disappearance of the NCO peaks together with the appearance of the urethane peak in the FT-IR spectrum also confirmed the successful synthesis of the target polyurethane.

SEC measurement for synthesized polyurethane

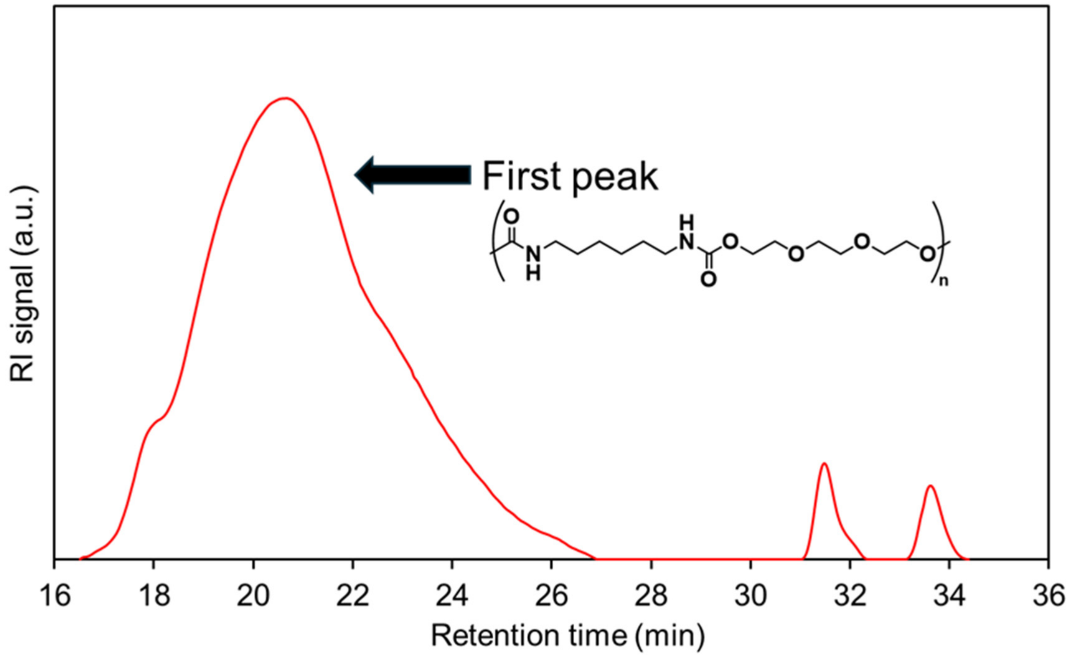


**Supplementary Fig. 19** SEC traces (eluent: DMF) of the polyurethane synthesized from P5A–HMDI complex and triethylene glycol. The first peak corresponds to the target polyurethane ($M_n = 3.2 \times 10^4$, $M_w = 1.0 \times 10^5$, $M_w/M_n = 3.3$).

Collection of P5A after polymerization

After polyurethane synthesis, the supernatant solution and the toluene used for washing were evaporated. The residue was redissolved in dichloromethane and passed through a membrane filter to remove insoluble impurities, and then the filtrate was concentrated. The residue was redissolved in 2 mL of dichloromethane and reprecipitated by addition to 40 mL of cold methanol. After centrifugation, the solution was decanted and the residue was dried under vacuum to afford white solid P5A (214.9 mg, 85% recovery).

$^1$H NMR spectroscopy for collected P5A

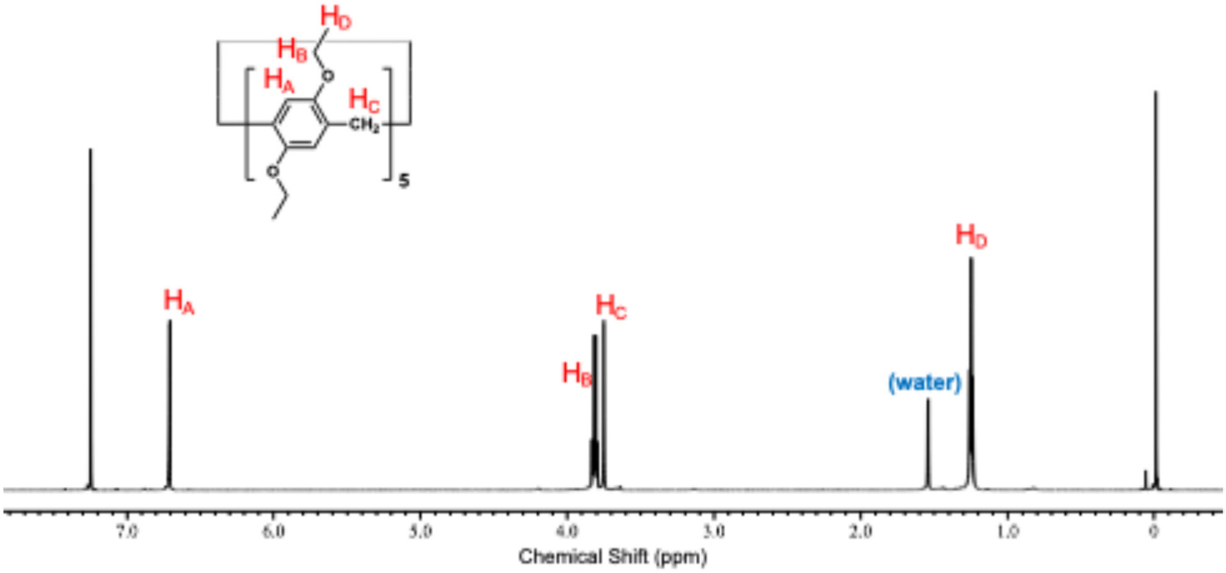


**Supplementary Fig. 20** $^1$H NMR spectra ($CDCl_3$, 298 K) of collected P5A after polymerization of P5A–HMDI complex and triethylene glycol.

No peaks attributable to impurities were observed, confirming that pure P5A was successfully recovered.

## 5. Preparation and Stability Investigation of the Other Isocyanates

Examination of the stability of HI against water vapor

Hexyl isocyanate (HI) (3.0 mg) was placed in an open 5 mL vial. This vial was then placed inside a 50 mL vial containing water (5.0 mL). The outer vial was tightly capped and stored at room temperature to expose the sample to water vapor. After 3 days, the inner vial was removed and the compounds were dried under vacuum at room temperature. The exposed sample was subsequently analyzed by $^{1}H$ NMR and FT-IR spectroscopy to evaluate the extent of degradation of the isocyanate groups.

Stability investigation of HI

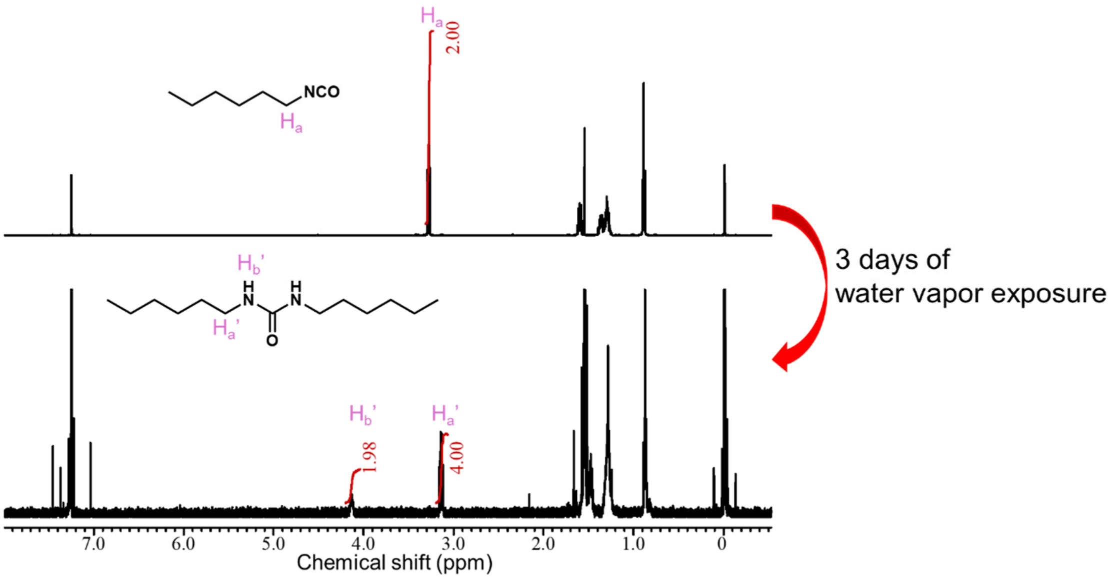


**Supplementary Fig. 21** $^{1}H$ NMR spectra ($CDCl_3$, 298 K) of HI (top) and after 3 days of water vapor exposure (bottom).

$^{1}H$ NMR analysis revealed that HI decomposed to the corresponding amine, which subsequently underwent further reactions to form a urea dimer.

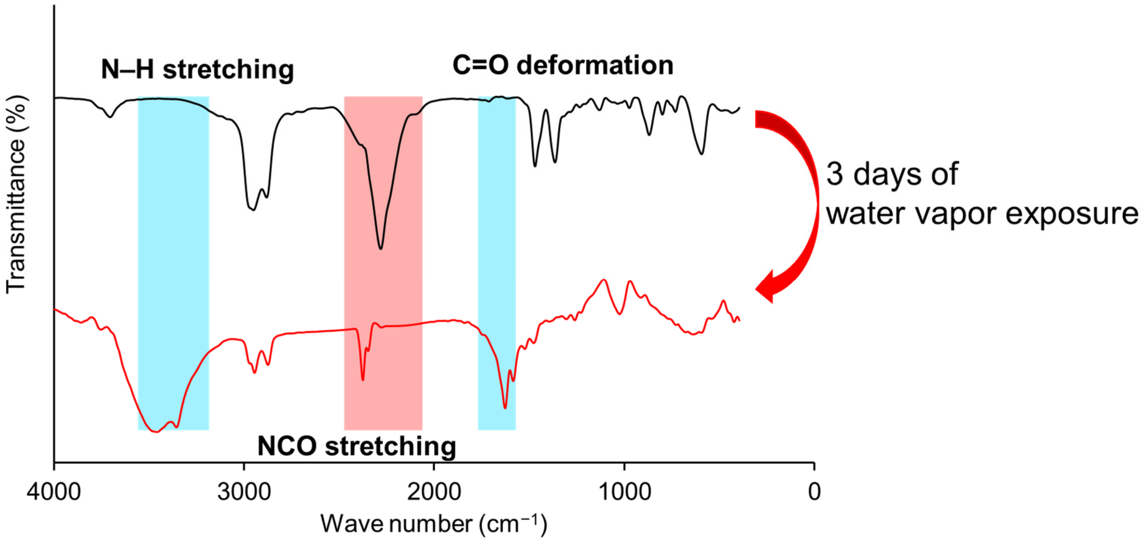


**Supplementary Fig. 22** FT-IR spectra of the HI (top, black) and after 3 days of water vapor exposure (bottom, red).

Preparation of crystalline P5A–HI complex (immersion method)

P5A (50.0 mg) was immersed in excess HI (500 μL) and stirred for 12 h at 40 °C in 5 mL vial. Uncomplexed HI was washed with cyclohexane and the solid was collected by filtration, followed by drying under vacuum at room temperature. A white crystalline P5A–HI complex was obtained.

$^{1}H$ NMR spectroscopy for prepared P5A–HI complex

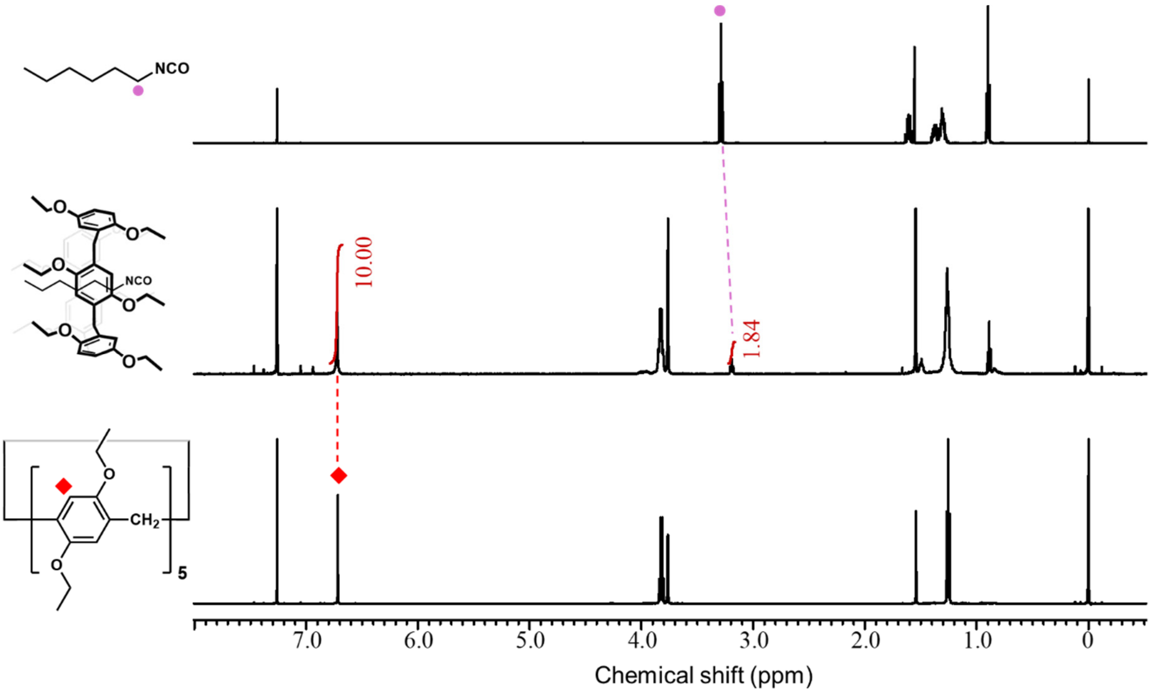


**Supplementary Fig. 23** $^{1}H$ NMR spectra ($CDCl_3$, 298 K) of HI (top), prepared P5A–HI complex (middle), and P5A (bottom).

1:1 complexation was confirmed based on the integrals of an aromatic proton on P5A (red square, 10H) and ethylene protons of HI (purple circle, 2H).

Examination of the stability of crystalline P5A–HI complex against water vapor

Crystalline P5A–HI complex (3.3 mg) was placed in an open 5 mL vial. This vial was then placed inside a 50 mL vial containing water (5.0 mL). The outer vial was tightly capped and stored at room temperature to expose the sample to water vapor. After 3 days, the inner vial was removed and the crystalline complex was dried under vacuum at room temperature.

Stability investigation of P5A–HI complex

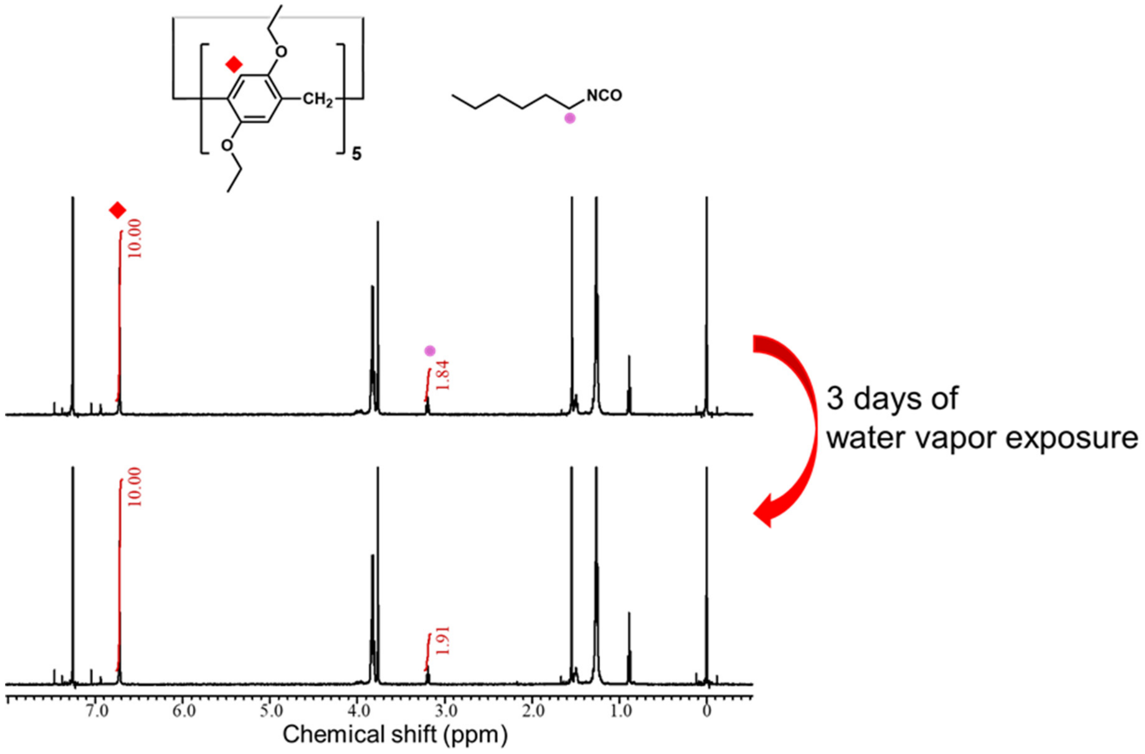


**Supplementary Fig. 24** $^{1}$H NMR spectra ($CDCl_3$, 298 K) of the P5A–HI complex prepared by immersion method (top) and after 3 days of water vapor exposure (bottom).

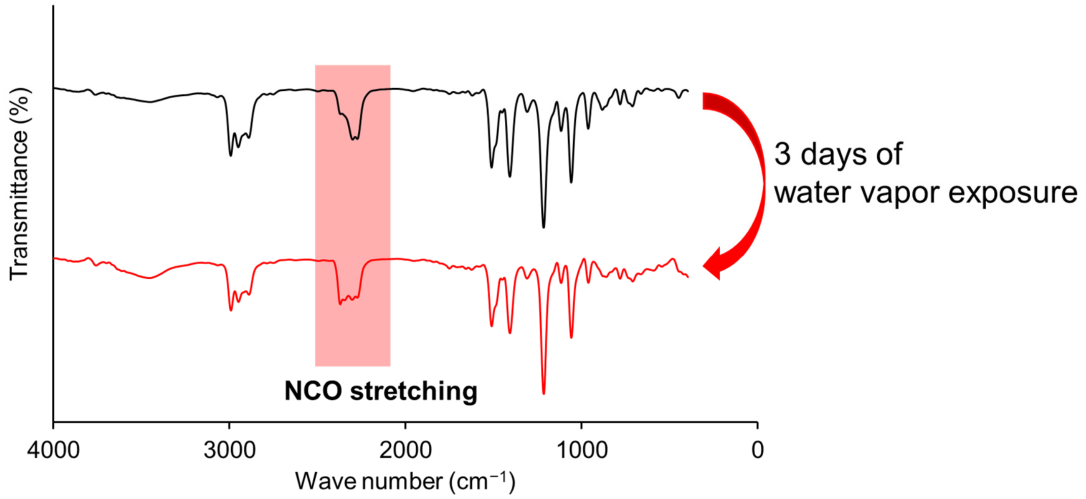


**Supplementary Fig. 25** FT-IR spectra of the P5A–HI complex prepared by immersion method (top, black) and after 3 days of water vapor exposure (bottom, red).

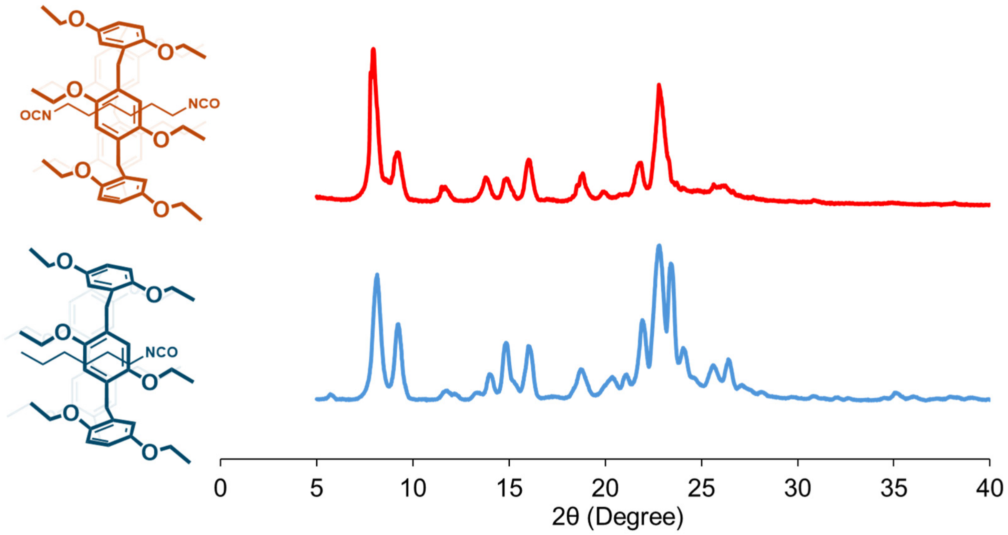


**Supplementary Fig. 26** PXRD patterns of the crystalline P5A–HMDI complex prepared by evaporation method (red, top) and the crystalline P5A–HI complex prepared by immersion method (blue, bottom).

The PXRD pattern of P5A–HI complex was similar to that of P5A–HMDI, suggesting that the crystalline P5A–HI complex also adopts a 1D channel structure.

Examination of the stability of ODI against water vapor

Octadecyl isocyanate (ODI) (11.4 mg) was placed in an open 5 mL vial. This vial was then placed inside a 50 mL vial containing water (5.0 mL). The outer vial was tightly capped and stored at room temperature to expose the sample to water vapor. After 3 days, the inner vial was removed and the compounds were dried under vacuum at room temperature. The exposed sample was subsequently analyzed by FT-IR spectroscopy to evaluate the extent of degradation of the isocyanate groups. (Because the exposed sample showed poor solubility, quantitative analysis by $^{1}H$ NMR spectroscopy was difficult.)

Stability investigation of ODI

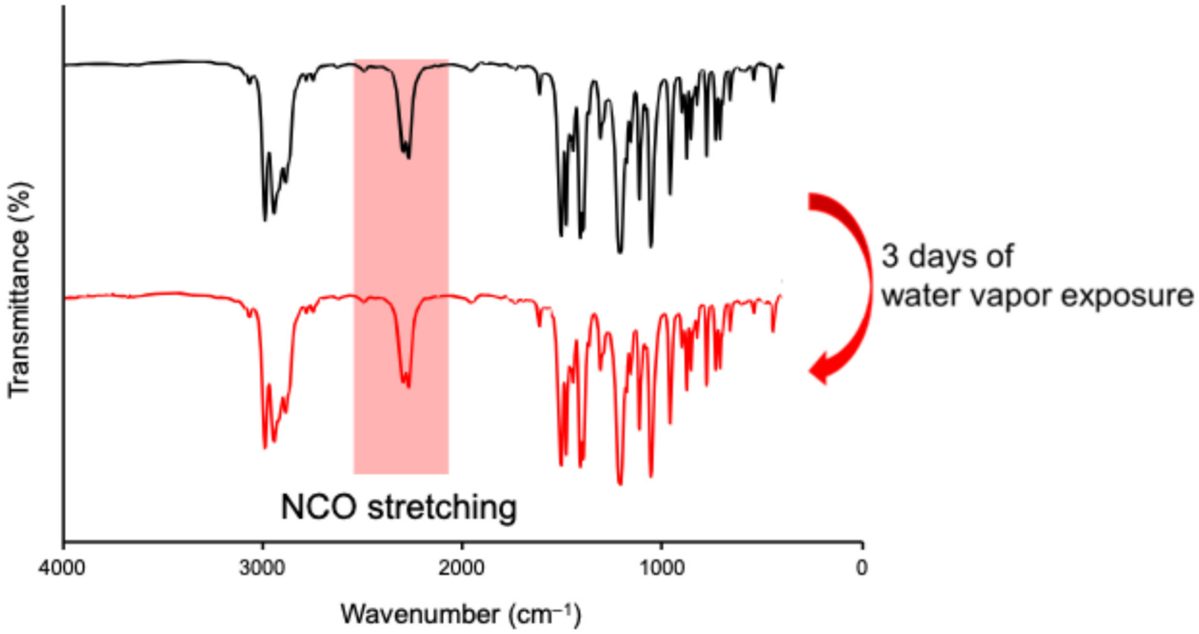


**Supplementary Fig. 27** FT-IR spectra of the ODI (top, black) and after 3 days of water vapor exposure (bottom, red).

In addition to the peaks attributed to the isocyanate groups, peaks assignable to urea groups formed after degradation were also observed.

Preparation of crystalline P5A–ODI complex (evaporation method)

P5A (25.0 mg) and ODI (33.2 mg, 4.0 eq.) were dissolved in chloroform (1.0 mL) and placed in an open 50 mL vial. The solvent was evaporated within 10 min with a Smart Evaporator C1 (Biochromato) at 25 °C. Uncomplexed ODI was washed with cyclohexane and the solid was collected by filtration, followed by drying under vacuum at room temperature. A white crystalline P5A–ODI complex was obtained.

$^1$H NMR spectroscopy for prepared P5A–ODI complex

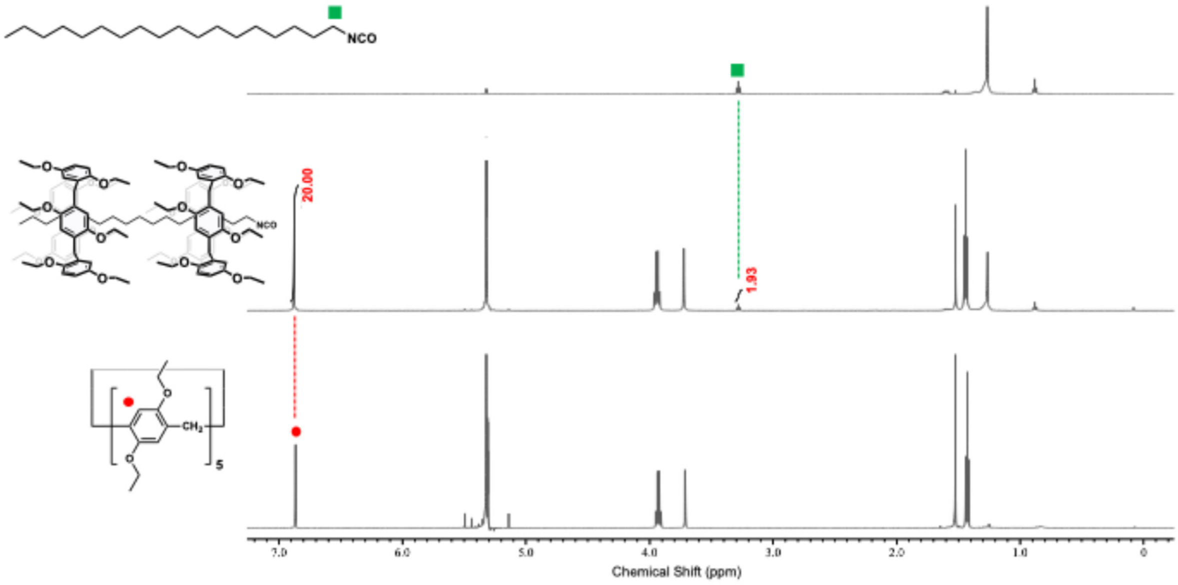


**Supplementary Fig. 28** $^1$H NMR spectra ($CD_2Cl_2$, 298 K) of ODI (top), prepared P5A–ODI complex (middle), and P5A (bottom).

2:1 complexation was confirmed based on the integrals of an aromatic proton on P5A (red square, 20H) and ethylene protons of ODI (green square, 2H).

Examination of the stability of crystalline P5A–ODI complex against water vapor

Crystalline P5A–ODI complex (6.1 mg) was placed in an open 5 mL vial. This vial was then placed inside a 50 mL vial containing water (5.0 mL). The outer vial was tightly capped and stored at room temperature to expose the sample to water vapor. After 3 days, the inner vial was removed and the crystalline complex was dried under vacuum at room temperature.

Stability investigation of P5A–ODI complex

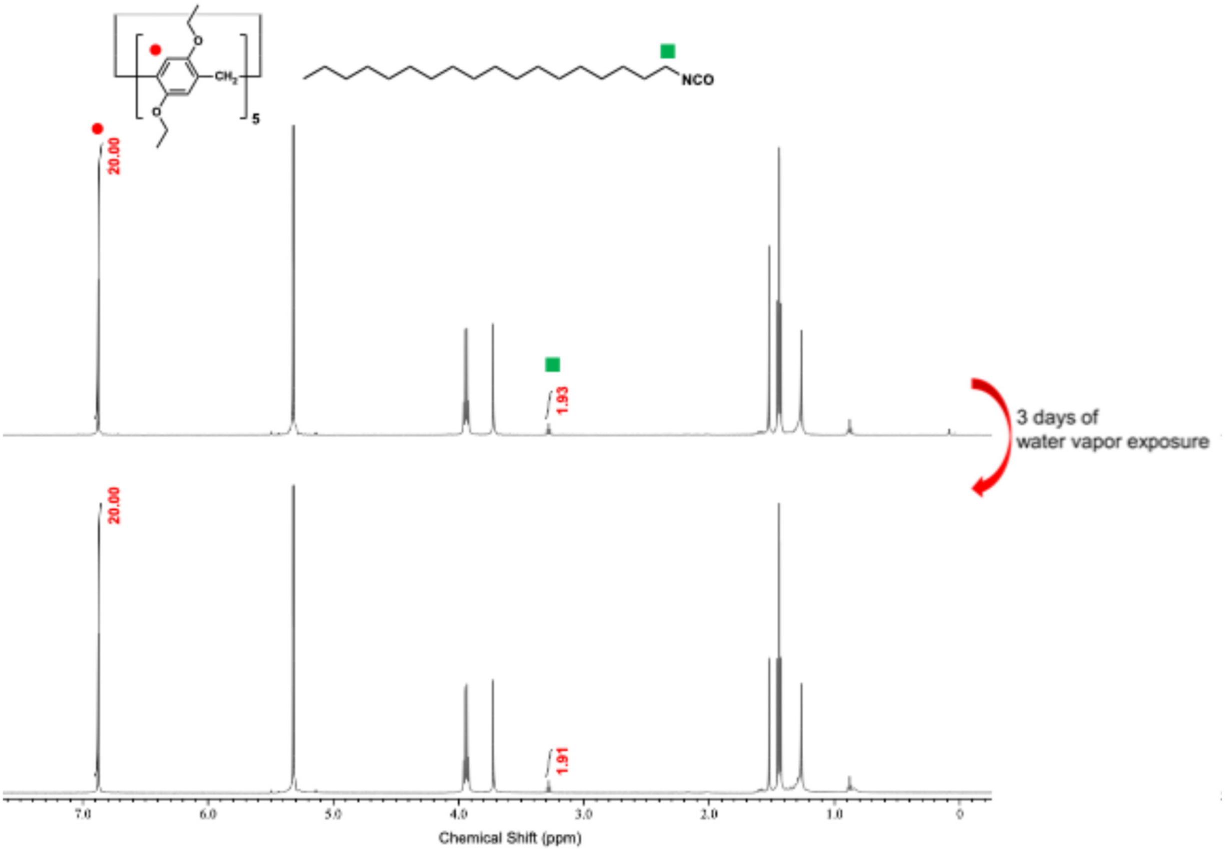


**Supplementary Fig. 29** $^{1}$H NMR spectra ($CD_2Cl_2$, 298 K) of the P5A–ODI complex prepared by evaporation method (top) and after 3 days of water vapor exposure (bottom).

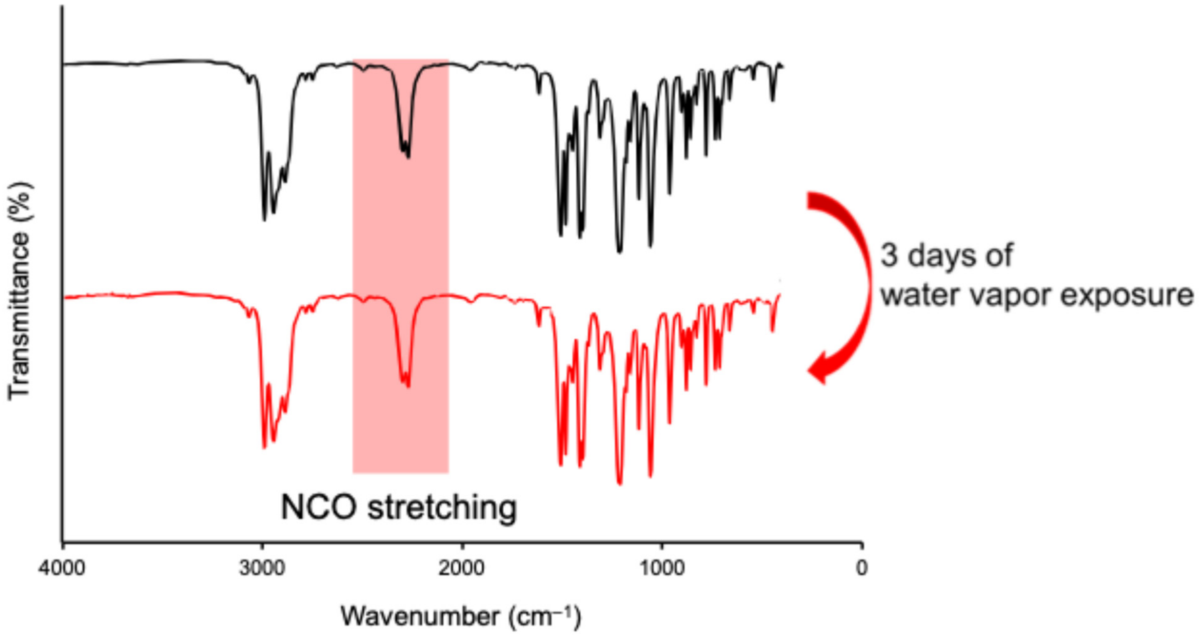


**Supplementary Fig. 30** FT-IR spectra of the P5A–ODI complex prepared by evaporation method (top, black) and after 3 days of water vapor exposure (bottom, red).

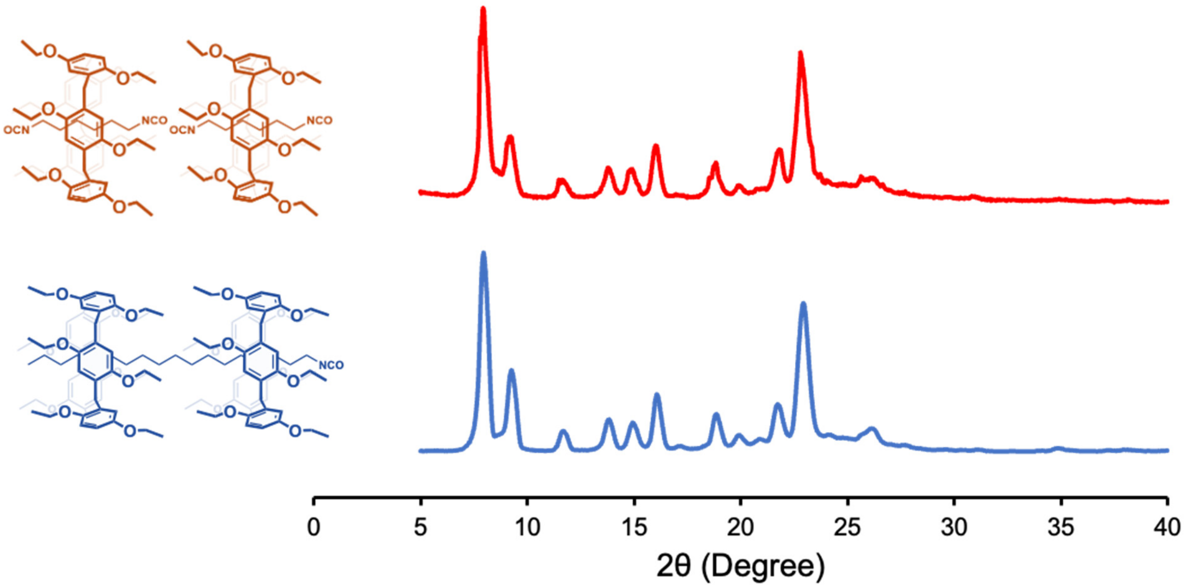


**Supplementary Fig. 31** PXRD patterns of the crystalline P5A–HMDI complex prepared by evaporation method (red, top) and the crystalline P5A–ODI complex prepared by evaporation method (blue, bottom).

The PXRD pattern of P5A–ODI complex was similar to that of P5A–HMDI, suggesting that the crystalline P5A–ODI complex also adopts a 1D channel structure.

Examination of the stability of MDI against water vapor

Methylenediphenyl 4,4'-diisocyanate (MDI) (3.0 mg) was placed in an open 5 mL vial. This vial was then placed inside a 50 mL vial containing water (5.0 mL). The outer vial was tightly capped and stored at room temperature to expose the sample to water vapor. After 3 days, the inner vial was removed and the compounds were dried under vacuum at room temperature. The exposed sample was subsequently analyzed by FT-IR spectroscopy to evaluate the extent of degradation of the isocyanate groups. (Because the exposed sample showed poor solubility, quantitative analysis by $^{1}$H NMR spectroscopy was difficult.)

Stability investigation of MDI

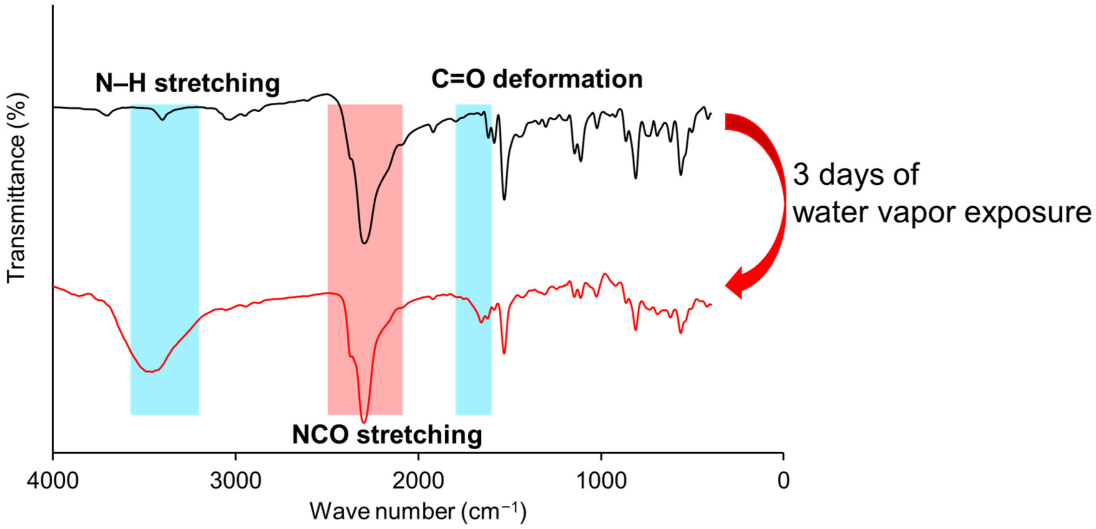


**Supplementary Fig. 32** FT-IR spectra of the MDI (top, black) and after 3 days of water vapor exposure (bottom, red).

Preparation of crystalline P6A–MDI complex (vapor diffusion method)

P6A (20.0 mg) and MDI (50.0 mg, 10.7 eq.) were dissolved in dichloromethane (1.5 mL) and placed in an open 5 mL vial. This vial was then placed inside a 50 mL vial containing diethyl ether (4.0 mL), tightly capped, and stored at room temperature for 1 day to diffuse diethyl ether vapor. After decanting the solution, the solid was washed with diethyl ether, followed by drying under vacuum at room temperature. A white crystalline P6A–MDI complex was obtained.

$^1$H NMR spectroscopy for prepared P6A–MDI complex

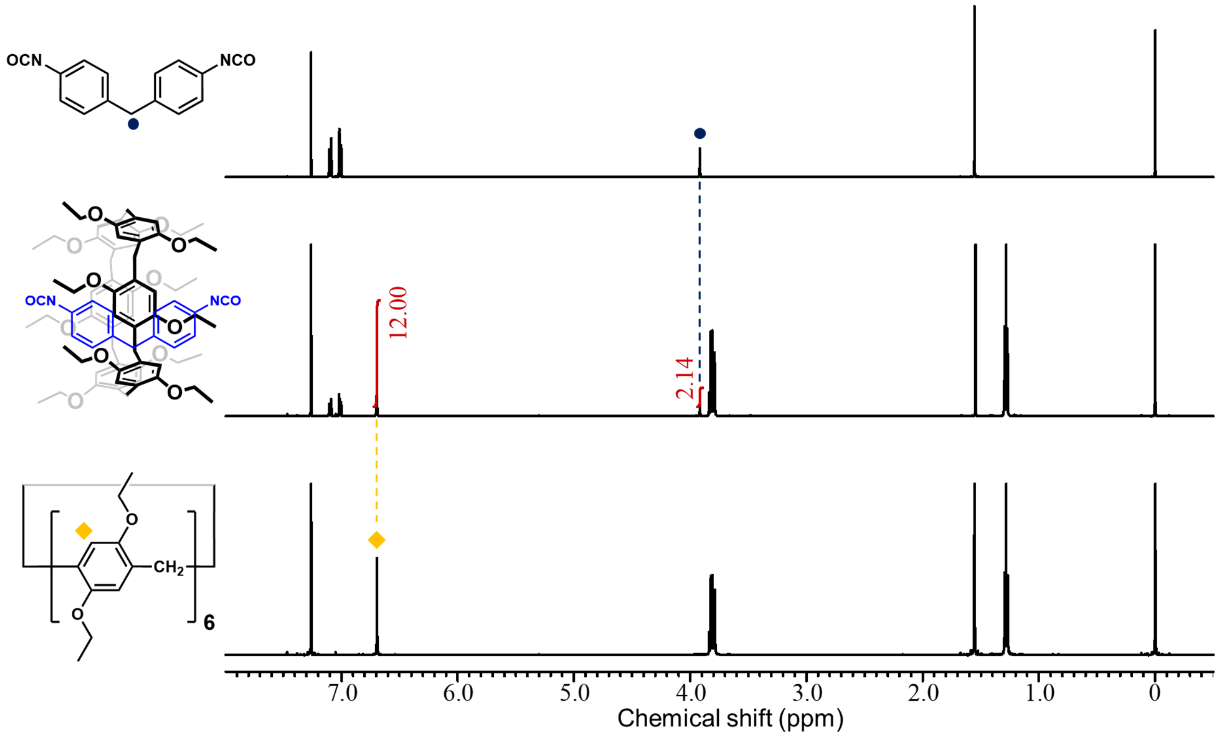


**Supplementary Fig. 33** $^1$H NMR spectra ($CDCl_3$, 298 K) of MDI (top), prepared P6A–MDI complex (middle), and P6A (bottom).

1:1 complexation was confirmed based on the integrals of an aromatic proton on P6A (yellow square, 12H) and ethylene protons of MDI (navy circle, 2H).

Examination of the stability of crystalline P6A–MDI complex against water vapor

Crystalline P6A–MDI complex (3.0 mg) was placed in an open 5 mL vial. This vial was then placed inside a 50 mL vial containing water (5.0 mL). The outer vial was tightly capped and stored at room temperature to expose the sample to water vapor. After 3 days, the inner vial was removed and the crystalline complex was dried under vacuum at room temperature.

Stability investigation of P6A–MDI complex

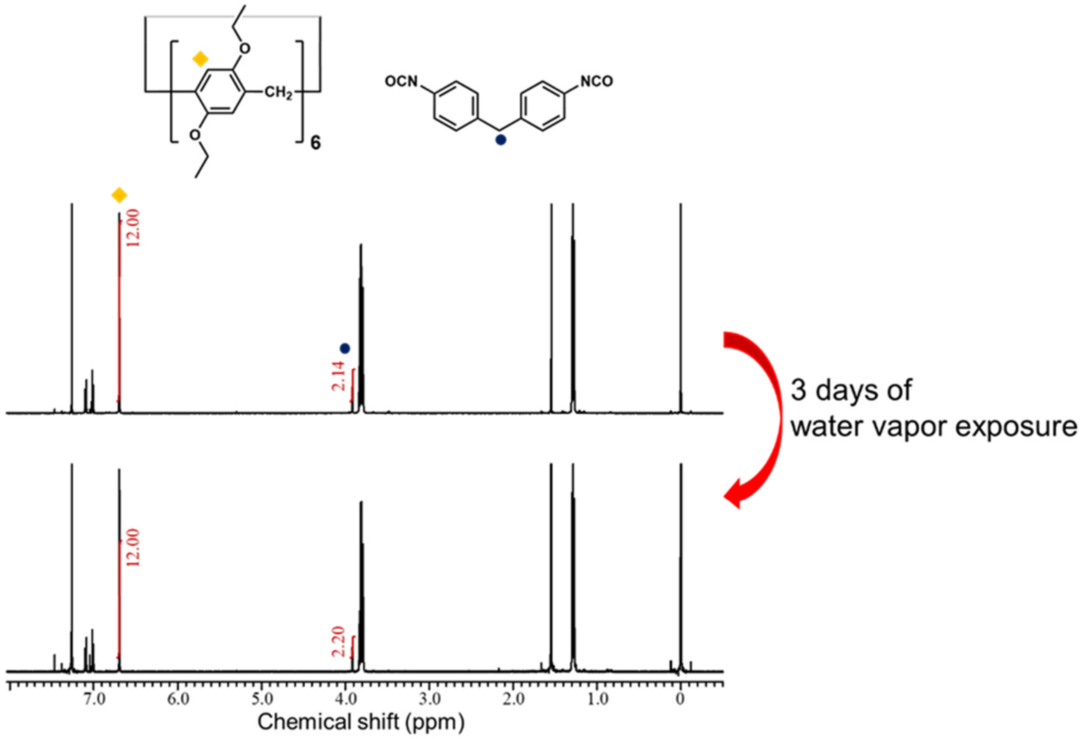


**Supplementary Fig. 34** $^1$H NMR spectra ($CDCl_3$, 298 K) of P6A–MDI complex prepared by vapor diffusion method (top) and after 3 days of water vapor exposure (bottom).

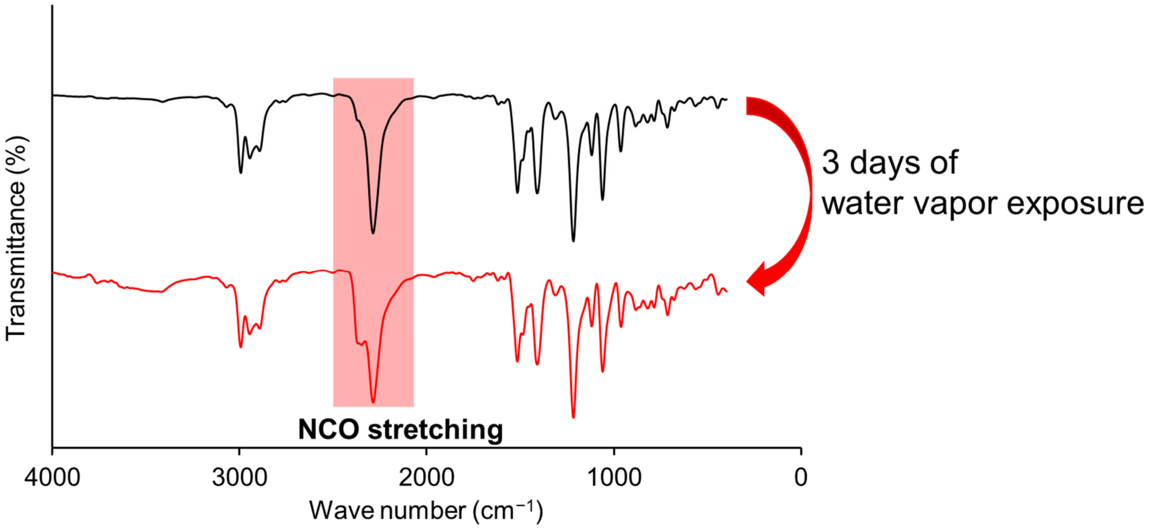


**Supplementary Fig. 35** FT-IR spectra of the P6A–MDI complex prepared by vapor diffusion method (top, black) and after 3 days of water vapor exposure (bottom, red).

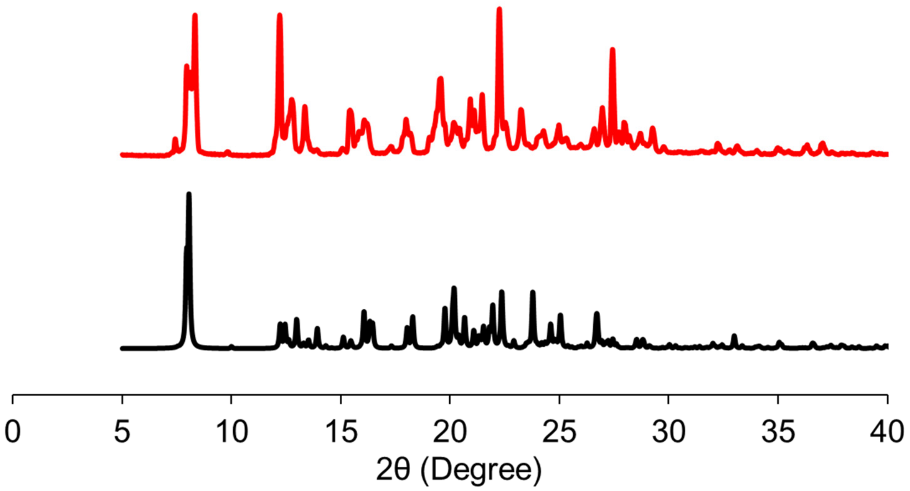


**Supplementary Fig. 36** PXRD patterns of the crystalline P6A–MDI complex prepared by vapor diffusion method (red, top) and the simulated pattern calculated from its single crystal structure (black, bottom).

Examination of the stability of DchDI against water vapor

Dicyclohexylmethane 4,4’-diisocyanate (DchDI) (3.0 mg) was placed in an open 5 mL vial. This vial was then placed inside a 50 mL vial containing water (5.0 mL). The outer vial was tightly capped and stored at room temperature to expose the sample to water vapor. After 3 days, the inner vial was removed and the compounds were dried under vacuum at room temperature. The exposed sample was subsequently analyzed by FT-IR spectroscopy to evaluate the extent of degradation of the isocyanate groups. (Because the exposed sample showed poor solubility, quantitative analysis by $^{1}$H NMR spectroscopy was difficult.)

Stability investigation of DchDI

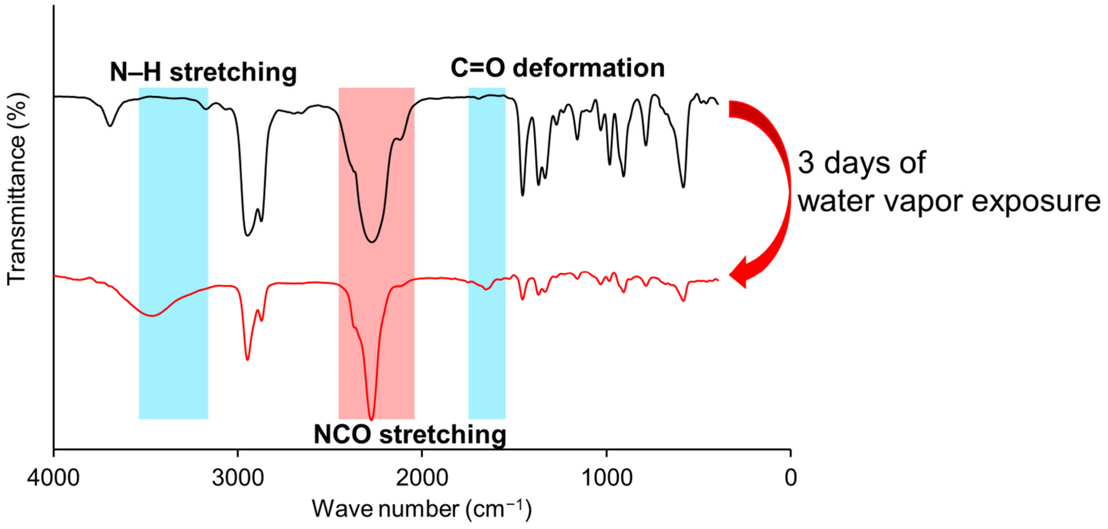


**Supplementary Fig. 37** FT-IR spectra of the DchDI (top, black) and after 3 days of water vapor exposure (bottom, red).

<u>Preparation of crystalline P6A–DchDI complex (vapor diffusion method)</u>

P6A (20.0 mg) and DchDI (50.0 µL, 10.6 eq.) were dissolved in dichloromethane (1.0 mL) and placed in an open 5 mL vial. This vial was then placed inside a 50 mL vial containing diethyl ether (4.0 mL), tightly capped, and stored at room temperature for 2 days to diffuse diethyl ether vapor. After decanting the solution, the solid was washed with diethyl ether, followed by drying under vacuum at room temperature. A white crystalline P6A–DchDI complex was obtained.

<u>$^1$H NMR spectroscopy for prepared P6A–DchDI complex</u>

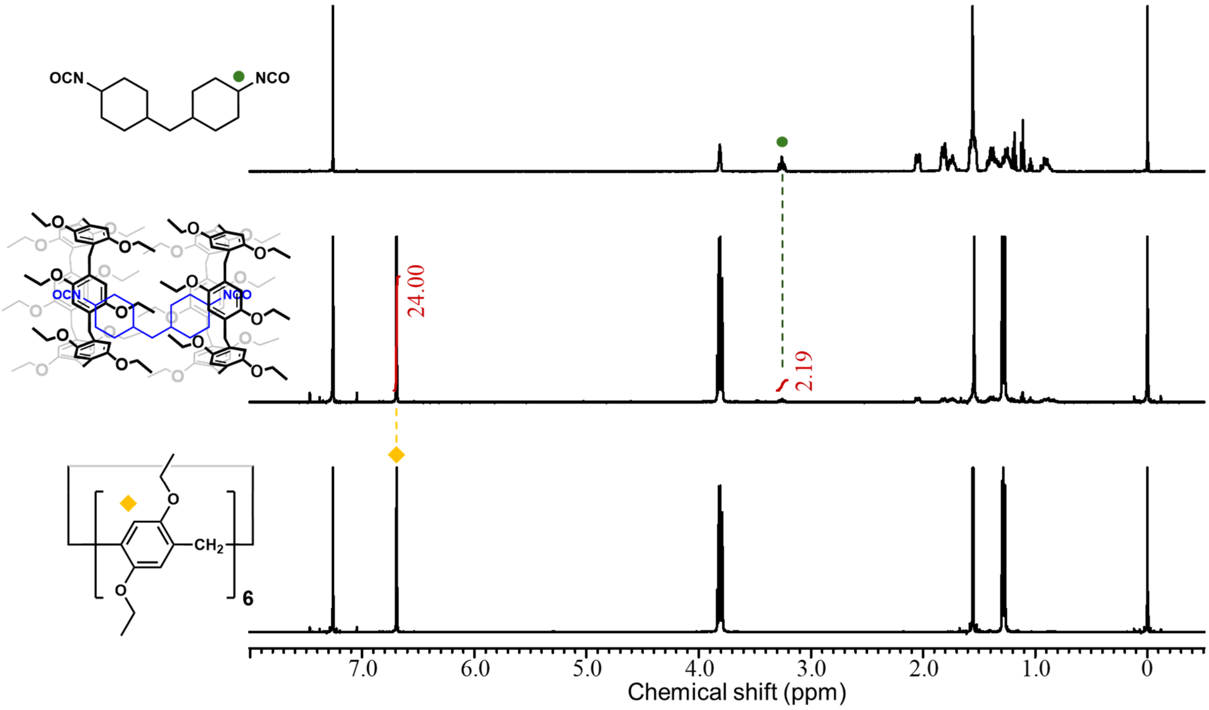


**Supplementary Fig. 38** $^1$H NMR spectra ($CDCl_3$, 298 K) of DchDI (top), prepared P6A–DchDI complex (middle), and P6A (bottom).

2:1 complexation was confirmed based on the integrals of an aromatic proton on P6A (yellow square, 24H) and ethylene protons of DchDI (green circle, 2H).

Examination of the stability of crystalline P6A–DchDI complex against water vapor

Crystalline P6A–DchDI complex (3.3 mg) was placed in an open 5 mL vial. This vial was then placed inside a 50 mL vial containing water (5.0 mL). The outer vial was tightly capped and stored at room temperature to expose the sample to water vapor. After 3 days, the inner vial was removed and the crystalline complex was dried under vacuum at room temperature.

Stability investigation of P6A–DchDI complex

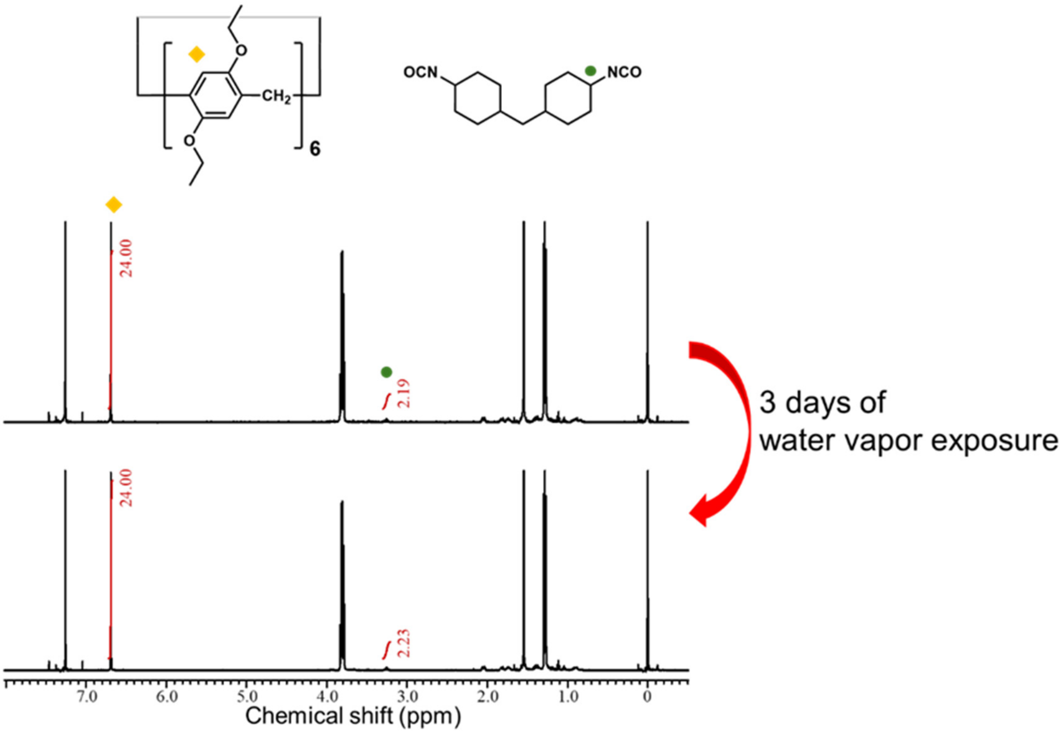


**Supplementary Fig. 39** $^1$H NMR spectra ($CDCl_3$, 298 K) of P6A–DchDI complex prepared by vapor diffusion method (top) and after 3 days of water vapor exposure (bottom).

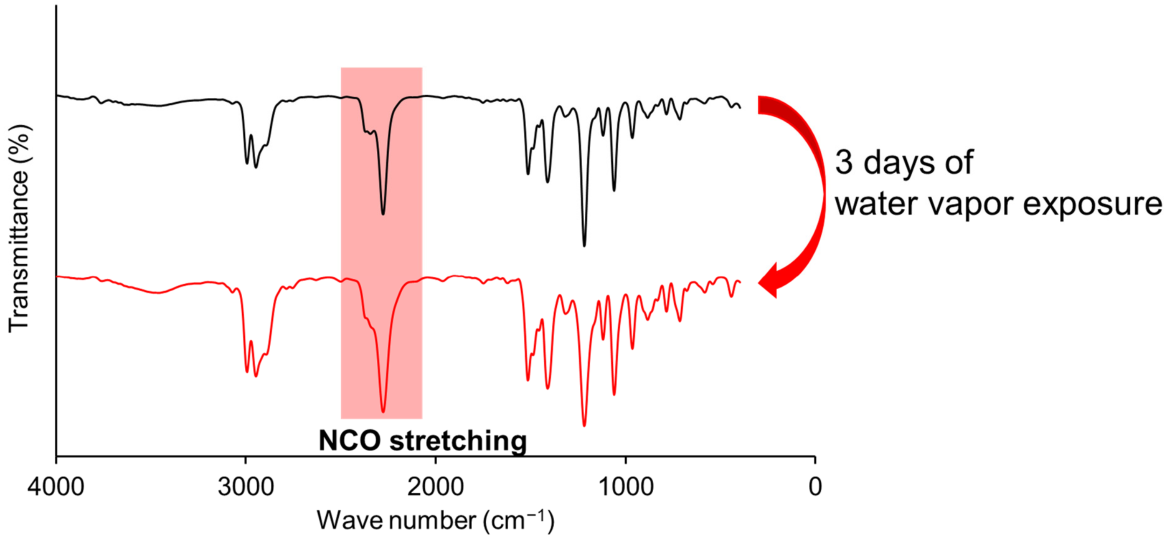


**Supplementary Fig. 40** FT-IR spectra of the crystalline P6A–DchDI complex prepared by vapor diffusion method (top, black) and after 3 days of water vapor exposure (bottom, red).

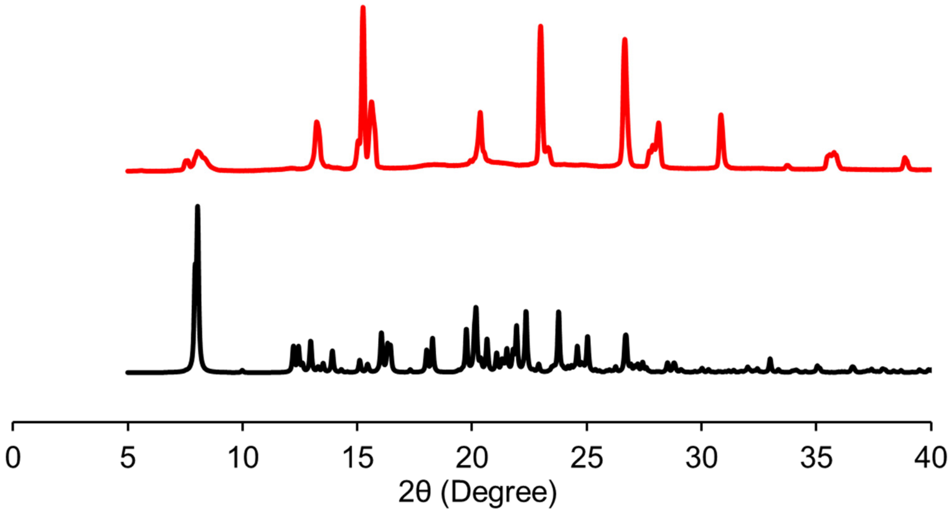


**Supplementary Fig. 41** PXRD patterns of the crystalline P6A–DchDI complex prepared by vapor diffusion method (red, top) and the simulated pattern of P6A–MDI complex calculated from its single crystal structure (black, bottom).

Although the crystalline P6A–DchDI complex were obtained, its structure could not be determined. The crystalline pattern is considered to be different from the 1D channel structure observed for the P6A–MDI complex.

Examination of the stability of XDI against water vapor

*m*-Xylylene diisocyanate (XDI) (3.0 mg) was placed in an open 5 mL vial. This vial was then placed inside a 50 mL vial containing water (5.0 mL). The outer vial was tightly capped and stored at room temperature to expose the sample to water vapor. After 3 days, the inner vial was removed and the compounds were dried under vacuum at room temperature. The exposed sample was subsequently analyzed by FT-IR spectroscopy to evaluate the extent of degradation of the isocyanate groups. (Because the exposed sample showed poor solubility, quantitative analysis by $^{1}$H NMR spectroscopy was difficult.)

Stability investigation of XDI

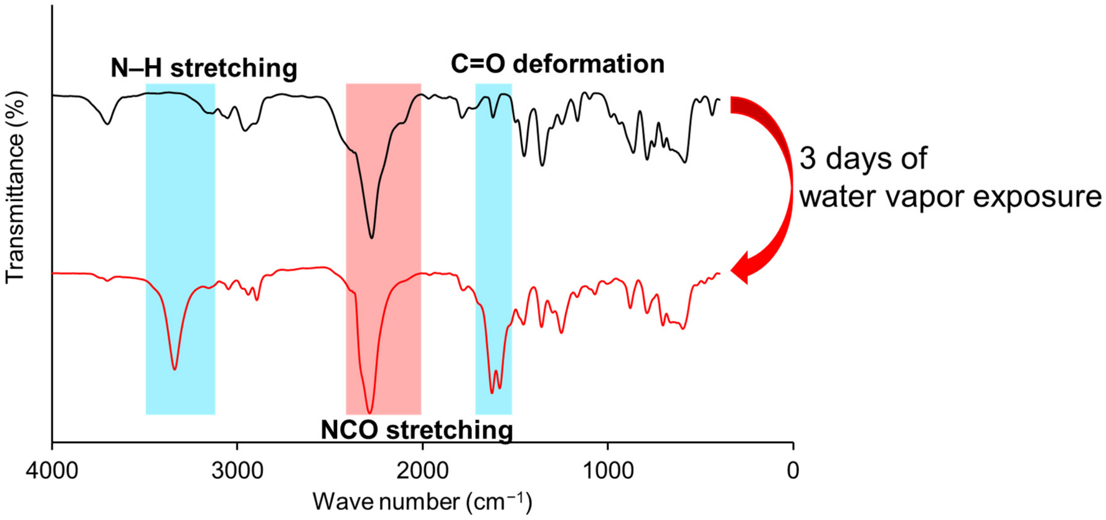


**Supplementary Fig. 42** FT-IR spectra of the XDI （top, black） and after 3 days of water vapor exposure (bottom, red).

Preparation of crystalline P6A–XDI complex (evaporation method)

P6A (20.0 mg) and XDI (29.3 µL) were dissolved in dichloromethane (1 mL) and placed in an open 5 mL vial. The solvent was evaporated within 30 min with Smart Evaporator C1 (Biochromato) at 60 °C. Uncomplexed XDI was washed with hexane and the solid was collected by filtration, followed by drying in vacuum at room temperature. A white crystalline P6A–XDI complex was obtained.

$^1$H NMR spectroscopy for prepared P6A–XDI complex

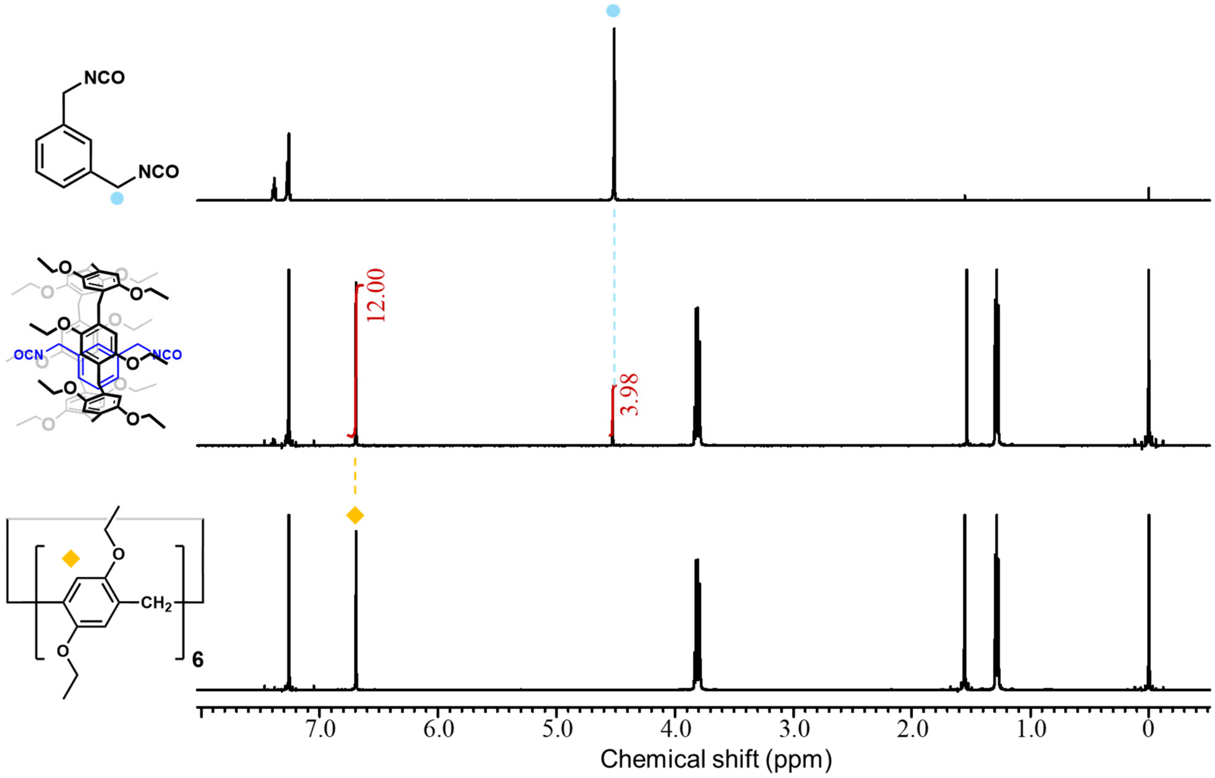


**Supplementary Fig. 43** $^1$H NMR spectra ($CDCl_3$, 298 K) of XDI (top), prepared P6A–XDI complex (middle), and P6A (bottom).

1:1 complexation was confirmed based on the integrals of an aromatic proton on P6A (yellow square, 12H) and ethylene protons of XDI (light blue circle, 4H).

Examination of the stability of crystalline P6A–XDI complex against water vapor

Crystalline P6A–XDI complex (3.0 mg) was placed in an open 5 mL vial. This vial was then placed inside a 50 mL vial containing water (5.0 mL). The outer vial was tightly capped and stored at room temperature to expose the sample to water vapor. After 3 days, the inner vial was removed and the crystalline complex was dried under vacuum at room temperature.

Stability investigation of P6A–XDI complex

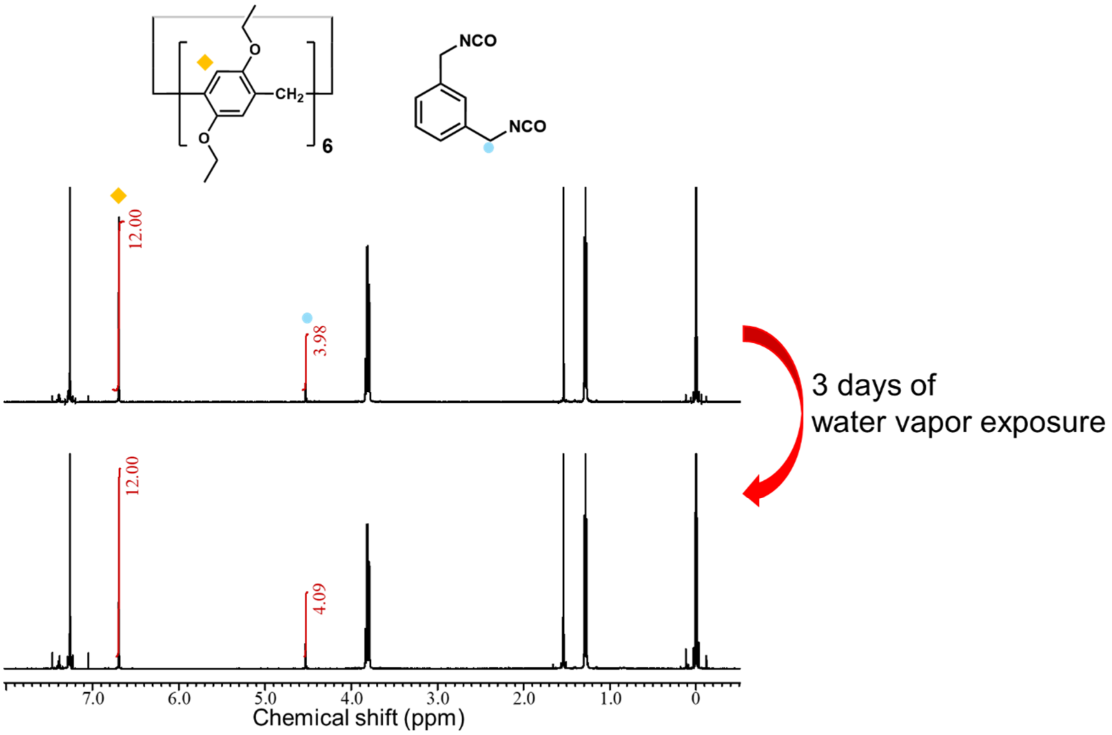


**Supplementary Fig. 44** $^{1}$H NMR spectra ($CDCl_3$, 298 K) of P6A–XDI complex prepared by vapor diffusion method (top) and after 3 days of water vapor exposure (bottom).

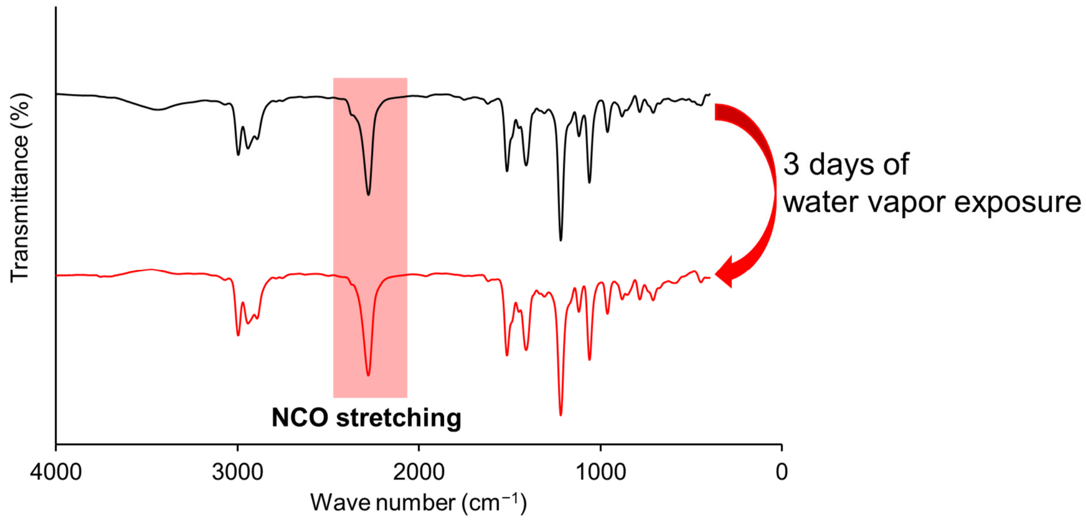


**Supplementary Fig. 45** FT-IR spectra of the P6A–XDI complex prepared by vapor diffusion method (top, black) and after 3 days of water vapor exposure (bottom, red).

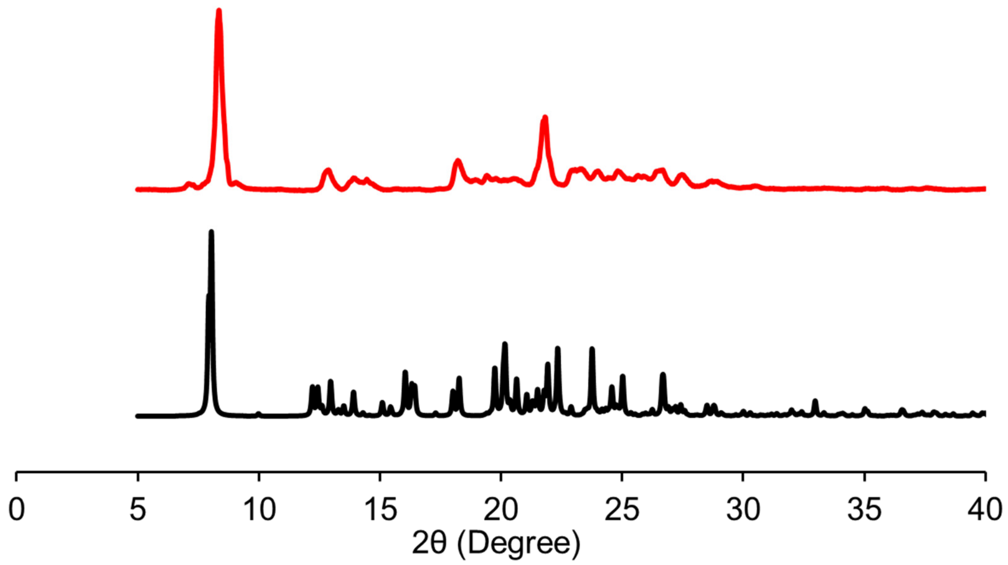


**Supplementary Fig. 46** PXRD patterns of the crystalline P6A–XDI complex prepared by vapor diffusion method (red, top) and the simulated pattern of P6A–MDI complex calculated from its single crystal structure (black, bottom).

The PXRD pattern of P6A–XDI complex was similar to that of P6A–MDI, suggesting that the crystalline P6A–XDI complex also adopts a 1D channel structure.

Examination of the stability of IPDI against water vapor

Isophorone diisocyanate (IPDI) (3.0 mg) was placed in an open 5 mL vial. This vial was then placed inside a 50 mL vial containing water (5.0 mL). The outer vial was tightly capped and stored at room temperature to expose the sample to water vapor. After 3 days, the inner vial was removed and the compounds were dried under vacuum at room temperature. The exposed sample was subsequently analyzed by FT-IR spectroscopy to evaluate the extent of degradation of the isocyanate groups. (Because the exposed sample showed poor solubility, quantitative analysis by $^{1}$H NMR spectroscopy was difficult.)

Stability investigation of IPDI

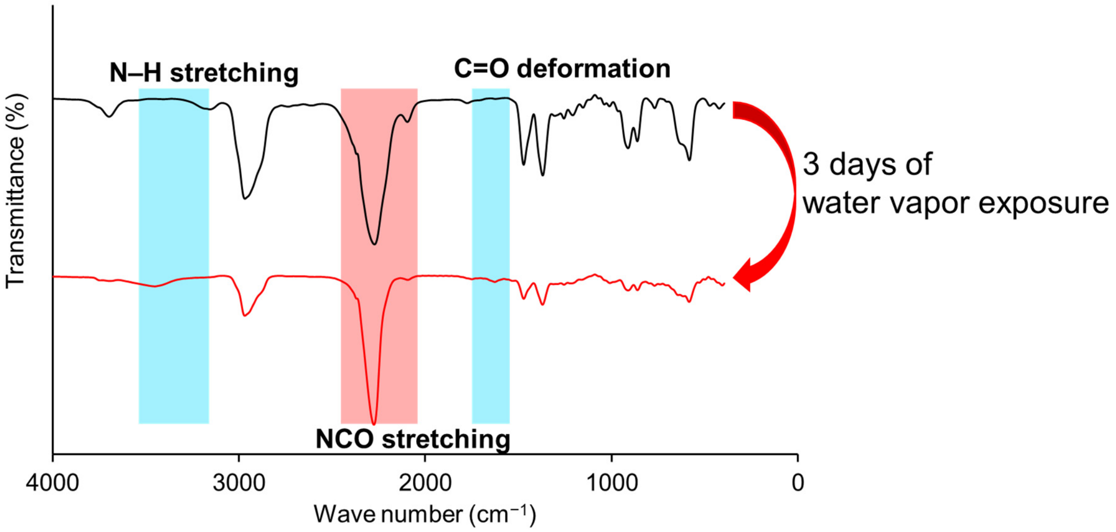


**Supplementary Fig. 47** FT-IR spectra of the IPDI (top, black) and after 3 days of water vapor exposure (bottom, red).

Preparation of crystalline P6A–IPDI complex (evaporation method)

P6A (1.00 g, 1.12 mmol) and IPDI (200 μL, 1.19 mmol) were dissolved in dichloromethane (20 mL) and placed in an open 5 mL vial. The solvent was evaporated within 30 min with Smart Evaporator C1 (Biochromato) at 60 °C. Uncomplexed IPDI was washed with hexane and the solid was collected by filtration, followed by drying in vacuum at room temperature. A white crystalline P6A–IPDI complex was obtained.

$^{1}$H NMR spectroscopy for prepared P6A–IPDI complex

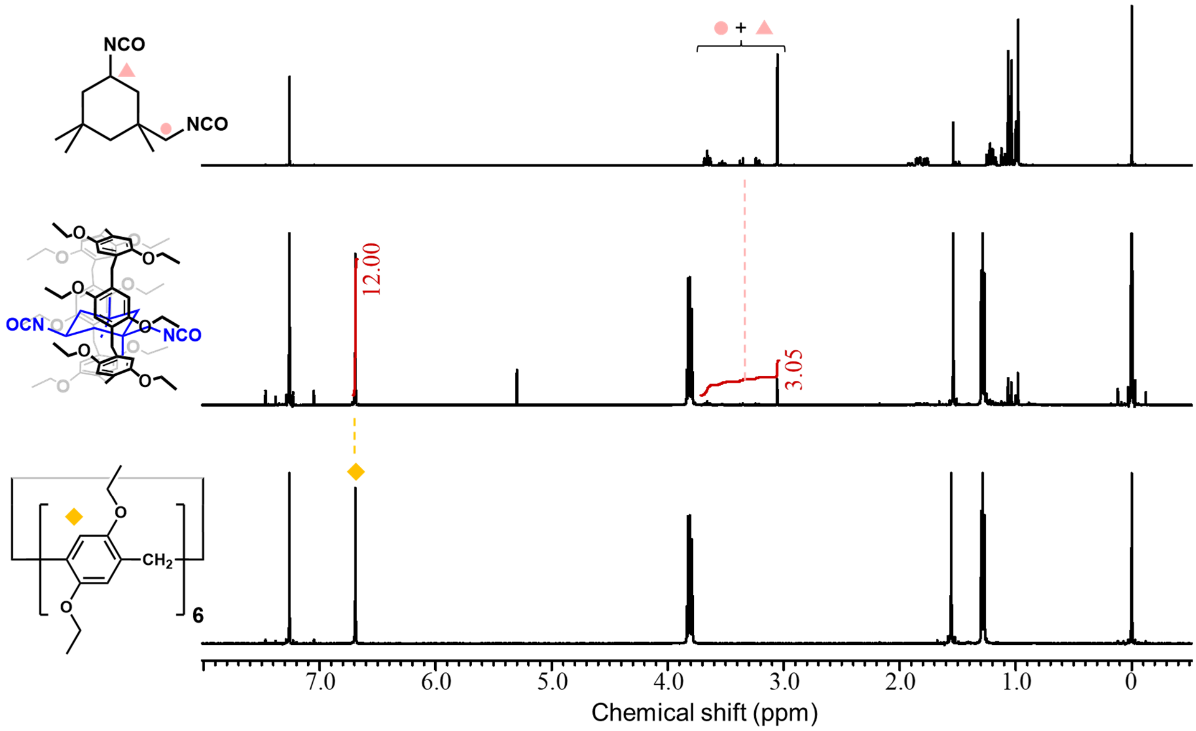


**Supplementary Fig. 48** $^{1}$H NMR spectra ($CDCl_3$, 298 K) of IPDI (top), prepared P6A–IPDI complex (middle), and P6A (bottom).

1:1 complexation was confirmed based on the integrals of an aromatic proton on P6A (yellow square, 12H) and ethylene protons and a methine proton of IPDI (pink circle and triangle,3H).

Examination of the stability of crystalline P6A–IPDI complex against water vapor

Crystalline P6A–IPDI complex (3.0 mg) was placed in an open 5 mL vial. This vial was then placed inside a 50 mL vial containing water (5.0 mL). The outer vial was tightly capped and stored at room temperature to expose the sample to water vapor. After 3 days, the inner vial was removed and the crystalline complex was dried under vacuum at room temperature.

Stability investigation of P6A–IPDI complex

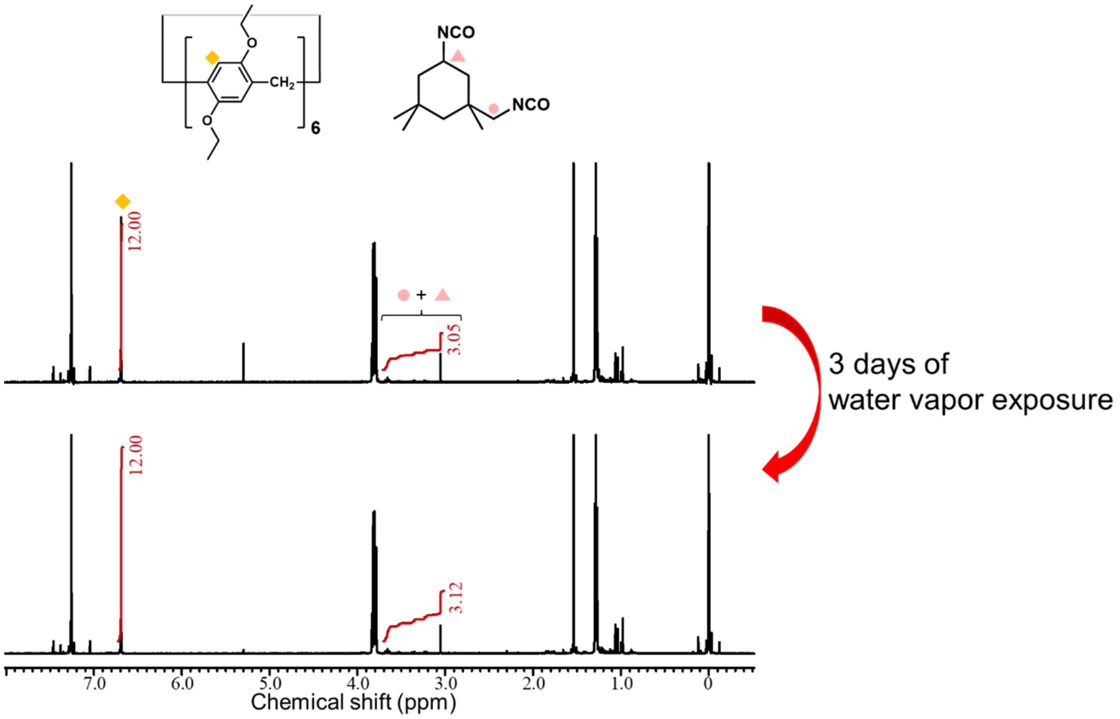


**Supplementary Fig. 49** $^1$H NMR spectra ($CDCl_3$, 298 K) of P6A–IPDI complex prepared by vapor diffusion method (top) and after 3 days of water vapor exposure (bottom).

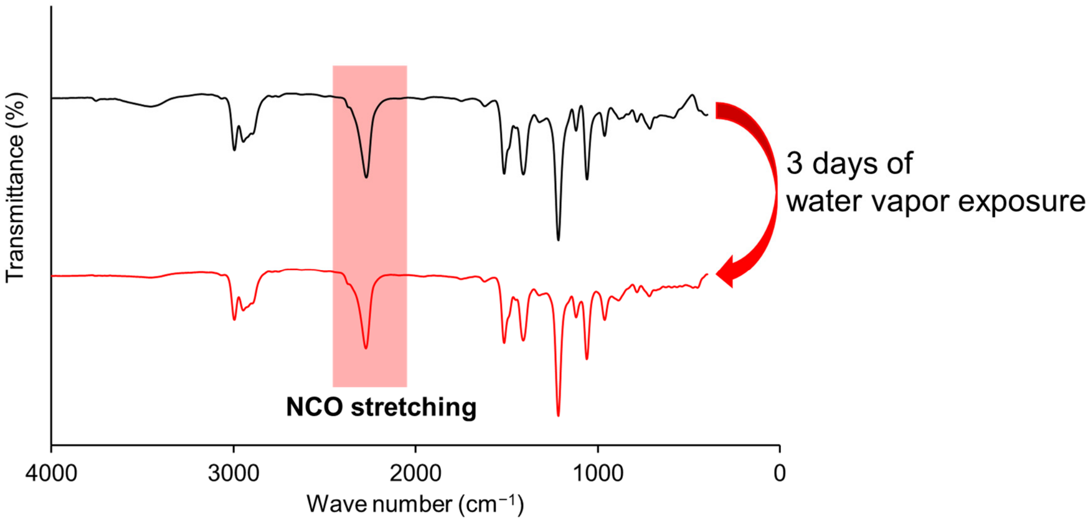


**Supplementary Fig. 50** FT-IR spectra of the P6A–IPDI complex prepared by vapor diffusion method (top, black) and after 3 days of water vapor exposure (bottom, red).

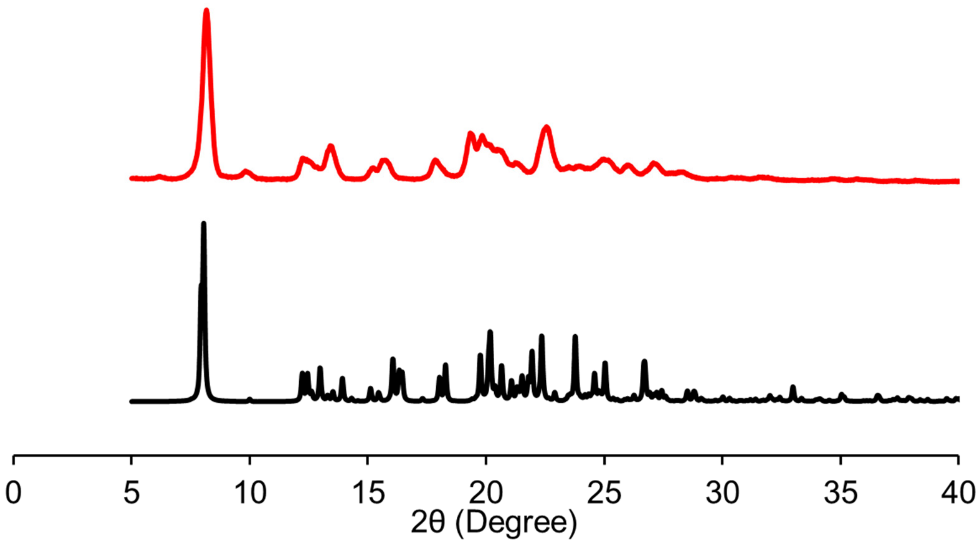


**Supplementary Fig. 51** PXRD patterns of the crystalline P6A–IPDI complex prepared by vapor diffusion method (red, top) and the simulated pattern of P6A–MDI complex calculated from its single crystal structure (black, bottom).

The PXRD pattern of P6A–IPDI complex was similar to that of P6A–MDI, suggesting that the crystalline P6A–IPDI complex also adopts a 1D channel structure.

Since neat IPDI was stable against water vapor (Figure S39), we examined the stability of IPDI against water.

Examination of the stability of IPDI against water

IPDI (3.0 mg) was added into a 5 mL vial containing water (1.0 mL), tightly capped, and stored at room temperature. After 3 days, the solid was collected by evaporation with Smart Evaporator C1 (Biochromato) at 80 °C.

Stability investigation of IPDI

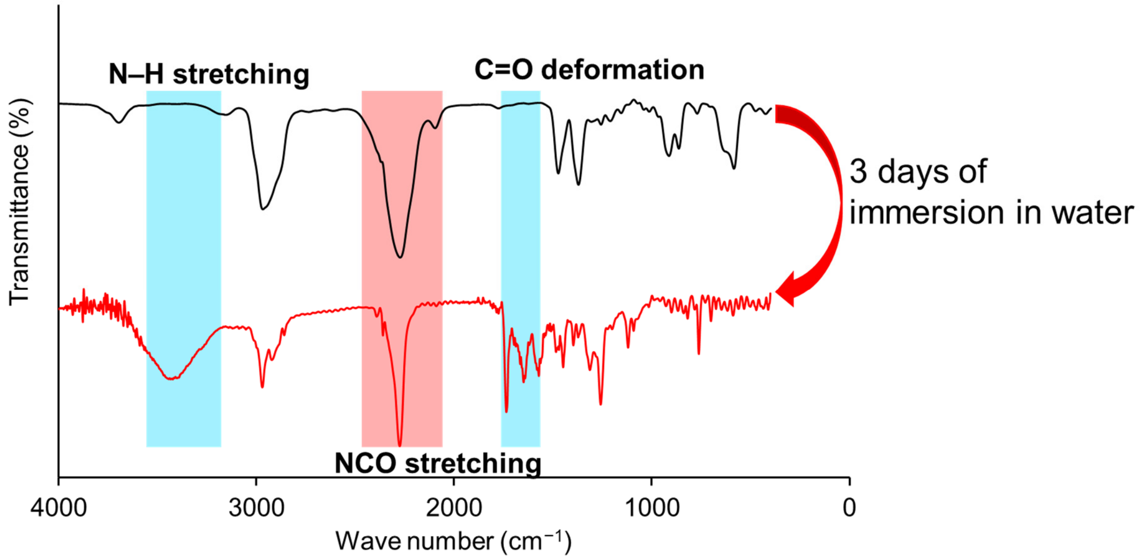


**Supplementary Fig. 52** FT-IR spectra of IPDI (top, black) and after 3 days of immersion in water (bottom, red).

Examination of the stability of crystalline P5A–IPDI complex against water

Crystalline P5A–IPDI complex (3.0 mg) was added into a 5 mL vial containing water (1.0 mL), tightly capped, and stored at room temperature. After 3 days, the crystals were collected by filtration.

Stability investigation of P6A–IPDI complex

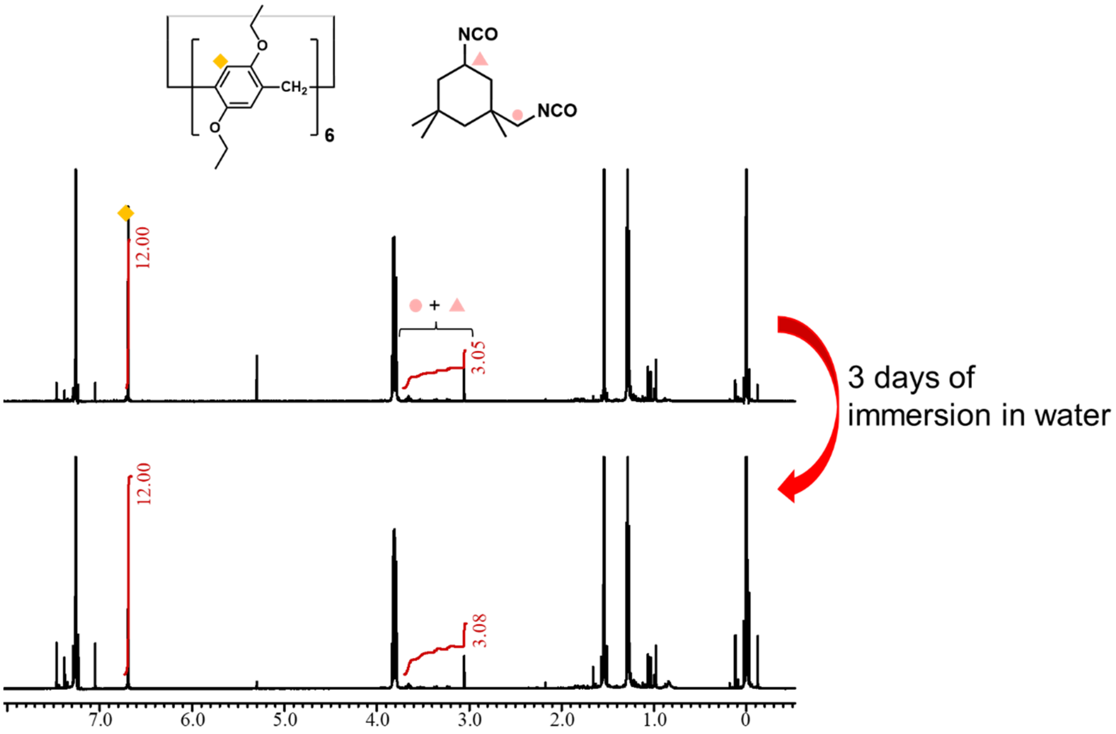


**Supplementary Fig. 53** $^{1}$H NMR spectra ($CDCl_3$, 298 K) of the P6A–IPDI complex prepared by evaporation method (top) and after 3 days of immersion in water (bottom).

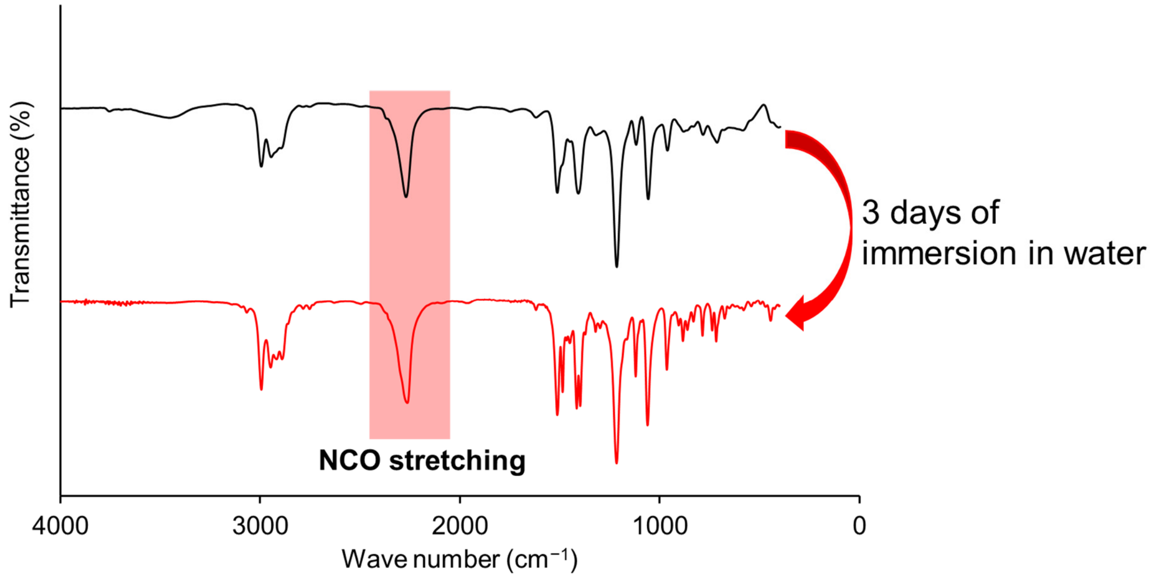


**Supplementary Fig. 54** FT-IR spectra of the P6A–IPDI complex prepared by evaporation method (top, black) and after 3 days of immersion in water (bottom, red).

The FT-IR and $^1$H NMR measurements revealed that while IPDI decomposed under the immersion in water (Figure S44), the P6A–IPDI complex remained stable (Figure S45, S46).

Attempted preparation of crystalline P5A–TESPI complex

P5A (20.0 mg) and 3-(triethoxysilyl)propyl isocyanate (TESPI) (55.5 µL, 10 eq.) were dissolved in chloroform (1.0 mL) and placed in an open 5 mL vial. The solvent was evaporated within 30 min with Smart Evaporator C1 (Biochromato) at 80 °C. Uncomplexed TESPI was washed with cyclohexane and the solid was collected by filtration, followed by drying in vacuum at room temperature. A white crystalline product was obtained.

$^{1}$H NMR spectroscopy for the crystalline product

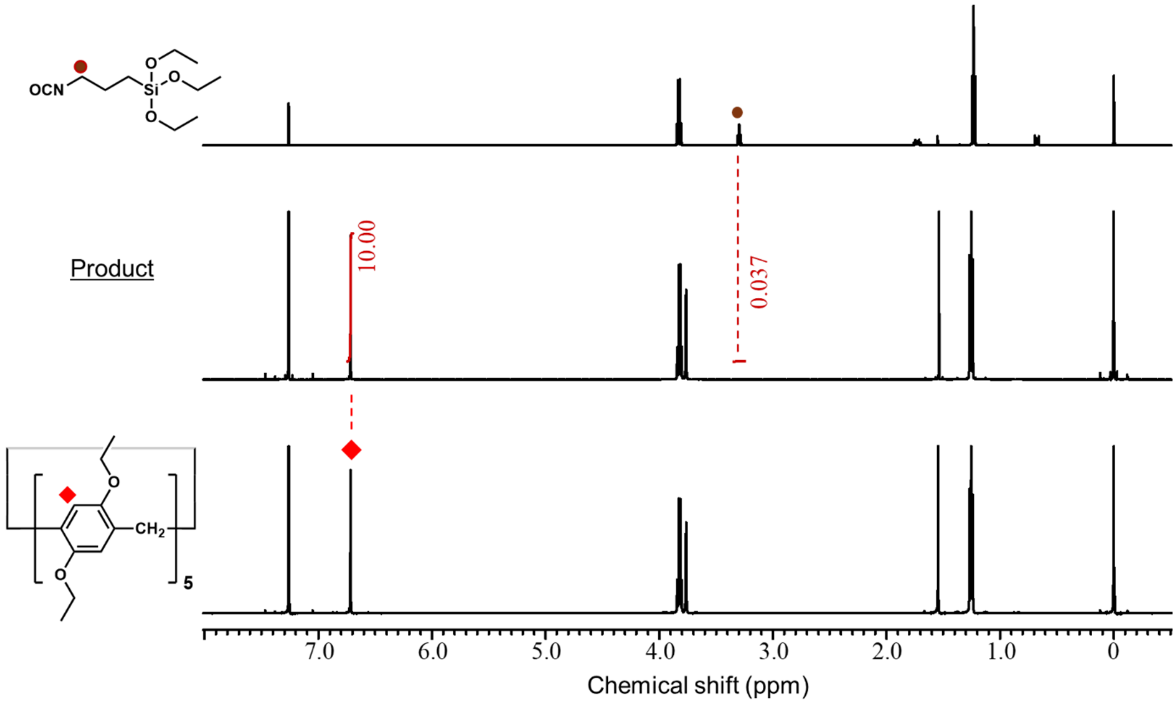


**Supplementary Fig. 55** $^{1}$H NMR spectra ($CDCl_3$, 298 K) of TESPI (top), crystalline product (middle), and P5A (bottom).

If a 1:1 complex were formed, ethylene protons of TESPI (brown circle) would be expected to appear as a 2H signal; however, the corresponding integral was negligible, indicating that P5A and TESPI did not form a complex.

Attempted preparation of crystalline P6A–TESPI complex

P6A (20.0 mg) and TESPI (46.3 µL, 10 eq) were dissolved in dichloromethane (1.0 mL) and placed in an open 5 mL vial. The solvent was evaporated within 30 min with Smart Evaporator C1 (Biochromato) at 60 °C. Uncomplexed TESPI was washed with hexane and the solid was collected by filtration, followed by drying in vacuum at room temperature. A white crystalline product was obtained.

$^1$H NMR spectroscopy for the crystalline product

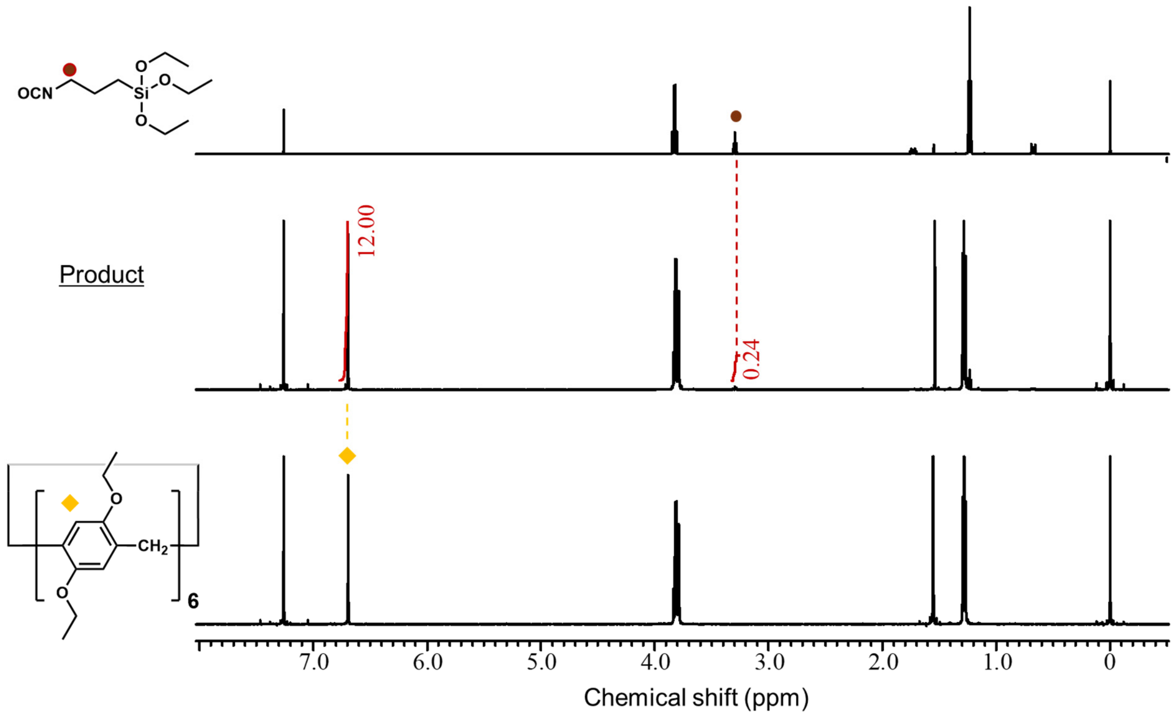


**Supplementary Fig. 56** $^1$H NMR spectra ($CDCl_3$, 298 K) of TESPI (top), crystalline product (middle), and P6A (bottom).

If a 1:1 complex were formed, ethylene protons of TESPI (brown circle) would be expected to appear as a 2H signal; however, the corresponding integral was negligible, indicating that P6A and TESPI did not form a complex.

Attempted preparation of crystalline P6A–BTMPI complex

P6A (20.0 mg) and 3,5-bis(trifluoromethyl)phenyl isocyanate (BTMPI) (32.0 μL, 10 eq) were dissolved in dichloromethane (1.0 mL) and placed in an open 5 mL vial. The solvent was evaporated within 30 min with Smart Evaporator C1 (Biochromato) at 60 °C. Uncomplexed BTMPI was washed with hexane and the solid was collected by filtration, followed by drying in vacuum at room temperature. A white crystalline product was obtained.

$^1$H NMR spectroscopy for the crystalline product

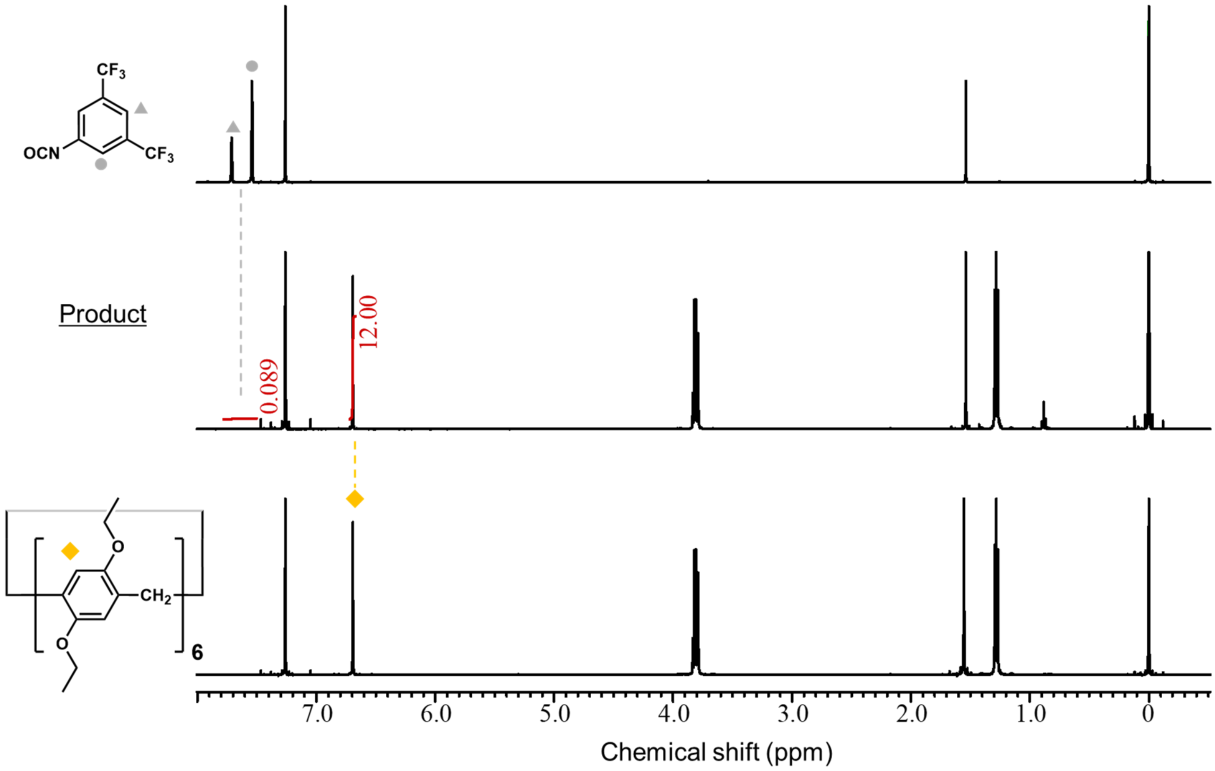


**Supplementary Fig. 57** $^1$H NMR spectra ($CDCl_3$, 298 K) of BTMPI (top), crystalline product (middle), and P6A (bottom).

If a 1:1 complex were formed, aromatic protons of BTMPI (gray circle) would be expected to appear as a 3H signal; however, the corresponding integral was negligible, indicating that P6A and BTMPI did not form a complex.

## 6. X-ray Crystallographic Analysis

X-ray crystallographic analysis of P5A–HMDI

Intensity data were collected on a Rigaku Saturn 724+ with MicroMax-007HF CCD diffractometer with Varimax Mo optics using graphite-monochromated MoKα radiation. The structures were solved and refined by full-matrix least-squares procedures based on $F^2$ (SHELXL-2018/3).

Single crystals of P5A–HMDI were prepared by vapor diffusion from the chloroform solution under *c*-hexane atmosphere at room temperature.

**Table S1.** Crystallographic data of P5A–HMDI at 143 K

| | |
|---|---|
| Morphology | Colorless prism |
| Crystal system | monoclinic |
| Space group | $P2_1/n$ |
| $a$/Å | 12.586(4) |
| $b$/Å | 20.694(7) |
| $c$/Å | 22.836(9) |
| $\alpha$/deg | 90 |
| $\beta$/deg | 91.515(7) |
| $\gamma$/deg | 90 |
| $V$/Å$^3$ | 5946(4) |
| $Z$ | 4 |
| Density/g cm$^{-3}$ | 1.183 |
| μ(Mo-K$\alpha$) /mm$^3$ | 0.081 |
| $F$(000) | 2280.0 |
| 2$\theta$ range for data collection | 6.170–55.020 |
| Limiting indices | $-13 \le h \le 16, -24 \le k \le 26, -29 \le l \le 29$ |
| Reflections collected (unique) | 44277/13155 [$R$(int) = 0.0581] |
| Goodness-off-fit on $F^2$ | 1.112 |
| Final $R$ indices [$I > 2\sigma$(I)] | $R_1$ = 0.0980, w$R_2$ = 0.2423 |
| $R$ indices (all data) | $R_1$ = 0.1427, w$R_2$ = 0.2768 |
| $T$/K | 143 |

X-ray crystallographic analysis of P6A–MDI

Intensity data were collected on a Bruker Venture diffractometer with using graphite-monochromated CuKα radiation. The structures were solved by SHELXD and refined by full-matrix least-squares procedures based on $F^2$ (SHELXL-2014).

Single crystals of P6A–MDI were prepared by vapor diffusion from the dichloromethane solution under diethylether atmosphere at room temperature.

**Table S2.** Crystallographic data of P6A–MDI at 153 K

| | |
|---|---|
| Morphology | Colorless prism |
| Crystal system | monoclinic |
| Space group | $C2/c$ |
| $a$/Å | 26.7295(8) |
| $b$/Å | 12.6560(4) |
| $c$/Å | 24.7525(11) |
| $\alpha$/deg | 90 |
| $\beta$/deg | 118.7169(10) |
| $\gamma$/deg | 90 |
| $V$/Å$^3$ | 7343.6(5) |
| $Z$ | 4 |
| Density/g cm$^{-3}$ | 1.194 |
| μ(Cu-K$\alpha$) /mm$^3$ | 0.651 |
| $F$(000) | 2824.0 |
| 2$\theta$ range for data collection | 7.542–133.152 |
| Limiting indices | $-31 \leq h \leq 27$, $-15 \leq k \leq 15$, $-29 \leq l \leq 29$ |
| Reflections collected (unique) | 6401/5838 [$R$(int) = 0.0327] |
| Goodness-off-fit on $F^2$ | 1.067 |
| Final $R$ indices [$I > 2\sigma$(I)] | $R_1$ = 0.0597, w$R_2$ = 0.1527 |
| $R$ indices (all data) | $R_1$ = 0.0637, w$R_2$ = 0.1567 |
| $T$/K | 153 |

## 7. References


[S1] Ogoshi, T., Sueto, R., Yagyu, M., Kojima, R., Kakuta, T., Yamagishi, T., Doitomi, K., Tummanapelli, A. K., Hirao, H., Sakata, Y., Akine, S. & Mizuno, M. Molecular weight fractionation by confinement of polymer in one-dimensional pillar[5]arene channels. *Nat. Commun*. **10**, 479 (2019).

[S2] Huang, S., Toh, C. L., Yang, L.P., Phua, S., Zhou, R., Dasari, A., & Lu, X. H. Reinforcing nylon 6 via surface-initiated anionic ring-opening polymerization from stacked-cup carbon nanofibers. *Compos. Sci. Technol*. **93**, 30-37 (2014).

[S3] Inada, Y., & Masuda, H. *Kansai Paint Co., Ltd.* Method of forming multilayered coating films using starch-based colored base coatings, the multilayered coating films with good interlayer adhesion therefrom, and their coated articles. *WO 2009075369 A1* (2009).
