# Supplementary material for: Supramolecular protection of isocyanates from water by encapsulation within hydrophobic crystalline pillar[n]arene macrocycles
Source: Nat Commun. 2026 May 9;17:6274. doi: 10.1038/s41467-026-72696-y (PMC13376218; doi:10.1038/s41467-026-72696-y)
Supplement: Supplementary file 2 — Transparent Peer Review file [file 41467_2026_72696_MOESM2_ESM.pdf]

nature portfolio

Peer Review File

Supramolecular Protection of Isocyanates from Water by Encapsulation within Hydrophobic Crystalline Pillar[*n*]arene Macrocycles

Corresponding Author: Professor Tomoki Ogoshi

This file contains all reviewer reports in order by version, followed by all author rebuttals in order by version.

Version 0:

Reviewer comments:

Reviewer #1

(Remarks to the Author)
The manuscript describes the successful implementation of supramolecular protection strategy towards isocyanate compounds that are known to be unstable in the presence of water (also atmospheric moisture). The non-covalent protection of isocyanates occurs only in the crystalline state through their inclusion into the macrocyclic cavities of per-ethylated pillar[n]arene macrocycles. Such solid state host-guest assemblies are highly hydrophobic providing stabilization and a mean for long-term storage for isocyanate compounds. The deprotection is achieved simply by dissolution the host-guest crystals, as no complexation in the solution has been observed by NMR. The method itself has some limitation, as good host-guest complementarity is required between specific isocyanate guest and macrocyclic host. As the authors discuss, more bulky isocyanates are not suitable guests for either pillar[5]arene or pillar[6]arene and thus could not be protected. Still, in future it seems to be possible to match even bulkier isocyanates with suitable hosts, not necessarily of pillar[n]arene family. Taking into account the importance of di-isocyanate compounds in the production of polyurethane polymers, the demonstrated potential for practical application, together with possible extension of supramolecular protection concept onto other water-sensitive reagents, I would recommend the paper to be published after some minor revision (see below).
Comments:
1) Label of Fig. 3… i, j, X-ray crystal structure of the P5A–HMDI complex in the ab plane (i, space filling model, the solvent molecules have been removed for clarity) and bc plane (j, capped sticks model, the solvent molecules and hydrogen atoms have been removed for clarity).

The crystal structure model of P5A–HMDI as appears from CIF file does not comprise any solvent molecules to be omitted. The same comment concerns label of Fig.5d,e and crystal structure of P6A-MDI. Please, correct.

2) The P5A–HMDI crystal structure model has misplaced hydrogen atoms on C01P carbon atom, which is part of disordered ethyl group. Hence, the erroneous formula for pillar[5]arene given as C55 H69.41 O10 in CIF file. The proper formula should be C55 H70 O10.
The handling of disorder of HMDI molecule included in the P5A cavity is far from optimal. Very harsh EADP constraints and ISOR restraints have been used excessively, that can implement a bias in the structure model. EADP and ISOR should be used very carefully when other measures have been unsuccessful (combination of SIMU and RIGU) as the last resort only. The minor component of disorder can also be refined isotropically. The same applies for disordered O-ethyl substituents of pillar[5]arene. The implementation of all restraints and constraints should be clearly explained in the manuscript text (Supp. info). The problematic lines in the res file are, for example:
EADP N5A N5
EADP C5A C5
EADP O13A O13
EADP C8A C8
EADP N12A N12
EADP C6A C6
EADP O24 O24A
EADP C9A C3
EADP C3 C9

EADP C9 C9A
EADP C3A C3
ISOR 0.01 0.02 O13A O13 C5 C5A N5 N5A C9A C9 C3 C3A C1A C1 C2A C2 C4A C4 =
C8A C8 N12 N12A C6 C6A O24 O24A

Overall, the structure should be re-refined with correct placement of hydrogen atoms and reconsidered disorder modelling. The new CIF file should be re-deposited in the CSD.

3) The refinement strategy for symmetry-related disorder of MDI guest in the pillar[6]arene cavity could be improved. Instead of the combination of DELU and ISOR as used by authors, the SIMU/RIGU should be more appropriate.

DELU 0.003 C34 > LAST
ISOR 0.015 C34 > LAST

Also, many DFIX and SADI can be simply substituted by AFIX 66 for the geometry of aromatic rings.

DFIX 1.39 C35 C36 C36 C37 C37 C38 C38 C39 C39 C40 C40 C35
SADI C35 C37 C36 C38 C37 C39 C38 C40 C39 C35 C40 C36
DFIX 1.39 C42 C43 C43 C44 C44 C45 C45 C46 C46 C47 C47 C42
SADI C42 C44 C43 C45 C44 C46 C45 C47 C46 C42 C47 C43

Oksana Danylyuk

Reviewer #2

(Remarks to the Author)
The manuscript describes the use of pillararenes as supramolecular hosts to protect bis-isocyanates in the solid state, enabling their long-term storage. The authors convincingly demonstrate, through NMR, IR, and single-crystal X-ray diffraction analyses, that the bis-isocyanates are encapsulated within the cavity of the pillararenes, which assemble into hydrophobic channels in the crystalline state. This supramolecular protection strategy enables the stabilization of highly moisture-sensitive species such as isocyanates by shielding them from ambient humidity.

A key advantage over conventional covalent protection strategies lies in the reversibility of the process: upon dissolution, supramolecular deprotection occurs rapidly and quantitatively, without the need for additional reagents, thereby restoring the intrinsic reactivity of the bis-isocyanates. The authors further demonstrate the synthetic utility of this concept through its application to polyurethane synthesis.

Overall, the study is convincing and will be of interest to researchers in supramolecular chemistry and beyond. Regarding conceptual originality, to the best of my knowledge, there are no closely related reports involving supramolecular protection using polyaromatic cavity-based macrocycles such as pillararenes. However, the broader concept of solid-state stabilization via host–guest chemistry is less unprecedented in the context of cyclodextrins, which have been extensively investigated for stabilizing and suppressing the reactivity of neutral guests in the solid state.

At first glance, the generality of the concept appears somewhat limited. Pillar[5]arene derivatives can encapsulate only linear bis-isocyanates within a quite narrow chain-length window. Larger oligomers such as pillar[6]arenes accommodate a broader range of guests, yet guest size and geometry remain constrained by cavity dimensions. In addition, pillar[6]arenes remain comparatively expensive to produce. Expanding the methodology to pillararenes of different cavity sizes and, critically, optimizing host recyclability (the reported recovery yield of 73% remains modest) will be essential steps toward improving the generality and practical viability of the approach.

Despite these limitations, I believe the manuscript merits publication in a journal such as Nature Communications, provided the authors address the following points:

1. The description of the solution-state complexation behavior is imprecise and at times contradictory. For example, the Introduction states: “because complexation only occurs in the crystalline state”, whereas the caption of Figure 1 reads: “Because the binding affinity of the complex is weak in the reaction solvent…”. These statements are not inherently incompatible but lack sufficient clarification. It is evident that complexation is solvent-dependent: in DCM, no complexation is observed due to competitive inclusion of the solvent, whereas in CHCl3, weak complexation occurs because the solvent is too bulky to fit efficiently within the cavity. Similar ambiguities appear in the discussion of pillar[6]arene in different solvents (toluene, DCM, etc.). In other words, the authors should provide a more rigorous and solvent-specific description of binding behavior and avoid overly general statements.

2. Figure 1 lacks clarity and scientific rigor in its current form:
- In step e, the guest should be indicated on the reaction arrow, and polymer separation should be explicitly depicted.
- In step c, the solvent (rather than the complex) should appear above the arrow.
- The notation “R′” is inappropriate; “X” would be clearer.
- The central image unnecessarily complicates the scheme and applies only to pillar[5]arenes.

A complete redesign of this figure is recommended.

3. “Fig. 3f” should be corrected to “Fig. 3k” in the sentence:
“Thermogravimetric analysis of the complex showed 16% weight loss at approximately 230 °C (Fig. 3f).”

4. The stability results are overly long and insufficiently structured. The sections: “Stability of the Host–Guest Complex of P5A and HMDI Against Water Vapour”, “Water Stability Under Harsher Conditions” and “Thermal Stability” could be merged into a single section entitled: “Stability of the Crystalline Host–Guest Complex of P5A and HMDI Against Water and

Temperature"
This reorganization would significantly improve clarity, reduce redundancy, and enhance readability.

5. Throughout the stability studies, the formation (or absence) of an "insoluble" product is mentioned (e.g., after exposure of neat HMDI to water vapour). The term "insoluble" lacks precision. Insoluble in which solvents? The authors must specify the solvent screening conditions.

6. Figure 3 is overloaded and difficult to interpret. It should be divided into three separate figures: (a), (b–h, k) and (i–j).
In addition:
- Photographs placed on arrows in Fig. 3b should be removed.
- The photograph of the complex in the solid state should be replaced by the image currently on the arrow.
- The crystal structure should be relocated and associated with the NMR spectrum in Fig. 3d.
- Fig. 3b could reasonably be moved to the Supporting Information.

7. The manuscript inconsistently refers to the same species as "P5A–HMDI co-crystal," "P5A–HMDI complex crystal," and "crystalline P5A–HMDI complex." A single, consistent nomenclature must be adopted throughout.

8. The term "deactivation" is repeatedly used to describe the reaction of HMDI with water. While understandable in the field of polymers, it is misleading here and potentially confusing given the "activation" process of pillararenes. The term "degradation" would be more chemically accurate in this article.

9. The degradation mechanism presented in Fig. 3a is unlikely to represent the dominant pathway. The major pathway presumably involves hydrolysis of a single isocyanate group to yield a mono-NCO/mono-NH_2 intermediate, followed by self-reaction and polymer formation. The scheme and accompanying discussion should be revised accordingly.

10. The terms "activated P5A crystal," "pristine P5A," and "crystalline P5A" are not clearly defined. The authors must explicitly clarify the nature of each material and specify which form is used in each experiment.

11. Statements such as "suggesting perfect protection was not achieved" or "the majority of the isocyanate groups" lack quantitative precision.

12. The paragraph discussing improved thermal stability due to crystalline packing and 1D channel structures should be moved to the section describing the X-ray crystallographic analysis, where it logically belongs.

13. The section title "Deprotection of the Supramolecular Protector" is conceptually incorrect: it is the bis-isocyanate that is deprotected, not the pillararene. A more appropriate title would be: "Deprotection of the Bis-Isocyanate in Solution" or "Removal of the Supramolecular Protecting Host in Solution". Additionally, the deprotection study was performed in DCM, whereas polyurethane synthesis was conducted in toluene. For coherence and practical relevance, the deprotection study should ideally be carried out in toluene.

14. Certain sentences (e.g., regarding improved handling due to reduced volatilization and degradation under ambient conditions) are redundant and could be removed to streamline the manuscript.

15. The modest recovery yield of P5A (73%) significantly impacts the practicality of the method. The authors should discuss the origin of this loss and propose strategies to improve recyclability.

16. The TOC graphic could be clarified as follows:
- "Supramolecular Protection of Isocyanates from Water" should be placed above the solid image on the right.
- The rightward arrow should be labeled "Solvent evaporation."
- The dissolution arrow should read "Facile deprotection through dissolution."
- The structure of the complex should be placed beneath the solid-state image.

In conclusion, the work presents an elegant and conceptually stimulating supramolecular strategy for solid-state protection of reactive monomers. With improved rigor, clarity, and structural organization, the manuscript would constitute a strong contribution suitable for publication in a high-impact journal.

Reviewer #3

(Remarks to the Author)
The authors report the use of a pillararene to protect diisocyanates from hydrolysis in the solid state. The results overall are convincing and impressive and will be of use to a wide readership in supramolecular chemistry, mechanically interlocked molecules and polymer synthesis.

The authors use hexamethylene diisocyanate as the representative diisocyanate. This raises the question of whether the author's approach is applicable to longer chain diisocyanates as presumably the isocyanate groups would protrude from the end of the pillararene and are less protected from hydrolysis?

The authors state that "1:1 complexation was confirmed" based on integrals, presumably after decomplexation? Normally I

would anticipate the use binding constants and Job plots to establish the ratio of binding. Has this been done?

Indeed the behaviour of the P5A-HMDI complex in different solvents is unclear even to someone who works on these types of materials. Presumably HMDI is bound by P5A in CHCl3 but not in CH2Cl2 and toluene? My assumption is based on the formation of the P5A-HMDI complex in CHCl3 but it would be useful to see the NMR in CHCl3. This behaviour would be consistent with other P5A/guest behaviour. I can't tell from the text whether the authors suggest for cyclohexane solutions. Can the authors make the solution phase behaviour in different solvents absolutely clear, preferably early in the text?

The authors state "The powder X-ray diffraction profile of the obtained solid showed crystallinity, and the pattern corresponding to the individual P5A disappeared and a new set of peaks appeared, indicating the formation of a crystalline host–guest complex (Supplementary Fig. 2)." The authors can say more than this as they also report the single crystal structure of the P5A-HMDI complex and compare the calculated and experimental PXRD patterns which confirms the identity of the powder.

A related paper has been published, presumably while the authors were writing their study - Nat. Commun., 2026, 17, 340. Although this other paper doesn't not impact the significance of the authors' study is does provide a useful parallel and would be a valuable comparison.

Overall this is an interesting study and if the points raised above can be addressed I recommend publication in Nature Communications.

Version 1:

Reviewer comments:

Reviewer #1

(Remarks to the Author)
I recommend the paper to be accepted. The authors have addressed the points that have been raised in my report.

Reviewer #2

(Remarks to the Author)
In this revised version of the manuscript, the authors have addressed all the Referees' comments and have significantly improved both the rigor and the clarity of the text. In addition, further experiments have been successfully carried out to strengthen the applicability of the method. I have no further comments and recommend the publication of this work.

Reviewer #3

(Remarks to the Author)
The authors have done an excellent job of answering this reviewers comments, and those of the other reviewers (in my opinion). I recommend publication without further changes.

Open Access This Peer Review File is licensed under a Creative Commons Attribution 4.0 International License, which permits use, sharing, adaptation, distribution and reproduction in any medium or format, as long as you give appropriate credit to the original author(s) and the source, provide a link to the Creative Commons license, and indicate if changes were made.
In cases where reviewers are anonymous, credit should be given to 'Anonymous Referee' and the source.
The images or other third party material in this Peer Review File are included in the article's Creative Commons license, unless indicated otherwise in a credit line to the material. If material is not included in the article's Creative Commons license and your intended use is not permitted by statutory regulation or exceeds the permitted use, you will need to obtain permission directly from the copyright holder.
To view a copy of this license, visit https://creativecommons.org/licenses/by/4.0/

According to the comments from three reviewers, we have revised our manuscript and supporting information. We would like to explain these corrections point-by-point as follows:

Reviewer 1

Comment: The manuscript describes the successful implementation of supramolecular protection strategy towards isocyanate compounds that are known to be unstable in the presence of water (also atmospheric moisture). The non-covalent protection of isocyanates occurs only in the crystalline state through their inclusion into the macrocyclic cavities of per-ethylated pillar[n]arene macrocycles. Such solid state host-guest assemblies are highly hydrophobic providing stabilization and a mean for long-term storage for isocyanate compounds. The deprotection is achieved simply by dissolution the host-guest crystals, as no complexation in the solution has been observed by NMR. The method itself has some limitation, as good host-guest complementarity is required between specific isocyanate guest and macrocyclic host. As the authors discuss, more bulky isocyanates are not suitable guests for either pillar[5]arene or pillar[6]arene and thus could not be protected. Still, in future it seems to be possible to match even bulkier isocyanates with suitable hosts, not necessarily of pillar[n]arene family. Taking into account the importance of di-isocyanate compounds in the production of polyurethane polymers, the demonstrated potential for practical application, together with possible extension of supramolecular protection concept onto other water-sensitive reagents, I would recommend the paper to be published after some minor revision (see below).

Answer: We appreciate the strong recommendation of the reviewer to publish our paper.

Comment 1: Label of Fig. 3… i, j, X-ray crystal structure of the P5A–HMDI complex in the ab plane (i, space filling model, the solvent molecules have been removed for clarity) and bc plane (j, capped sticks model, the solvent molecules and hydrogen atoms have been removed for clarity).

The crystal structure model of P5A–HMDI as appears from CIF file does not comprise any solvent molecules to be omitted. The same comment concerns label of Fig.5d,e and crystal structure of P6A-MDI. Please, correct.

Answer 1: We appreciate the reviewer's comment. According to the reviewer's comment, we have removed the comment from Fig. 3i,j (in revision Fig. 5a,b) and Fig. 5d,e (in revision Fig. 7d,e).

Comment 2: The P5A–HMDI crystal structure model has misplaced hydrogen atoms on C01P carbon atom, which is part of disordered ethyl group. Hence, the erroneous formula for pillar[5]arene given as C55 H69.41 O10 in CIF file. The proper formula should be C55 H70 O10.

The handling of disorder of HMDI molecule included in the P5A cavity is far from optimal. Very harsh EADP constraints and ISOR restraints have been used excessively, that can implement a bias in the structure model. EADP and ISOR should be used very carefully when other measures have been unsuccessful (combination of SIMU and RIGU) as the last resort only. The minor component of disorder can also be refined isotropically. The same applies for disordered O-ethyl substituents of pillar[5]arene. The implementation of all restraints and constraints should be clearly explained in the manuscript text (Supp. info). The problematic lines in the res file are, for example:

EADP N5A N5
EADP C5A C5
EADP O13A O13
EADP C8A C8
EADP N12A N12

EADP C6A C6
EADP O24 O24A
EADP C9A C3
EADP C3 C9
EADP C9 C9A
EADP C3A C3
SOR 0.01 0.02 O13A O13 C5 C5A N5 N5A C9A C9 C3 C3A C1A C1 C2A C2 C4A C4 =
C8A C8 N12 N12A C6 C6A O24 O24A

Overall, the structure should be re-refined with correct placement of hydrogen atoms and reconsidered disorder modelling. The new CIF file should be re-deposited in the CSD.

Answer 2: We appreciate the valuable comment of the reviewer. We re-examined the structural model of P5A–HMDI and constructed an updated model with less disorder, because the disorder model in the initial refinement was obtained only by using harsh constraints/restraints as the reviewer pointed out. Now we obtained satisfactorily reliable model, which we believe suitable for publication. The updated CIF was deposited in the CSD.

Comment 3: The refinement strategy for symmetry-related disorder of MDI guest in the pillar[6]arene cavity could be improved. Instead of the combination of DELU and ISOR as used by authors, the SIMU/RIGU should be more appropriate.
DELU 0.003 C34 > LAST
ISOR 0.015 C34 > LAST
Also, many DFIX and SADI can be simply substituted by AFIX 66 for the geometry of aromatic rings.
DFIX 1.39 C35 C36 C36 C37 C37 C38 C38 C39 C39 C40 C40 C35
SADI C35 C37 C36 C38 C37 C39 C38 C40 C39 C35 C40 C36
DFIX 1.39 C42 C43 C43 C44 C44 C45 C45 C46 C46 C47 C47 C4
SADI C42 C44 C43 C45 C44 C46 C45 C47 C46 C42 C47 C43

Answer 3: We thanks this reviewer for important comment. According to the suggestion by the reviewer, the structure of EtP6A-MDI was re-refined using SIMU/RIGU restraints instead of DELU/ISOR. However, we did not use AFIX66 rigid group constraints because use of AFIX66 is not consistent with the policy of avoiding too many harsh constraints/restraints. The updated CIF was deposited in the CSD.

<u>Reviewer 2</u>

Comment: The manuscript describes the use of pillararenes as supramolecular hosts to protect bis-isocyanates in the solid state, enabling their long-term storage. The authors convincingly demonstrate, through NMR, IR, and single-crystal X-ray diffraction analyses, that the bis-isocyanates are encapsulated within the cavity of the pillararenes, which assemble into hydrophobic channels in the crystalline state. This supramolecular protection strategy enables the stabilization of highly moisture-sensitive species such as isocyanates by shielding them from ambient humidity.

A key advantage over conventional covalent protection strategies lies in the reversibility of the process: upon dissolution, supramolecular deprotection occurs rapidly and quantitatively, without the need for additional reagents, thereby restoring the intrinsic reactivity of the bis-isocyanates. The authors further demonstrate the synthetic utility of this concept through its application to polyurethane synthesis.

Overall, the study is convincing and will be of interest to researchers in supramolecular chemistry and

beyond. Regarding conceptual originality, to the best of my knowledge, there are no closely related reports involving supramolecular protection using polyaromatic cavity-based macrocycles such as pillararenes. However, the broader concept of solid-state stabilization via host–guest chemistry is less unprecedented in the context of cyclodextrins, which have been extensively investigated for stabilizing and suppressing the reactivity of neutral guests in the solid state.

At first glance, the generality of the concept appears somewhat limited. Pillar[5]arene derivatives can encapsulate only linear bis-isocyanates within a quite narrow chain-length window. Larger oligomers such as pillar[6]arenes accommodate a broader range of guests, yet guest size and geometry remain constrained by cavity dimensions. In addition, pillar[6]arenes remain comparatively expensive to produce. Expanding the methodology to pillararenes of different cavity sizes and, critically, optimizing host recyclability (the reported recovery yield of 73% remains modest) will be essential steps toward improving the generality and practical viability of the approach.

Despite these limitations, I believe the manuscript merits publication in a journal such as Nature Communications, provided the authors address the following points:

Answer: We appreciate your evaluation of our research and the recommendation to publish in Nature Communications. To overcome the narrow chain-length window, we have demonstrated the concept using octadecyl isocyanate as a longer-chain example and have included these results in the revised manuscript as follows:

Interestingly, P5A also exhibited a protective effect against water vapour for linear alkyl isocyanate with longer chain, octadecyl isocyanate (ODI) (Supplementary Figs. 29 and 30). Previous studies have shown that P5A can encapsulate linear alkanes with chain lengths exceeding that of a single P5A cavity through inclusion by multiple P5A units, forming 1D channel structures.[30] In the case of ODI, the formation of a similar 1D channel structure was confirmed by PXRD analysis (Supplementary Figs. 31), suggesting that multiple P5A molecules encapsulate ODI and protect it from water vapour. Thus, we demonstrate that this supramolecular protection strategy is applicable to linear alkyl isocyanate compounds over a wide range of alkyl chain lengths.

Regarding the recyclability, the recovery yield was improved to 85% by revisiting and optimizing the reprecipitation conditions. To further improve the generality of this approach, we plan to apply pillar[*n*]arenes with different cavity sizes. This is our next target. Thanks again the reviewer for pointing out the important point.

Comment 1: The description of the solution-state complexation behavior is imprecise and at times contradictory. For example, the Introduction states: “because complexation only occurs in the crystalline state”, whereas the caption of Figure 1 reads: “Because the binding affinity of the complex is weak in the reaction solvent…”. These statements are not inherently incompatible but lack sufficient clarification. It is evident that complexation is solvent-dependent: in DCM, no complexation is observed due to competitive inclusion of the solvent, whereas in $CHCl_3$, weak complexation occurs because the solvent is too bulky to fit efficiently within the cavity. Similar ambiguities appear in the discussion of pillar[6]arene in different solvents (toluene, DCM, etc.). In other words, the authors should provide a more rigorous and solvent-specific description of binding behavior and avoid overly general statements.

Answer 1: We appreciate the reviewer’s valuable comment. Yes, as the reviewer mentioned, the inclusion behavior of pillar[*n*]arenes in solution is solvent-dependent. In case of P5A, in DCM, no complexation is observed due to competitive inclusion of the solvent, whereas in $CHCl_3$, weak complexation occurs because the solvent is too bulky to fit efficiently within the cavity. Similar ambiguities appear in the discussion of pillar[6]arene in different solvents (toluene, DCM, etc.). According to the reviewer’s suggestion, we have

provided a more rigorous and solvent-specific description of binding behavior and avoided overly general statements in the revision.

According to the reviewer's comment, we have revised the manuscript to clarify these points as follows:

Importantly, although the host–guest complex is stabilized in the crystalline state, dissolution in an appropriate solvent rapidly induces dissociation of the complex, enabling facile deprotection (Fig. 1c).

Because chloroform is bulkier than the cavity of P5A, it does not compete with HMDI for inclusion in solution or during crystallization, allowing preferential encapsulation of HMDI during crystal formation. The obtained solid was then washed with cyclohexane to remove uncomplexed HMDI because bulky cyclohexane does not compete with the guest of P5A and can dissolve only HMDI.

Unlike chloroform, dichloromethane acts as a competitive guest solvent and rapidly dissociates the P5A–HMDI complex; therefore, the resulting integral ratio reflects the host–guest stoichiometry in the crystalline complex.

The deprotection behavior can be readily controlled by appropriate solvent selection. For example, in systems based on P5A, dichloromethane, whose molecular size matches the cavity of P5A, is known to induce competitive inclusion and thereby rapidly release guests encapsulated in the crystalline state.

Importantly, deprotection of the isocyanate was achieved simply by dissolving the crystalline complex in the reaction solvent at the reaction temperature (Supplementary Fig. 16 and Supplementary Note 2). Unlike dichloromethane, the bulkier toluene does not effectively induce competitive inclusion with P5A and therefore provides slower release kinetics; however, dissociation of the complex proceeded sufficiently under the reaction conditions.

Comment 2: Figure 1 lacks clarity and scientific rigor in its current form:
- In step e, the guest should be indicated on the reaction arrow, and polymer separation should be explicitly depicted.
- In step c, the solvent (rather than the complex) should appear above the arrow.
- The notation "R′" is inappropriate; "X" would be clearer.
- The central image unnecessarily complicates the scheme and applies only to pillar[5]arenes.

A complete redesign of this figure is recommended.

Answer 2: We thank the reviewer for helpful comments. To improve the clarity and scientific rigor of Figure 1, we have revised the figure according to the reviewer's comments as follows:

- In step e, the addition of guest and polymer separation were described with the arrow.
- In step c, the solvent appeared above the arrow and in the flask.
- The notation "X" was used instead of "R′" in the polyurethane synthesis part.
- The central image was removed.
- In step d, "Dissociation" was used instead of "No Complexation" to enhance the rigorous.

Comment 3: "Fig. 3f" should be corrected to "Fig. 3k" in the sentence:
"Thermogravimetric analysis of the complex showed 16% weight loss at approximately 230 °C (Fig. 3f)."

Answer 3: We thank this reviewer for comment. We have corrected "Fig. 3f" to "Fig. 3k" (in revision Fig. 4h) in the corresponding sentence.

Comment 4: The stability results are overly long and insufficiently structured. The sections: "Stability of the Host–Guest Complex of P5A and HMDI Against Water Vapour", "Water Stability Under Harsher Conditions" and "Thermal Stability" could be merged into a single section entitled: "Stability of the Crystalline Host–Guest Complex of P5A and HMDI Against Water and Temperature"
This reorganization would significantly improve clarity, reduce redundancy, and enhance readability.

Answer 4: We appreciate the valuable comment of the reviewer. According to the reviewer's suggestion, we have merged the three sections "Stability of the Host–Guest Complex of P5A and HMDI Against Water Vapour," "Water Stability Under Harsher Conditions," and "Thermal Stability" into a single section entitled "Stability of the Crystalline Host–Guest Complex of P5A and HMDI Against Water and Temperature."

Comment 5: Throughout the stability studies, the formation (or absence) of an "insoluble" product is mentioned (e.g., after exposure of neat HMDI to water vapour). The term "insoluble" lacks precision. Insoluble in which solvents? The authors must specify the solvent screening conditions.

Answer 5: Thanks for your suggestion. The product obtained after exposure of neat HMDI to water vapour was confirmed to be insoluble in a range of solvents, including water, DMSO, DMF, MeOH, acetone, THF, dichloromethane, chloroform, and *n*-hexane. Instead of using only the ambiguous term "insoluble," we have clarified the used solvents as follows;

After 3 days, a white product was obtained that was hardly soluble in a wide range of solvents, including water, DMSO, DMF, MeOH, acetone, THF, dichloromethane, chloroform, and *n*-hexane.

Comment 6: Figure 3 is overloaded and difficult to interpret. It should be divided into three separate figures: (a), (b–h, k) and (i–j).
In addition:
- Photographs placed on arrows in Fig. 3b should be removed.
- The photograph of the complex in the solid state should be replaced by the image currently on the arrow.
- The crystal structure should be relocated and associated with the NMR spectrum in Fig. 3d.
- Fig. 3b could reasonably be moved to the Supporting Information.

Answer 6: Thank you for important comment to improve our paper. According to the reviewer's suggestion, we have divided Fig. 3 into Fig. 3–5 and reorganized it to improve clarity and readability. Specifically, we have made the following revisions:

- Figure 3 has been revised to include only data related to the degradation of HMDI.
- Figure 4 now presents only the results associated with the stability evaluation of the crystalline P5A–HMDI complex.
- The original Fig. 3b has been moved to Supplementary Figs. 6 and 7.
- The single-crystal structure of the P5A–HMDI complex has been moved to Fig. 5 and associated with the NMR spectrum shown in Fig. 4c (formally Fig. 3d) as follows:

X-ray crystallographic analysis revealed that the HMDI molecule was encapsulated within the cavity of P5A at a 1:1 molar ratio, consistent with the integral ratio observed in the ^{1}H NMR spectrum (Fig. 4c), and that the P5A units assembled into a one-dimensional (1D) channel structure (Fig. 5a and b).

Comment 7: The manuscript inconsistently refers to the same species as "P5A–HMDI co-crystal," "P5A–HMDI complex crystal," and "crystalline P5A–HMDI complex." A single, consistent nomenclature must be adopted throughout.

Answer 7: We thank the reviewer's comment. We have unified the terminology as "crystalline P5A–HMDI complex" throughout the manuscript.

Comment 8: The term "deactivation" is repeatedly used to describe the reaction of HMDI with water. While understandable in the field of polymers, it is misleading here and potentially confusing given the "activation" process of pillararenes. The term "degradation" would be more chemically accurate in this article.

Answer 8: We appreciate the reviewer's comment. According to the reviewer's comment, we have unified the terminology to "degradation" to describe the reaction of HMDI with water.

Comment 9: The degradation mechanism presented in Fig. 3a is unlikely to represent the dominant pathway. The major pathway presumably involves hydrolysis of a single isocyanate group to yield a mono-NCO/mono-NH_2 intermediate, followed by self-reaction and polymer formation. The scheme and accompanying discussion should be revised accordingly.

Answer 9: We thank the reviewer for the important comment. In the revised manuscript, we have corrected the reaction description to reflect the more plausible pathway involving formation of a mono-NCO/mono-NH_2 intermediate followed by its self-reaction to give polymeric products. Accordingly, the degradation scheme in Fig. 3b (formerly Fig. 3a) has also been revised.

Comment 10: The terms "activated P5A crystal," "pristine P5A," and "crystalline P5A" are not clearly defined. The authors must explicitly clarify the nature of each material and specify which form is used in each experiment.

Answer 10: We thank the reviewer for this valuable comment. In our original manuscript, the term "activated P5A crystal" was used to describe guest-free P5A crystals, following common description in gas and vapor adsorption studies, where "activation" refers to removal of guest molecules from the cavity prior to adsorption measurements. However, we agree that this terminology may cause confusion in the present context. To avoid ambiguity, we have removed the term "activated" and instead explicitly state in the Experimental section when guest-free P5A was used. Similarly, the term "pristine P5A" was originally used to indicate P5A prior to exposure to water vapor. As this distinction can be clearly conveyed without introducing additional terminology, we have removed the term "pristine" throughout the manuscript. In the revised manuscript, we now explicitly clarify the state of P5A used in each experiment to ensure consistency and clarity.

To clarify the reason why the isocyanate compound was effectively protected from water, we measured the adsorption of water using crystalline P5A without any guest molecules in its cavity.

Comment 11: Statements such as "suggesting perfect protection was not achieved" or "the majority of the isocyanate groups" lack quantitative precision.

Answer 11: We thank this reviewer for important comment. We agree that statements such as "suggesting perfect protection was not achieved" and "the majority of the isocyanate groups" lack quantitative precision. We have therefore performed a quantitative evaluation based on the integral ratio of P5A and HMDI in the ^{1}H NMR measurements before and after immersing boiling water. The degradation ratio can

be calculated from the integration ratio of a HMDI proton (H_a) to an aromatic proton of P5A (H_A) (from 3.88 to 3.70, Fig. 3d and 3g).

Degradation ratio (%) = 3.70 / 3.88 × 100 = 95%

We have added the quantitative result in the manuscript as follows:

However, these results indicate that most of the isocyanate groups were still protected from degradation in the crystalline state (95% from ^{1}H NMR integral ratio, Supplementary Fig. 11), even after immersion in boiling water.

In addition, the calculation method has described in Supplementary Fig. 11 as follows:

The degradation ratio was calculated from the integration ratio of a HMDI proton (H_a) to an aromatic proton of P5A (H_A) (from 3.88 to 3.70). Degradation ratio (%) = 3.70 / 3.88 × 100 = 95%

Comment 12: The paragraph discussing improved thermal stability due to crystalline packing and 1D channel structures should be moved to the section describing the X-ray crystallographic analysis, where it logically belongs.

Answer 12: We thank the reviewer for this important comment. As suggested by the reviewer, we have moved the paragraph discussing the improved thermal stability to the section describing the X-ray crystallographic analysis. In addition, we have clarified the origin of the improved thermal stability by discussing the confinement effect of the isocyanate species within the 1D channels of P5A.

Indeed, our previous study demonstrated that the thermal stability of polyethylene oxide (PEO) confined within the 1D channels of P5A was significantly enhanced compared with that of neat PEO. This enhancement was attributed to restricted molecular motion of PEO within the channels, as the polymer chains fit snugly into the 1D cavity of P5A (*Nat. Commun.* **10**, 479 (2019)). Based on this precedent, we consider that a similar confinement effect contributes to the improved thermal stability observed in the present system. The manuscript has been revised accordingly as follows:

Furthermore, the formation of 1D channel structure would be a one of key factors contributing to the improved thermal stability. Based on our previous studies, molecules confined within the 1D channels of P5A crystals exhibit significantly restricted molecular motion compared with the neat molecules because they fit into the P5A channels.[32] Consequently, the confined molecules show enhanced thermal stability. In the present study, we therefore consider that confinement of the isocyanate species within the 1D channel structure of the P5A crystal is responsible for the remarkable improvement in thermal stability observed in the crystalline state, which was comparable to the typically covalent stabilization.

Comment 13: The section title "Deprotection of the Supramolecular Protector" is conceptually incorrect: it is the bis-isocyanate that is deprotected, not the pillararene. A more appropriate title would be: "Deprotection of the Bis-Isocyanate in Solution" or "Removal of the Supramolecular Protecting Host in Solution". Additionally, the deprotection study was performed in DCM, whereas polyurethane synthesis was conducted in toluene. For coherence and practical relevance, the deprotection study should ideally be carried out in toluene.

Answer 13: We appreciate this reviewer for helpful comment to improve our paper. We have revised the section title to "Removal of the Supramolecular Protecting Host in Solution" to avoid ambiguity regarding the species being deprotected. With regard to the solvent choice, the deprotection study was performed in DCM because its molecular size matches the cavity of P5A and promotes competitive inclusion, facilitating rapid guest release. In contrast, toluene is less effective in inducing competitive inclusion with P5A and

provides slower release kinetics. For this reason, DCM was selected to clearly demonstrate the deprotection behavior. Nevertheless, polyurethane synthesis was successfully carried out in toluene under heating conditions (reaction temperature), indicating that guest release also proceeds under these conditions. We have clarified this point in the revised manuscript to improve consistency and practical relevance as follows:

Removal of the Supramolecular Protecting Host in Solution

We next investigated the deprotection process of the supramolecularly protected HMDI. The deprotection behavior can be readily controlled by appropriate solvent selection. For example, in systems based on P5A, DCM, whose molecular size matches the cavity of P5A, is known to induce competitive inclusion and thereby rapidly release guests encapsulated in the crystalline state.

Polyurethane Synthesis

One of the important roles of isocyanate groups is as a precursor for polyurethane synthesis. Therefore, we attempted to synthesise polyurethane from supramolecularly protected HMDI (Fig. 6). The crystalline P5A–HMDI complex and triethylene glycol were dissolved in toluene and stirred at 90 °C for 3 h in the presence of a dibutyltin dilaurate (DBTDL) catalyst. After the reaction, a white solid precipitated. The toluene phase was removed, and the precipitate was dissolved in dimethyl sulfoxide-d6 for 1H NMR analysis. The ^{1}H NMR spectra clearly showed proton signals corresponding to urethane linkages, indicating successful polymerisation (Supplementary Fig. 17).

Importantly, deprotection of the isocyanate was achieved simply by dissolving the crystalline complex in the reaction solvent at the reaction temperature (Supplementary Fig. 16 and Supplementary Note 2). Unlike dichloromethane, the bulkier toluene does not effectively induce competitive inclusion with P5A and therefore provides slower release kinetics; however, dissociation of the complex proceeded sufficiently under the reaction conditions.

Comment 14: Certain sentences (e.g., regarding improved handling due to reduced volatilization and degradation under ambient conditions) are redundant and could be removed to streamline the manuscript.

Answer 14: We thank this reviewer for the valuable comment. We have removed these sentences from the main text to improve conciseness.

Comment 15: The modest recovery yield of P5A (73%) significantly impacts the practicality of the method. The authors should discuss the origin of this loss and propose strategies to improve recyclability.

Answer 15: We thank the reviewer for this important comment. We consider that the relatively low recovery yield was mainly due to material loss during the reprecipitation process. As commonly practiced, reducing the amount of good solvent used during reprecipitation and using a cold poor solvent are effective ways to minimize such losses. Therefore, we repeated the polyurethane synthesis under the same conditions and optimized the reprecipitation procedure. As a result, the recovery yield improved to 85%. The revised manuscript includes the improved recovery yield, and the solvent volumes used in the reprecipitation process have been described in detail in the Supporting Information as follows:

After polyurethane synthesis, the supernatant solution and the toluene used for washing were evaporated. The residue was redissolved in dichloromethane and passed through a membrane filter to remove insoluble impurities, and then the filtrate was concentrated. The residue was redissolved in 2 mL of dichloromethane

and reprecipitated by addition to 40 mL of cold methanol. The solution was decanted and the residue was dried under vacuum to afford white solid P5A (214.9 mg, 85% recovery).

Comment 16: The TOC graphic could be clarified as follows:
- "Supramolecular Protection of Isocyanates from Water" should be placed above the solid image on the right.
- The rightward arrow should be labeled "Solvent evaporation."
- The dissolution arrow should read "Facile deprotection through dissolution."
- The structure of the complex should be placed beneath the solid-state image.

Answer 16: We thank this reviewer for helpful comment. Based on the reviewer's suggestion, we have revised the TOC graphic.

Comment: In conclusion, the work presents an elegant and conceptually stimulating supramolecular strategy for solid-state protection of reactive monomers. With improved rigor, clarity, and structural organization, the manuscript would constitute a strong contribution suitable for publication in a high-impact journal.

Answer: We appreciate your thorough evaluation of our research. Following the reviewer's suggestions, we have carefully revised the manuscript to improve its rigor, clarity, and structural organization.

Reviewer 3

Comment: The authors report the use of a pillararene to protect diisocyanates from hydrolysis in the solid state. The results overall are convincing and impressive and will be of use to a wide readership in supramolecular chemistry, mechanically interlocked molecules and polymer synthesis.

Answer: We appreciate the strong recommendation of the reviewer to publish our paper.

Comment 1: The authors use hexamethylene diisocyanate as the representative diisocyanate. This raises the question of whether the author's approach is applicable to longer chain diisocyanates as presumably the isocyanate groups would protrude from the end of the pillararene and are less protected from hydrolysis?

Answer 1: We thank the reviewer for this important comment, which helped us improve the manuscript. Long-chain diisocyanates are not commercially available; therefore, we used a mono-isocyanate (octadecyl isocyanate, ODI) as a representative example. Previous studies have shown that P5A can form crystalline host–guest complexes with linear alkanes longer than the cavity size of a single P5A molecule through encapsulation by multiple P5A units, resulting in 1D channel structures (*Chem Commun.* **53**, 8577-8580 (2017)). Based on this previous report, we thought that long-chain alkyl isocyanates could likewise be confined within such 1D channel structures and protected from water vapour. Consistent with this expectation, PXRD analysis confirmed the formation of a 1D channel structure with octadecyl isocyanate, and the resulting crystalline complex was found to be stable against water vapour. These results have been included in the revised manuscript as follows:

Interestingly, P5A also exhibited a protective effect against water vapour for linear alkyl isocyanate with longer chain, octadecyl isocyanate (ODI) (Supplementary Figs. 29 and 30). Previous studies have shown that P5A can encapsulate linear alkanes with chain lengths exceeding that of a single P5A cavity through inclusion by multiple P5A units, forming 1D channel structures.[30] In the case of ODI, the formation of a similar 1D channel structure was confirmed by PXRD analysis (Supplementary Figs. 31), suggesting that

multiple P5A molecules encapsulate ODI and protect it from water vapour. Thus, we demonstrate that this supramolecular protection strategy is applicable to linear alkyl isocyanate compounds over a wide range of alkyl chain lengths.

Comment 2: The authors state that "1:1 complexation was confirmed" based on integrals, presumably after decomplexation? Normally I would anticipate the use binding constants and Job plots to establish the ratio of binding. Has this been done?

Answer 2: We thank the reviewer for this important comment. In this study, we aimed to determine the stoichiometry of the host–guest complex in the crystalline state rather than in solution. Therefore, instead of determining binding constants or performing Job plot analyses, which are typically used to evaluate solution-phase binding behavior, we estimated the stoichiometry of the crystalline complex using three independent approaches. First, the integral ratio obtained from the ^{1}H NMR spectrum in CD_2Cl_2 after decomplexation was used. Because CD_2Cl_2 acts as a competitive guest solvent and rapidly dissociates the P5A–HMDI complex, the resulting integral ratio reflects the host–guest composition in the crystalline complex. Second, thermogravimetric analysis showed a weight loss corresponding to approximately one equivalent of HMDI at around 230 °C, which supports formation of a 1:1 host–guest complex. Third, single-crystal X-ray diffraction analysis clearly revealed the formation of a 1:1 P5A–HMDI complex in the solid state. Based on these consistent results, we concluded that the complex forms with a 1:1 stoichiometry in the crystalline phase. We have clarified this point in the revised manuscript.

Comment 3: Indeed the behaviour of the P5A-HMDI complex in different solvents is unclear even to someone who works on these types of materials. Presumably HMDI is bound by P5A in $CHCl_3$ but not in CH_2Cl_2 and toluene? My assumption is based on the formation of the P5A-HMDI complex in $CHCl_3$ but it would be useful to see the NMR in $CHCl_3$. This behaviour would be consistent with other P5A/guest behaviour. I can't tell from the text whether the authors suggest for cyclohexane solutions. Can the authors make the solution phase behaviour in different solvents absolutely clear, preferably early in the text?

Answer 3: Thank you for the helpful comments. The solution-phase behavior of the P5A–HMDI complex depends strongly on solvent choice. In $CHCl_3$, complex formation was confirmed by ^{1}H NMR spectroscopy. In contrast, CH_2Cl_2 acts as a competitive guest solvent and induces dissociation of the complex. Toluene does not effectively compete for the P5A cavity but allows guest release under the reaction temperature used for polymerization. We did not measure the ^{1}H NMR spectrum in cyclohexane because P5A is not soluble in this solvent. Instead, cyclohexane was used to remove uncomplexed HMDI, as it readily dissolves free HMDI but does not compete for inclusion in the P5A cavity due to its bulky structure. Rather than introducing all solvent effects at once, we clarified the solvent-dependent behavior at the relevant points in the manuscript where each solvent is discussed in context, to maintain readability while improving clarity.

Preparation of the Host–Guest Complex of Pillar[5]arene and Hexamethylene Diisocyanate

We first used hexamethylene diisocyanate (HMDI) as the target isocyanate compound and pillar[5]arene (P5A) crystal as the 'supramolecular protector' (Fig. 2). Since the cavity size of P5A (4.7 Å) is suitable for linear alkyl chain, a host-guest complex of P5A and HMDI was prepared by simply concentrating a chloroform solution of the two components in 1 to 1.1 molar ratio (Fig. 2a, details in Methods). Because chloroform is bulkier than the cavity of P5A, it does not compete with HMDI for inclusion in solution or during crystallization, allowing preferential encapsulation of HMDI during crystal formation. The obtained solid was then washed with cyclohexane to remove uncomplexed HMDI because bulky cyclohexane does not compete with the guest of P5A and can dissolve only HMDI. Proton signals from both P5A and HMDI

were observed by 1H NMR in dichloromethane-d2 (Fig. 2b) even after uncomplexed HMDI was removed with cyclohexane, indicating the complexation of P5A and HMDI. Unlike chloroform, dichloromethane acts as a competitive guest solvent and rapidly dissociates the P5A–HMDI complex; therefore, the resulting integral ratio reflects the host–guest stoichiometry in the crystalline complex.

Removal of the Supramolecular Protecting Host in Solution

We next investigated the deprotection process of the supramolecularly protected HMDI. The deprotection behavior can be readily controlled by appropriate solvent selection. For example, in systems based on P5A, DCM, whose molecular size matches the cavity of P5A, is known to induce competitive inclusion and thereby rapidly release guests encapsulated in the crystalline state.

Polyurethane Synthesis

Importantly, deprotection of the isocyanate was achieved simply by dissolving the crystalline complex in the reaction solvent at the reaction temperature (Supplementary Fig. 16 and Supplementary Note 2). Unlike dichloromethane, the bulkier toluene does not effectively induce competitive inclusion with P5A and therefore provides slower release kinetics; however, dissociation of the complex proceeded sufficiently under the reaction conditions.

Comment 4: The authors state "The powder X-ray diffraction profile of the obtained solid showed crystallinity, and the pattern corresponding to the individual P5A disappeared and a new set of peaks appeared, indicating the formation of a crystalline host–guest complex (Supplementary Fig. 2)." The authors can say more than this as they also report the single crystal structure of the P5A-HMDI complex and compare the calculated and experimental PXRD patterns which confirms the identity of the powder.

Answer 4: We thank the reviewer for this important comment. As the reviewer suggested, since we obtained the single-crystal structure of the P5A–HMDI complex, we are able to more directly confirm the identity of the crystalline phase. Therefore, we have added a statement to the manuscript clarifying that the experimental PXRD pattern agrees well with the pattern simulated from the single-crystal X-ray structure, as follows:

The powder X-ray diffraction (PXRD) profile of the obtained solid showed disappearance of the peaks corresponding to individual P5A and the appearance of a new set of reflections, indicating formation of a crystalline host–guest complex.

The experimental PXRD pattern is consistent with that simulated from the single-crystal X-ray structure of the P5A–HMDI complex (Supplementary Fig. 2).

Comment 5: A related paper has been published, presumably while the authors were writing their study - Nat. Commun., 2026, 17, 340. Although this other paper doesn't not impact the significance of the authors' study is does provide a useful parallel and would be a valuable comparison.

Answer 5: We thank the reviewer for this helpful suggestion. The paper pointed out by the reviewer involves the use of HMDI in a pillar[5]arene-based system; however, it does not address supramolecular protection or host–guest encapsulation of HMDI and therefore is not directly comparable to the present study from that perspective. Nevertheless, it highlights the importance of diisocyanates not only as building blocks for linear polyurethanes and polyureas but also as key components for the construction of network polymers. In this context, our work demonstrates a supramolecular protection strategy for diisocyanates, which is expected to be applicable not only to linear polymer synthesis but also to the design of network

polymers and gel materials. Accordingly, we have added a statement in the Conclusion to clarify this broader applicability as follows:

Given the growing use of isocyanates not only as monomers for polyurethanes and polyureas but also as dynamic crosslinkers in network polymers,[39,40] stabilization through host–guest encapsulation is expected to provide a broadly useful strategy in materials chemistry.

39 Liu, W., Yang, S., Huang, L., Xu, J., & Zhao, N., Dynamic covalent polymers enabled by reversible isocyanate chemistry. *Chem. Commun.* **58**, 12399-12417 (2022).

40 Guo, Y., Liu, Y., Yuan, C., Champness, N. R., Wang, J., & Yang, L., Unraveling positive deformation rate-elongation relationships in tough and highly stretchable ionogels under rapid deformation. *Nat. Commun.* **17**, 340 (2026).

Comment: Overall this is an interesting study and if the points raised above can be addressed I recommend publication in Nature Communications.

Answer: We appreciate the strong recommendation of the reviewer to publish our paper.

Changes made in the manuscript and supporting information are highlighted for clarity.

Finally, we would like to express our gratitude to you and reviewers for kind and worth comments. We strongly hope that this revised manuscript will be accepted for publication in your journal.

Sincerely yours,
Tomoki Ogoshi

According to the comments from three reviewers, we have revised our manuscript and supporting information. We would like to explain these corrections point-by-point as follows:

Reviewer #1 (Remarks to the Author):
I recommend the paper to be accepted. The authors have addressed the points that have been raised in my report.

Answer: We appreciate the strong recommendation of the reviewer to publish our paper.

Reviewer #2 (Remarks to the Author):
In this revised version of the manuscript, the authors have addressed all the Referees' comments and have significantly improved both the rigor and the clarity of the text. In addition, further experiments have been successfully carried out to strengthen the applicability of the method. I have no further comments and recommend the publication of this work.

Answer: We appreciate the strong recommendation of the reviewer to publish our paper.

Reviewer #3 (Remarks to the Author):
The authors have done an excellent job of answering this reviewers comments, and those of the other reviewers (in my opinion). I recommend publication without further changes.

Answer: We appreciate the strong recommendation of the reviewer to publish our paper.
